# Novel Synthetic Opioids (NSOs) and Their Evolving Crisis: Utilising NPS*finder*^®^ as a Real-Time Predictive Tool

**DOI:** 10.3390/ph19010017

**Published:** 2025-12-21

**Authors:** Elena Deligianni, Davide Arillotta, Alessandro Vento, John Martin Corkery, Georgios Papazisis, Antonis Goulas, Lisa Lione, Fabrizio Schifano

**Affiliations:** 1 Psychopharmacology, Drug Misuse and Novel Psychoactive Substances Research Unit, Hertfordshire Medical School, University of Hertfordshire, Hatfield AL10 9AB, UK; elena.deligian@gmail.com (E.D.); davide.arillotta@yahoo.it (D.A.); j.corkery@herts.ac.uk (J.M.C.); l.lione@herts.ac.uk (L.L.); 2 Laboratory of Clinical Pharmacology, School of Medicine, Aristotle University of Thessaloniki, 54124 Thessaloniki, Greece; papazisg@auth.gr; 3 Department of Neurosciences, Psychology, Drug Research, and Child Health, Section of Pharmacology and Toxicology, University of Florence, 50121 Florence, Italy; 4 Observatory on Addictions and Mental Health Department, ASL Roma 2, 00182 Rome, Italy; alessandrovento@gmail.com; 5 Clinical Research Unit, Special Unit for Biomedical Research and Education (SUBRE), School of Medicine, Aristotle University of Thessaloniki, 54124 Thessaloniki, Greece; 6 1st Laboratory of Pharmacology, School of Medicine, Aristotle University of Thessaloniki, 54124 Thessaloniki, Greece; agoulas@auth.gr

**Keywords:** novel psychoactive substances, NPS*finder*^®^, novel synthetic opioids, addiction, early warning system, substance misuse

## Abstract

**Background/Objectives:** The rapidly evolving crisis of Novel Synthetic Opioids (NSOs) poses a serious and growing threat for global public health. NSOs include prescription/non-prescription opioids (fentanyl, non-fentanyl analogues), herbal derivatives, and other emerging analogues that are of critical concern due to their high potency, misuse potential, and addiction and intoxication risk. There remains an important gap in real-time, systematic monitoring of NSOs emergence, especially in online communities where these substances appear for the first time. This study aimed to employ the NPS*finder*^®^ automated web-crawling tool to detect, monitor, analyse, and evaluate the evolving NSOs scene. **Methods:** Data were collected during two time-periods, i.e., 2017–2019 and 2023, from selected high traffic psychonaut online platforms to better understand trends in opioids market evolution and adaptability and compare NPS*finder*^®^ findings with other well-known Early Warning Systems (EWSs) databases to assess detection overlap and early identification capacity. **Results:** Within the selected time-periods, a total of 446 NSOs were detected by NPS*finder*^®^; fentanyl analogues (n = 249) were dominant, with a notable rise in non-fentanyl analogues, especially nitazene-like compounds, in 2023. Over 57% of these NSOs were not captured by any of the other EWSs, confirming the tool’s strong capacity to identify early threats. **Conclusions:** Overall, the low overlap across EWS databases underscores the global challenges in comprehensive opioid detection and surveillance. Future studies should integrate web-crawler findings with real-world datasets. It will be vital to combine these efforts with toxicological, mortality, and clinical outcome analyses, especially for emerging research compounds, to inform targeted harm-reduction strategies.

## 1. Introduction

### 1.1. Health Risks from Novel Psychoactive Substances (NPSs)

The use of NPSs represents an impactful and rapidly growing threat to global public health due to their unpredictable potency, toxicity, dependence, and mortality risk [1,2]. These substances act on the central nervous system (CNS), affecting neurotransmitters and receptors whilst causing psychological and physiological alterations [3,4,5,6]. NPS-related serious adverse effects include nausea, vomiting, headache, gastrointestinal and cardiovascular complications, depression, psychosis, and confusion [7,8]. The use of these substances can lead to highly risky behaviours such as unplanned sexual intercourse and unwanted pregnancy. During lactation, NPS use may have toxic effects on neonates, increasing the risk of brain damage especially in situations where synthetic cannabinoids are used [9]. Attempted and completed suicide and other fatalities have been attributed to NPS use [10]. The Psychoactive Substances Act (PSA) 2016 was introduced in the United Kingdom in order to control NPS trafficking, manufacturing, and availability, although this has apparently not been associated with changes in NPS use [11,12]. One way of classifying NPSs is based on a substance’s origin; more specifically, they are categorised into natural, synthetic, and semisynthetic substances. Natural substances are those directly derived from plants or fungi such as khat (*Catha edulis*), kratom (*Mitragyna speciosa*), salvia (*Salvia divinorum*), psilocybin, and natural cannabinoids from *Cannabis sativa* L. and *Cannabis indica* plants [13]. Conversely, synthetic substances are produced in laboratory settings from chemical precursors through various chemical reactions resulting in compounds such as benzylpiperazines [6,14]. Finally, semisynthetic substances are chemical compounds, such as cocaine hydrochloride, which are prepared by using natural precursors and followed by chemical reactions often in laboratory or other settings [2]. NPSs can also be categorised based on psychopharmacological action, leading to six main groups, i.e., cannabinoids, dissociatives, hallucinogens, sedative/hypnotics, stimulants, and opioids [2].

### 1.2. The Emergence of Novel Synthetic Opioids (NSOs)

NSOs are one of the most potent, dangerous, rapidly evolving, and lethal NPSs classes [1,3,15]. They are characterised by structural diversity and are frequently more potent than traditional opioids [16]. Well-known compounds belonging to the NSOs class include fentanyl, nitazenes, brorphine, and etonitazepyne molecules [1,15]. By 2024, the United Nations Office on Drugs and Crime (UNODC) Early Warning Advisory (EWA) listed over 1100 NPSs. In 2023, 527 NPSs were reported worldwide, specifically synthetic cannabinoids (40%), synthetic cathinones (25%), and NSOs (10%) among the dominant groups. In 2022, a total of 749 seizure cases of NSOs were reported across the European Union (EU), with most of them having been identified in Northern European countries [16]. Fentanyl and its analogues remain the most frequently detected and distributed NSOs in the market. Since these substances have become increasingly subject to control and scheduling, other types of NSOs (e.g., nitazene derivatives) have recently emerged [16,17]. In 2025, the UNODC recorded 26 nitazenes across 30 countries internationally, with these substances being characterised by high toxicity risk [18]. The usual life cycles of NSOs are generally short; following detection and scheduling of the index molecule, manufacturers seem to be able to promptly adapt, whilst producing new products to be made available to the market [17]. In practice, this means that once an NSO is identified and controlled, it may stop circulating after a few months but then promptly be replaced by a new synthesised and structurally similar analogue, bypassing legal constraints, allowing illicit manufacturers to stay ahead of regulatory control and toxicological detection [17]. At both the EU and international levels, following the COVID-19 pandemic and the impact of government restrictions, the number of new NSOs appearing decreased in 2021–2022 [16]. However, an increase in NSOs detection and market presence was observed again in 2023 [16]. NSOs are frequently sold online and mixed with other drugs, often without users being aware; indeed, this may significantly increase the risk of overdose, intoxications, and fatalities [16]. NSOs are known for their extremely strong effects even in low doses, hence contributing to a large number of opioid-related deaths worldwide [15,19]. Severe respiratory depression remains a primary cause of NSOs-related morbidities and fatalities. For instance, vulnerable groups such as adolescents and polysubstance users exhibit significantly lower tolerance, while the combination of NSO use with alcohol, gabapentinoids, and benzodiazepines may substantially increase risk even at low NSO doses [20,21]. In Western countries, such as the USA and Canada, emergency departments have indicated rising cases of NSOs-related respiratory depression requiring repeated or high-dose administration of naloxone, especially in youth groups that have often been exposed to ultra-potent fentanyls or nitazenes that may be life-threatening due to the rapid onset of respiratory failure, even in cases of microgram doses [22,23]. Naloxone remains a first-line treatment for emergency NSOs overdose management, often in repeated or high doses [24]. According to evidence, the use of naltrexone varies, especially in Western countries. Injectable extended-release naltrexone is most commonly used among individuals in rehabilitation with more stable socioeconomic status and those in criminal justice programmes, where there is supervised administration supporting adherence [25,26]. In young adults suffering from opioid use disorder, naltrexone is not used often due to the need for a detoxification period before treatment initiation and there are other preferred options such as buprenorphine and methadone [27]. Vulnerable groups suffering from mental health disorders and addiction face a major relapse risk burden with naltrexone administration [28,29]. Overall, naltrexone use is less common, showing a need for more enhanced accessibility, education, and supportive settings, especially for vulnerable groups. NSOs remain a serious challenge for policy and regulatory authorities, healthcare experts, and forensic toxicology providers.

### 1.3. NPS Markets and Availability

Over the past decade, the availability of novel psychoactive substances (NPSs) has increased significantly, with new compounds continuously entering the market. The NPS market is characterised by persistence, adaptability, and integration with traditional illicit drug markets [3]. The incorporation of digital tools and technologies has further transformed this landscape. Online platforms—including social media, dark/deep web markets, and cryptocurrency-enabled marketplaces—have accelerated the global distribution of substances, broadening their availability, particularly among younger consumers [30]. The internet and social media play a pivotal role in the rising popularity, sale, and marketing of NPSs at the global level [31], with the 18–24 years age-group comprising the majority of users active on these platforms [32,33]. This digital dimension has introduced significant regulatory challenges, requiring more innovative and advanced monitoring tools and control strategies [11,34]. European studies show that there has been a significant increase in the number of young adults using NPSs, with synthetic cannabinoids and NSOs being among the most commonly reported substances [16,35]. Similarly to the EU, digital markets and social media play a crucial role in the dissemination of NSOs and synthetic cannabinoids among young adults in the USA and Canada, where there has been a substantial rise in fentanyl analogues and nitazene derivatives with record numbers of opioid overdose-related deaths [21,23]. The North American landscape is characterised by a higher prevalence of illicitly manufactured fentanyl, carfentanil, and tablets mimicking prescription medications [22]. There is a mirroring of EU observations referring to increased NPSs experimentation among young adults with a more severe overdose burden [22,23]. Many NPSs are marketed in attractive, colourful packaging with catchy names, making them especially appealing to vulnerable groups such as adolescents, youth, and individuals with mental health disorders who are more susceptible to substances experimentation, risk-taking behaviours, and dependence [3,36,37]. Drug fora and blogs—often referred to as ‘e-psychonaut’ platforms—have gained considerable popularity, attracting large audiences of online users [34,38]. On these web pages, individuals who use NPSs to explore or induce altered states of consciousness openly share their experiences. However, these platforms may pose a significant risk to users’ health and safety, as the spread of misinformation especially among younger or inexperienced users may cause an underestimation of the serious health risks associated with NPS use [39,40,41,42,43,44,45,46]. Many recreational users assume that NPSs are safe simply because they are legal or not yet controlled under current drug legislation [4,11,47]. However, NPS use has been strongly associated with polysubstance use, including alcohol, nicotine, and illicit substances [48]. Polysubstance abuse has been linked to NSO-related overdose fatalities, with isotonitazene and related substances becoming more frequently implicated [49].

### 1.4. Aims of the Study

The aim of this research study was to identify and predict the emerging substances, usage patterns, and current market availability of NSOs. Focusing on the post COVID-19 pandemic period, the study aimed also to investigate potential shifts in the NSOs scenario. The study also used information from the existing international early warning systems (EWSs), to assess if the NPS*finder*^®^ tool could act as a useful mechanism for identifying emerging trends, thereby supporting timely policy responses.

## 2. Results

**Figure 1:**
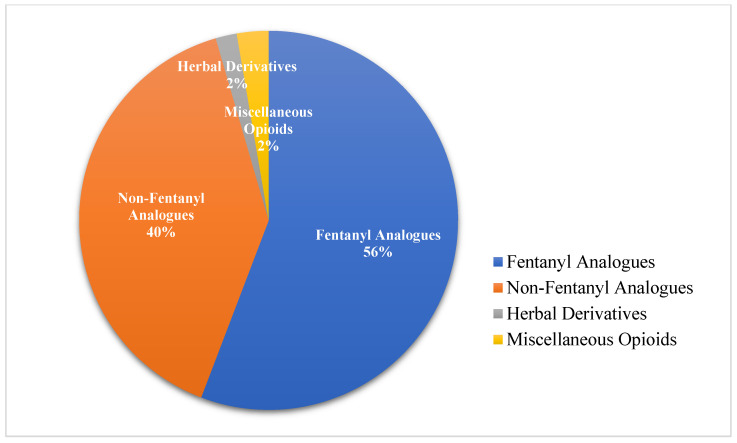
Classification of identified NSOs (2017–2019 and 2023).

A total of 446 unique NSOs were identified via NPS*finder*^®^ across both web-crawling periods (2017–2019 and 2023) (see Supplementary Tables S1, S2A,B, S2(Ca–Cc) and S3A,B). These substances were detected through systematic monitoring of high traffic psychonaut platforms. After expert manual review and categorization, these NSOs were classified into four main categories (See Figure 1):

- Fentanyl analogues (n = 249; 56%);
- Non-fentanyl analogues (e.g., nitazenes, mu/kappa agonists, prescription opioids, precursors) (n = 177; 39%);
- Herbal derivatives (n = 8; 2%);
- Miscellaneous opioids (n = 12; 3%).

Further details on the classification breakdown of the substances identified during the two crawling sessions (2017–2019 and 2023) are given in Table 1.

Table 2 presents the range of representative substances from the main identified NSOs classes, with their key characteristics.

**Figure 2:**
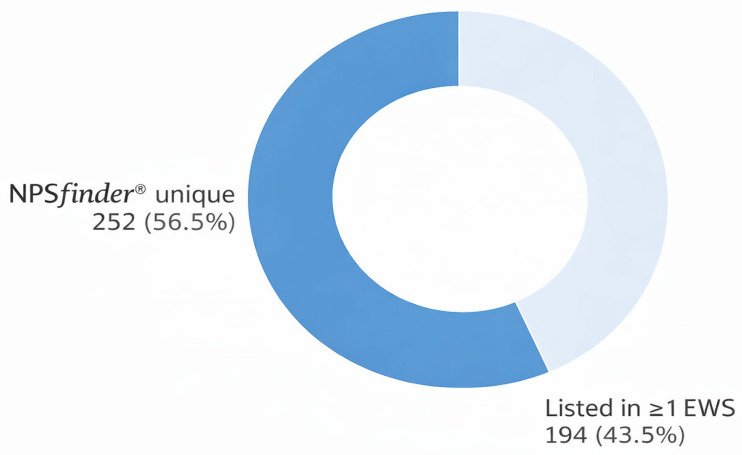
NSOs unique to NPS*finder*^®^ compared to the other EWSs databases.

Figure 2 shows that across the 446 NSOs detected and screened across international EWSs of drug monitoring, 252 (57%) were identified uniquely by NPS*finder*^®^, while 194 (44%) were listed in at least one of the other EWS databases in this study (*UNODC EWA, CFSRE/NPS Discovery, INCB Yellow/Green List).* NPS*finder*^®^ detected more than half of all the NSOs recorded in this study, showing its strong capacity in early detection, broad coverage, and early insights into emerging opioid trends before formal inclusion in other monitoring EWSs.

Each NSO was systematically searched across the above-listed EWSs. Substances not listed in any of these databases at the time of data extraction were deemed unique to NPS*finder*^®^. Besides the NPS*finder*^®^ tool, among the EWSs examined, the INCB Yellow List contained the highest number of detected NSOs followed by UNODC EWA.

**Figure 3:**
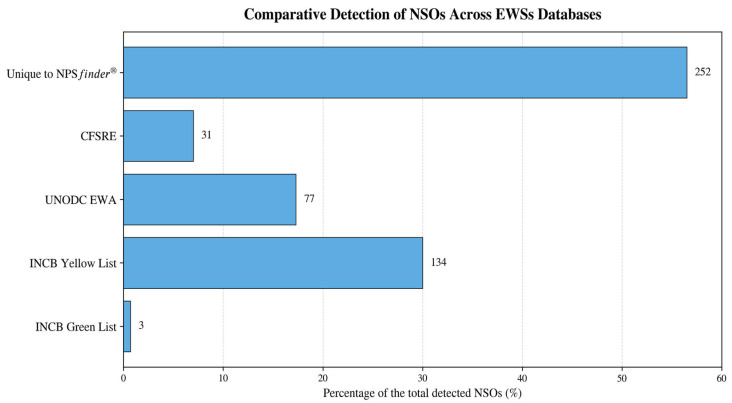
Performance of the NPS*finder*^®^ tool in detection of NSOs compared to other EWS databases with NSOs uniquely identified for each list.

Specifically, 134 NSOs (30% of the total 446; 95% CI: 25.98–34.46%) appeared on the INCB Yellow List. The UNODC EWA included 77 substances (17%; 95% CI: 13.90–20.85%), CFSRE/NPS Discovery had 31 (7%; 95% CI: 4.71–9.42%), and the INCB Green List had 3 entries (1%; 95% CI: 0.23–1.94%), mostly controlled medical substances (Figure 3). In total, 252 substances (57%; 95% CI: 52.47–61.66%) were identified exclusively by NPS*finder*^®^, emphasising this tool’s predictive strength in detecting NSOs prior to their formal listing by international regulatory agencies.

**Figure 4:**
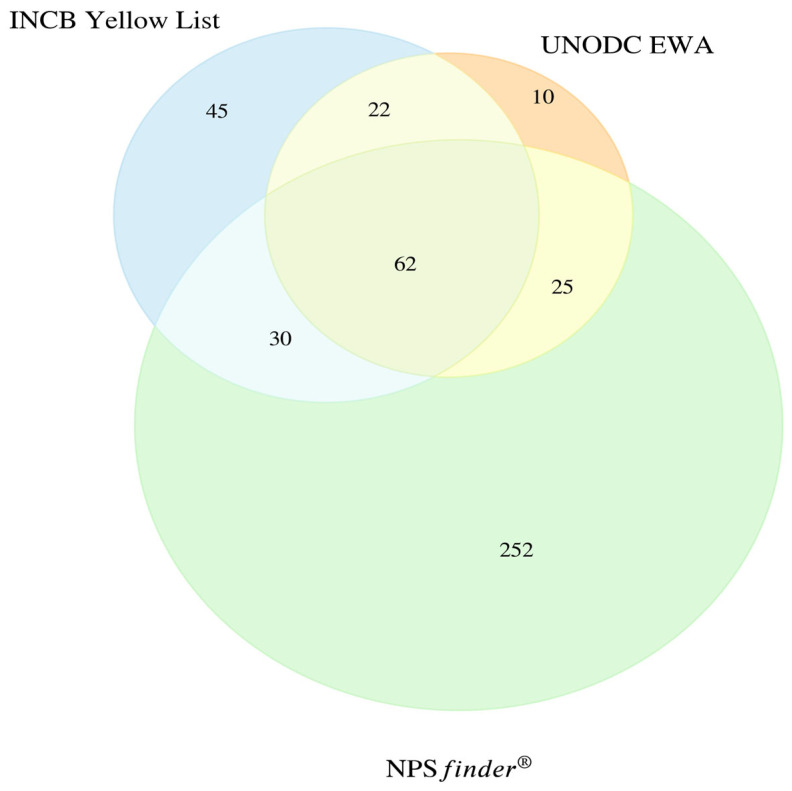
Overlap of NSOs across international EWSs (n = 446). CFSRE/NPS Discovery and INCB Green List were not included in the diagram due to the limitations of the Venn diagram and the smaller number of substances and minor overlaps detected.

The Venn diagram in Figure 4 shows the overlap of detections between the three international EWSs with the highest number of NSOs detected, INCB Yellow List, UNODC EWA, and NPS*finder*^®^. Only 62 substances (14%) were detected by all three EWSs. NPS*finder*^®^ showed the greatest detection capacity by identifying an overall total of 369 NSOs with 252 unique detections. The INCB Yellow List followed with 159 NSOs, while UNODC EWA recorded 119 substances in total.

- 45 substances detected only by INCB Yellow List;
- 30 shared between INCB Yellow List and NPS*finder*^®^ tool;
- 25 shared between UNODC EWA and NPS*finder*^®^ tool;
- 22 detected by INCB Yellow List and UNODC EWA, but not NPS*finder*^®^ tool;
- 10 exclusive to UNODC EWA;
- 252 unique to NPS*finder*^®^.

The 252 substances unique to NPS*finder*^®^ were predominantly fentanyl analogues (176), followed by non-fentanyl analogues (45; including nitazene-like compounds, mu/kappa agonists, and miscellaneous opioids), 16 non-fentanyl prescription opioids, 5 herbal derivatives, and 10 miscellaneous opioids. CFSRE documented 19 fentanyl analogues and 12 non-fentanyl analogues; UNODC listed 57 fentanyl analogues, 18 non-fentanyl analogues, and 2 herbals; the INCB Yellow List documented 27 fentanyl analogues, 77 non-fentanyl analogues (including 44 non-fentanyl prescription opioids, 2 miscellaneous opioids, and 1 herbal); and finally the INCB Green List included 2 non-fentanyl analogues and 1 non-fentanyl prescription opioid (Figure 4).

**Figure 5:**
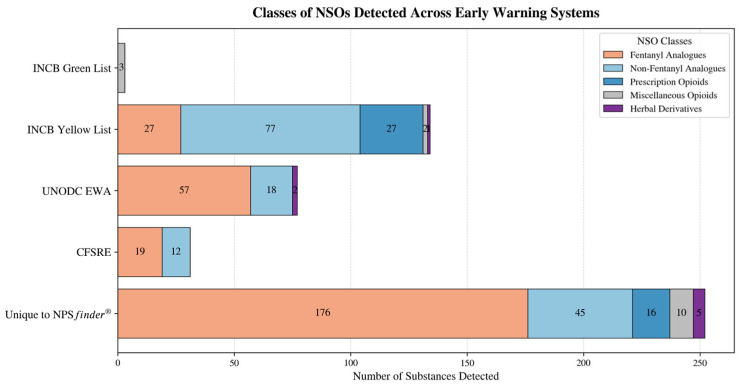
NSOs classes and number of substances detected across the different EWSs.

The stacked bar chart in Figure 5 displays a breakdown of NSOs by class across each EWS. Of the 252 unique NSOs identified by NPS*finder*^®^,

- 176 were fentanyl analogues;
- 45 were non-fentanyl analogues (including nitazene-like, mu/kappa agonists);
- 16 were prescription opioids;
- 10 were miscellaneous opioids;
- 5 herbal derivatives.

Each EWS showed a distinct pattern on NSOs detection:

- INCB Yellow List identified mainly non-fentanyl opioids and prescription substances;
- UNODC EWA enlisted mainly fentanyl analogues;
- CFSRE contained mostly fentanyls and nitazenes;
- INCB Green List included miscellaneous opioids/precursors.

**Figure 6:**
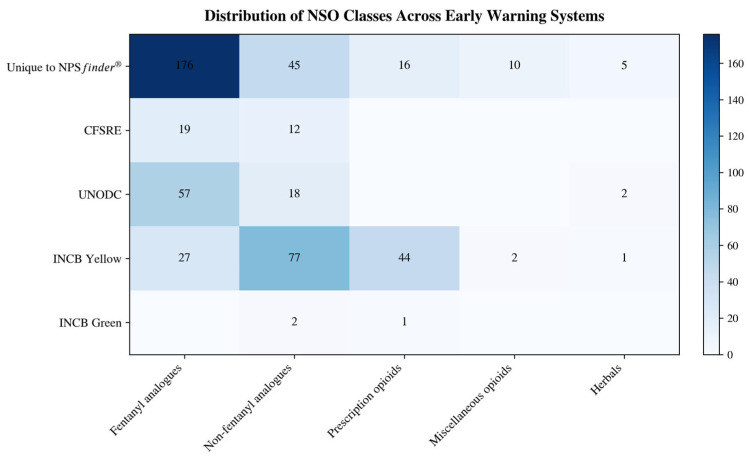
Heatmap of NSO classes detected across early warning systems (n = 446).

Figure 6 presents a ‘heatmap’ which further illustrates the above-described variations across substance classes and the five EWSs investigated. NPS*finder*^®^ uniquely detected the highest number of fentanyl analogues (n = 176) and non-fentanyl analogues (n = 45). INCB Yellow List listed regulated non-fentanyl compounds (n = 77) and prescription opioids. CFSRE/NPS Discovery primarily confirmed fentanyls (n = 19) and non-fentanyls (n = 12).

## 3. Discussion

### 3.1. Detection of NSOs: NPSfinder^®^ vs. EWSs

This current study uniquely provides an updated insight into novel opioids emerging in the post- COVID-19 pandemic era, reaffirming the predictive utility of NPS*finder*^®^ compared to other global EWSs [39]. Fentanyl analogues remained the most frequently detected class, and also the class with the highest number of substances uniquely identified (176) by NPS*finder*^®^ that other EWSs failed to detect. This underscores the early online emergence and widespread availability of fentanyl analogues within psychonaut communities before formal identification and reporting. Non-fentanyl analogues, particularly nitazene-like compounds and mu/kappa receptor agonists, proved more challenging to detect consistently across systems. While 77 of these were captured by the INCB Yellow List, 45 remained unique to NPS*finder*^®^. For non-fentanyl prescription opioids, most were listed in the INCB Yellow List, yet over one-third (36%) appeared only in the NPS*finder*^®^ database and not in other EWSs. Miscellaneous opioids (e.g., dextromorphan and research-chemical morphine analogues) were rarely identified by EWSs and were predominantly recorded via NPS*finder*^®^, reflecting a critical surveillance gap. Herbals such as *Mitragyna speciosa* (kratom) and *Salvia divinorum* likewise showed poor detection in EWSs, with over 60% uniquely recorded by NPS*finder*^®^. Only 11 NSOs were jointly detected by NPS*finder*^®^, the INCB Yellow List, and UNODC. Although each EWS identified some distinct NSOs, NPS*finder*^®^ demonstrated the strongest performance in early detection. Over time, NPS*finder*^®^ has maintained and even expanded its levels of usefulness by identifying new opioids before formal regulatory listing.

### 3.2. Public Health Threats Posed by NSOs

NSOs occupy an increasing share of the NPS landscape. In Europe, fentanyl derivatives are consistently flagged up as among the most dangerous NPS classes [16]. The rising use of NSOs points to user demand for highly potent synthetic alternatives, amplifying risks of unpredictable toxicity, dependence, and overdose fatalities [16,19]. Since NSOs exert potent effects at low doses, even small dosing errors can be lethal [15,19]. Their illicit production and distribution contribute significantly to global opioid-related mortality [15]. Fentanyl analogues remain a leading cause of opioid mortality globally [16,51,52,53,54,55]. In recent years, strict regulation of fentanyl analogues has coincided with a rise in nitazene derivatives, mirroring the present findings of newly emerging nitazene-like compounds [16,17]. Newly emerging fentanyl analogues were also detected by this study in 2023, indicating continued market persistence despite regulatory constraints, underscoring the need for sustained monitoring and adaptive policy measures. The typical life-cycle for NSOs is relatively short due to detection pressures, but manufacturers appear adept at rapidly diversifying compound portfolios and maintaining product turnover [17]. NSOs continue to challenge policy advisors, regulators, medical personnel, and forensic toxicology providers.

### 3.3. Role of EWSs in NSO Surveillance

Post COVID-19 pandemic trends suggest an escalation in NSO-related risks, underscoring the need for agile and continuous surveillance systems [16,56,57]. Comparative analyses across EWSs (UNODC, CFSRE, INCB) can illuminate trends in NSO prevalence, emerging classes, and risk profiles, thereby guiding prompt responses. The ability of NPS*finder*^®^ to detect and classify novel substances in real time makes it a valuable addition to existing surveillance ecosystems [58,59]. Its workflow supports the systematic scraping, filtering, detection, extraction, and classification of emerging compounds from psychonaut fora, marketplaces, and other digital sources [41,58,60,61]. Web-based surveillance methods tend to outperform traditional forensic, toxicologic, or clinical case reporting in terms of timeliness [62]. Web crawlers like NPS*finder*^®^ have demonstrated the capacity to flag up emerging NPSs months before they appear in toxicological databases, highlighting the utility of integrating digital tools with public health efforts [58,59,63]. These early warning data are invaluable for researchers, clinicians, and policymakers. During global crises, such as the COVID-19 pandemic, such systems have proven especially beneficial. For example, a French addictovigilance tool linked pharmacy reports, clinical data, and emergency admissions to monitor intoxications in real time [64]. Different monitoring platforms adopt various methods, sources, and techniques, which can lead to disparities in detection speed and coverage [63,65]. Real-time digital surveillance tools like NPS*finder*^®^ will increasingly play a pivotal role in shaping public health strategy, regulatory decision-making, and harm-reduction initiatives. In addition to EWSs, epidemiological methods such as wastewater-based analysis have demonstrated promise in capturing population-level trends in NPS availability and consumption [66,67,68].

### 3.4. NSO Market Adaptability and the Role of Digital Tools

The agility of NPS markets enables the continuous introduction of new substances despite regulatory controls. Even minimal structural modifications can significantly alter potency or toxicity, complicating regulation [63,69,70,71]. Given the rising reliance on online platforms for drug procurement, continuing to develop systematic digital monitoring of NSO markets is crucial. Digital harm-reduction campaigns have shown effectiveness, especially when tailored to vulnerable populations, by delivering evidence-based information [72,73,74]. Strategic partnerships among experts operating in online drug-information communities, social media platforms, and digital health organisations could facilitate the rapid dissemination of harm-reduction messaging [72,75]. In-depth communication and collaboration between public health regulatory agencies, social media platforms, blog fora, online marketplace moderators, and cybersecurity experts could also support the prompt detection, control, and removal of harmful content, identification of suspicious sales, and targeted online harm-reduction with evidence-based information. Conducting prevention programmes and advancing digital tools literacy may reduce misinformation risk. Special attention must be paid to vulnerable groups, e.g., adolescents, young adults, and individuals with mental health or substance-use disorders, who are more susceptible to peer influence and normalisation of illicit substances use and sourcing and access via online platforms increasing the harm risk [31,44,76,77,78]. The popularity of NSOs is driven not only by their powerful psychoactive effects but also by the perception that they are ‘legal’ alternatives to illicit drugs. Their uncontrolled status can lead users to believe that they are ‘safer’, and they often evade routine drug screening detection [11,79,80,81]. One could argue that online NSO discussions often predict real-world market developments, with mentions in fora, blogs, and darknet marketplaces preceding clinical or forensic detection. Hence, digital surveillance tools like NPS*finder*^®^ offer valuable early warning capacity, enabling the proactive monitoring of users’ evolving behaviours and facilitating timely interventions. It is crucial to strengthen the utilisation of digital surveillance tools and develop rapid-alert systems to keep toxicologists, clinicians, and researchers informed about new high-risk opioids trending in the open and dark web for effective application of early response measures and risk mitigation.

### 3.5. Implications for Clinical Pharmacology, Toxicology and Policy

The NSOs detected by this study show pharmacodynamic diversity according to their chemical structure. Most fentanyl analogues are characterised by an anilidopiperidine backbone, where substitutions on the N-phenyl ring or the acyl side chain alter μ-opioid receptor affinity, efficacy, and lipophilicity [82]. Ultra-potent analogues, such as carfentanil and lofentanil, are associated with rapid onset and severe respiratory depression at extremely low doses due to lipophilic structures and rapid blood–brain penetration [20]. The nitazenes identified in the findings are characterised by a benzimidazole core with μ-receptor affinity, where slight modifications to the core may change and increase potency as well as efficacy [83]. Nitazene derivatives are associated with severe side-effects, respiratory depression, and high toxicity. The dataset compiled for this study also includes κ-opioid receptor agonists and miscellaneous opioids with mixed and unknown exact mechanisms of action. K-selective receptor compounds such as salvinorin A and analogues produce dissociative and psychotomimetic effects [84]. Mixed-mechanism and miscellaneous opioids act on monoaminergic systems through serotonin and noradrenaline reuptake inhibition, with a risk of serotonin syndrome and severe toxicity [85]. Plant-derived and semisynthetic opioids, such as kratom and granulated opium, are characterised by further pharmacological and toxicological complexities. Mitragynine and indole alkaloid analogues are characterised for partial μ-agonism with mixed stimulant opioid effects. Metabolites, intermediates, and fentanyl precursors are pharmacologically active or partially active and act as markers of illicit synthesis routes. The diversity and heterogeneity in the chemical structures and pharmacological profiles of the identified NSOs show that even modest substitutions on core opioids can increase severe effects, respiratory depression, modify metabolic pathways, and make it difficult to be detected in toxicology assessments. NPSfinder^®^ can and does inform and support regulatory decision-making, pharmacological and toxicological preparedness, and clinical practice by identifying emerging compounds even years before they appear in official toxicology reports. The early recognition of trends supports pharmacology research and informs clinicians and policymakers to initiate scheduling efforts and targeted harm-reduction strategies. The findings presented here have direct implications for clinical practice and policy. The early identification of structures associated with high potency can support analogue scheduling and legal frameworks in the early stages of breakouts [86]. For harm-reduction strategies, the analysis of demographic characteristics, digital market trends, and patterns of use highlights the need for targeted prevention initiatives, especially in vulnerable populations, including youth [22]. These findings support the integration of NSO surveillance tools into routine emerging substances monitoring, training of clinicians, and public health promotion.

### 3.6. Study Strengths and Limitations

#### 3.6.1. Strengths

This study is among the first globally to systematically compare NPS identification across large international EWS databases using a web-based detection tool. It highlights the strength of NPS*finder*^®^, demonstrating its capacity to detect emerging NSOs, at times well before formal listing in systems like the UNODC EWA and INCB Yellow Scheme. It underscores NPS*finder*^®^’s utility as an innovative resource for addiction researchers, EWS authorities, and policymakers in responding to the global NPS challenge. The tool supports the effective classification of complex NSO entries, including compounds not yet captured by other surveillance systems. The principal strength lies in leveraging NPS*finder*^®^ to detect online market availability at the earliest possible point, which is essential for risk assessment and policy planning.

#### 3.6.2. Limitations

NPS*finder*^®^ relies solely on online data sources and, therefore, may suffer from selection biases such as language restrictions, platform coverage limits, and dependence on user-reported content. For instance, the NPS*finder*^®^ algorithm may detect predefined keywords and chemical nomenclatures, influencing which NSOs are capable of being identified based on this approach. There is a potential that the tool may generate false positives and miss some detections due to the keyword-dependent operation. There is also a difficulty in detecting unknown abbreviations or ambiguous terms, novel street name terms, confusing promotional advertisements, and emerging substances that may lack available descriptive information. It might also not reflect offline markets or NPS usage outside of digital spaces. The study does not incorporate pharmacological toxicity profiles or clinical outcome data for emerging NSOs, limiting the ability to generalise risk assessment beyond detection. Data collection was confined to open-surface web resources and a selected set of psychonaut websites. Although these sites were chosen based on high traffic and user engagement, relevant information from other platforms may have been missed. The tool currently does not analyse dark web markets, which host a significant number of opioid trafficking, and despite the multilanguage ability, it may not detect all the emerging substances discussed in less frequently scanned languages or regional domains. The searching algorithm of the tool detects the report of a substance name, but cannot automatically validate its pharmacological profile, structure, or real-world use patterns without confirming with clinical or toxicology forensic data. Full reproducibility is constrained due to this advanced and complex web-crawling methodology. To attain a more comprehensive perspective on NSO surveillance, it is essential to cross-validate findings with alternative tools, forensic data, clinical records, and epidemiological methods.

Future studies should expand the monitoring and trend analysis of NSOs via longitudinal designs that integrate web-crawler findings with real-world datasets. It will be vital to combine these efforts with toxicological, mortality, and clinical outcome analyses, especially for emerging research compounds and substances flagged via social media, to inform targeted harm-reduction strategies for adolescents and young adults. Enhanced collaboration, data-sharing, and dissemination among regulatory EWSs and digital surveillance platforms can support more agile and evidence-based policy responses to the evolving NSO threat.

## 4. Materials and Methods

### 4.1. Identification of Substances with the Help of the NPSfinder^®^ Web-Crawling Tool

To monitor the emergence of NSOs online, two web-crawling sessions were conducted using NPS*finder*^®^, an advanced 24/7 web-crawling software (2023 version of the NPSfinder software) developed by Damicom S.R.L. (Rome, Italy). The tool continuously scans the open/surface web and automatically collects data on NPSs from high traffic psychonaut and drug-related fora. It supports multiple languages, including English, Spanish, German, Italian, French, Dutch, Russian, Turkish, and Swedish. Extracted data include chemical and street names, molecular structures, and user-reported psychoactive effects. All information is securely stored in a password-protected database on a secure server. The first web-crawling session was conducted from November 2017 to May 2019, and its dataset was previously analysed to evaluate NPS*finder*^®^’s predictive capacity in comparison to international EWS entries [39]. The second session, whose data are presented first here, occurred between January and September 2023, focusing on post COVID-19 pandemic NSO trends. Both datasets were used to examine the evolution of NSO market dynamics and to reassess NPS*finder*^®^’s usefulness in better understanding current and future drug scenarios. All data were manually reviewed by the research team, systematically filtered, de-duplicated, and analysed. Each substance was evaluated for psychoactive effects and classified based on its mechanism of action and chemical characteristics using the literature and chemical databases. Substances were then added to a continuously expanding database of emerging NPSs. The unique capabilities of NPS*finder*^®^ allow for the real-time surveillance of drug trends discussed online, often identifying substances before they are formally detected by regulatory or toxicological monitoring systems. This function is critical in supporting expert and policymaker responses to the rapidly evolving NPS landscape. Further details on the design and functionality of NPS*finder*^®^ are available in previous publications by our research team [41,45,58,60].

### 4.2. Data Collection and Screening

**Figure A1:**
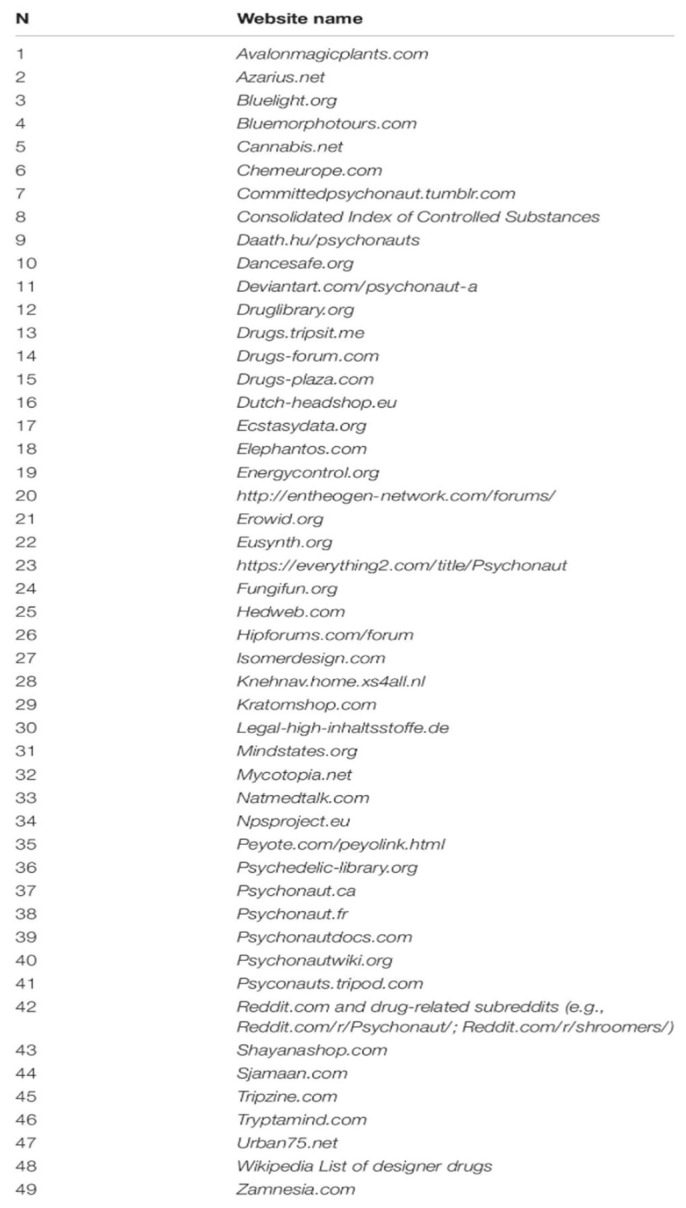
List of websites in open web surface that were selected to be searched by the NPS*finder*^®^ web crawler tool (2017–2019, first web-crawling session).

In the 2017–2019 web-crawling session, NPS*finder*^®^ collected information on 5922 substances, with 4204 confirmed as unique NPSs; the remainder were duplicates or false positives. The monitored platforms included popular drug fora, blogs, and other websites frequented by psychoactive substance users (Appendix A; Figure A1). Of these, 426 substances were identified as novel opioids. In the 2023 session, 370 entries were collected from high traffic platforms (Appendix B; Table A1). After removing one duplicate, 369 unique substances remained. Of these, 159 were newly identified NPSs, including 23 NSOs, which were added to the updated NPS*finder*^®^ database.

Data exported by Damicom’s IT team were processed in Excel. Initial screening for novelty, psychoactive properties, and abuse potential was conducted by the first author (E.D.). Substances were then further assessed by a multidisciplinary team of pharmacology and psychiatry experts (F.S., A.V., D.A., L.L.). Classification was based on chemical structure, pharmacological action, and known effects, using resources such as PubChem, IUPAC nomenclature, and the published literature (https://pubchem.ncbi.nlm.nih.gov; https://iupac.org/what-we-do/nomenclature/) (accessed on 15 May 2025). Ambiguities or challenging classifications were resolved collaboratively through team discussions to ensure scientific accuracy and reliability.

### 4.3. Comparison with International EWS Databases

To evaluate the power of NPS*finder*^®^ in detecting emerging NSOs, a comparative analysis was conducted between the tool’s datasets (2017–2019 and 2023) and listings from major international early warning systems, as of June 2025. The following EWS databases were consulted:

- United Nations Office on Drugs and Crime (UNODC) Early Warning Advisory (EWA) on New Psychoactive Substances (NPS). https://www.unodc.org;
- International Narcotics Control Board (INCB) Yellow List—List of Narcotic Drugs under International Control (1961 Single Convention). https://www.incb.org/incb/en/narcotic-drugs/Yellowlist/yellow-list.html (accessed on 15 May 2025);
- International Narcotics Control Board (INCB) Green List—List of Psychotropic Substances under International Control (1971 Convention). https://www.incb.org/incb/en/psychotropics/green-list.html (accessed on 15 May 2025);
- Center for Forensic Science Research & Education (CFSRE) “NPS Discovery” Database/Early-Warning System. https://www.cfsre.org.

Each NSO detected during web crawling was searched for chemical properties, mechanisms of action, and whether it had been officially listed by any EWS between its online detection (2017–2019 or 2023) and the 2025 follow-up. This five-year follow-up was essential to assess the long-term predictive validity of NPS*finder*^®^. The systematic comparison aimed at revealing how many substances first detected in online psychonaut fora were later formally listed in regulatory EWS databases.

### 4.4. Ethics and Data Security

All data extracted were publicly available. All data handling procedures complied with institutional ethical standards and General Data Protection Regulation (GDPR) requirements. The NPS*finder*^®^ database remains securely hosted and is accessible only to authorised researchers.

## 5. Conclusions

This study delivers an up-to-date detection and systematic classification of 446 NSOs identified via two web-crawling sessions (2017–2019 and 2023) using NPS*finder*^®^. Over 57% of these NSOs were not captured by any of the other EWSs, confirming the tool’s strong capacity in early threat identification. Fentanyl analogues were dominant, with a notable rise in non-fentanyl analogues, especially nitazene-like compounds, in 2023. Overall, the low overlap across EWS databases underscores the global challenges in comprehensive opioid detection and surveillance.

## Figures and Tables

**Table 1:** Breakdown of classification of NSOs detected in the two crawling sessions (2017–2019 and 2023).

Classification	Count	% of Total	95% CI (Lower–Upper)
Fentanyl Analogues (2017–2019)	237	53	48.50–57.72
Fentanyl Analogues (2023)	12	3	1.55–4.64
Non-Fentanyl Analogues—Prescription (ATC/DDD *) (2017–2019)	44	10	7.43–12.99
Non-Fentanyl Analogues (2023)	11	2	1.38–4.36
Non-Fentanyl μ (mu)—Agonists (2017–2019)	113	25	21.52–29.57
Non-Fentanyl κ (kappa)—Agonists (2017–2019)	9	2	1.07–3.79
Miscellaneous Opioids (2017–2019)	12	3	1.55–4.64
Herbal Derivatives (2017–2019)	8	2	0.91–3.50

* ATC = Anatomical Therapeutic Chemical classification; DDD = defined daily dose [50].

**Table 2:** Representative substances of the main identified opioid classes.

Category	Representative Substances	Characteristics
Fentanyl Analogues	Acryloylfentanyl, Alfentanil, Carfentanil, Fluorofentanyl, Remifentanil	Ultra-potent; High overdose risk
Non-Fentanyl (Nitazene-like)	Dipyanone, Isotonitazene, Metonitazene, Protonitazene	Some ultra-potent; Research compounds—precursors
Non-Fentanyl(Prescription Opioids)	Codeine, Hydromorphone, Methadone, Morphine, Oxycodone	Widely prescribed; Clinically used with high misuse risk; Listed in ATC/DDD Index
Non-Fentanyl (Miscellaneous Opioids)	Salvinorin A, U-48800, W-15	Unclear activity of receptors; Users’ suicide-related reports (W-15)
Herbals	Kratom, Salvia divinorum	Ethnobotanicals; Variable regulations

## Data Availability

The original contributions presented in this study are included in the article/Supplementary Materials. Further inquiries can be directed to the corresponding author.

## Supplementary Materials

The following supporting information can be downloaded at https://www.mdpi.com/article/10.3390/ph19010017/s1, Table S1—NPS*finder*^®^ fentanyl analogues; IUPAC names and comparisons between the different databases. NPS*finder*^®^ crawling in 2018 Table 1 (Arillotta et al., 2020 [39], Table 1); Table S2A—NPS*finder*^®^ ATC/DDD non fentanyl analogues (ATC/DDD opioids) and comparison between the different databases. NPS*finder*^®^ crawling in 2018 (Arillotta et al., 2020 [39], Table 2A). Table S2B—NPS*finder*^®^ plants and derivatives and comparison between the different databases. NPS*finder*^®^ crawling in 2018. (Arillotta et al., 2020 [39], Table 2B); Table S2Ca—NPS*finder*^®^ non fentanyl μ(mu)-opioid receptor agonists including those with partial/weak and potent/very potent action (miscellaneous opioids); comparison between the different databases. NPS*finder*^®^ crawling in 2018. (updated table from Arillotta et al., 2020 [39], Table 2C) Table S2Cb—NPS*finder*^®^ non fentanyl κ(kappa)-opioid receptor agonists including those with partial/weak and potent action (miscellaneous opioids); comparison between the different databases. NPS*finder*^®^ crawling in 2018. (updated table from Arillotta et al., 2020 [39], Table 2C); Table S2Cc—NPS*finder*^®^: miscellaneous opioids; e.g., non-fentanyl precursors; intermediates; inactive metabolites; comparison between the different databases. NPS*finder*^®^ crawling in 2018. (updated table from Arillotta et al., 2020 [39], Table 2C); Table S3A—NPS*finder*^®^: fentanyl analogues and precursor-like compounds; comparison between the different databases. NPS*finder*^®^ crawling in 2023; Table S3B—NPS*finder*^®^: non fentanyl analogues (e.g., nitazene-like molecules, kratom-related compounds and other miscellaneous opioids); comparison between the different databases. NPS*finder*^®^ crawling in 2023.

## Abbreviations

The following abbreviations are used in this manuscript:

NSOs	Novel Synthetic Opioids
EWSs	Early Warning Systems
NPSs	Novel Psychoactive Substances
CNS	Central Nervous System
PSA	Psychoactive Substances Act
EWA	Early Warning Advisory
EU	European Union
COVID-19	Coronavirus Disease 19
UNODC	United Nations Office on Drugs and Crime
INCB	International Narcotics Control Board
CFSRE	Center for Forensic Science Research & Education
GDPR	General Data Protection Regulation

## Appendix B

**Table A1:** List of websites in open web surface that were selected to be searched by the NPS*finder*^®^ web crawler tool (2023, second web-crawling session).

N	Website Name	URLs
1	Drugsdata	https://www.drugsdata.org/
2	Chemicalfrog	https://chemicalfrog.com/
