# Supplementary material for: Novel Synthetic Opioids (NSOs) and Their Evolving Crisis: Utilising NPSfinder® as a Real-Time Predictive Tool
Source: Pharmaceuticals (Basel). 2025 Dec 21;19(1):17. doi: 10.3390/ph19010017 (PMC12845053; doi:10.3390/ph19010017)
Supplement: Supplementary file 1 [file pharmaceuticals-19-00017-s001.zip › pharmaceuticals-4017890-supplementary.pdf]

Supplementary tables: Details on NSOs detected (nomenclature, chemical structure, EWSs detection)

***N.A: Refers to Not Available information.**

N	Molecule denomination in NPS*finder*®	Other Names	Chemical Name (IUPAC)	Chemical Structure	Molecular Formula	UNODC EWA on NPS (May 2025)	CFSRE (NPS Discovery) (December 2024)	INCB Yellow list (July 2024)	INCB Green list (January 2025)	Unique to NPS*finder*® database
**1**	(Iso)Butyryl-F-Fentanyl N-Benzyl Analogue	N-Benzyl-p-fluoro-isobutyrylfentanyl	1-(1-benzylpiperidin-4-yl)-1-(4-fluorophenyl)-3-methylbutan-2-one	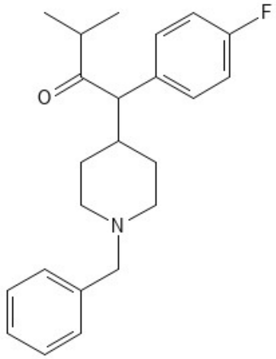	$C_{23}H_{28}FNO$	**Y**	**N**	**N**	**N**	**N**

**2**	2-Fluorofentanyl	ortho-fluorofentanyl	N-[2-Fluoro-1-(2-phenylethyl)piperidin-4-yl]-N-phenylpropanamide	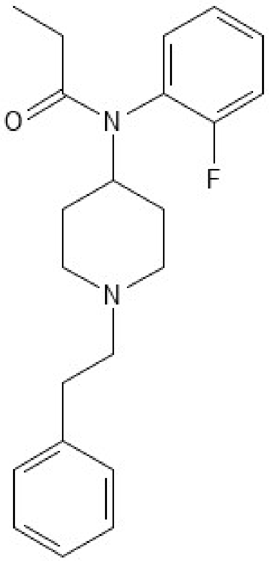	C22H27FN2O	**Y**	**Y**	**Y**	**N**	**N**
**3**	2-Isopropylfuranylfentanyl	o-Isopropyl-furanylfentanyl	N-[1-(2-Phenylethyl)piperidin-4-yl]-N-[2-(propan-2-yl)phenyl]furan-2-carboxamide	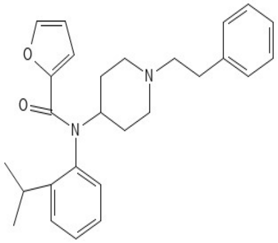	$C_{27}H_{32}N_2O_2$	**Y**	**N**	**N**	**N**	**N**

**4**	2-Methylfentanyl	Ortho-methylfentanyl	*N*-(2-methylphenyl)-*N*-[1-(2-phenylethyl)piperidin-4-yl]propanamide	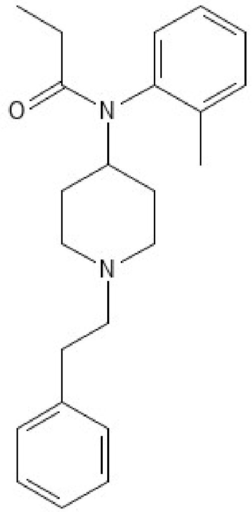	$C_{23}H_{30}N_2O$	**Y**	**Y**	**N**	**N**	**N**
**5**	3-Allylfentanyl		*N*-phenyl-*N*-[(3*S*,4*R*)-1-(2-phenylethyl)-3-prop-2-enylpiperidin-4-yl]propanamide	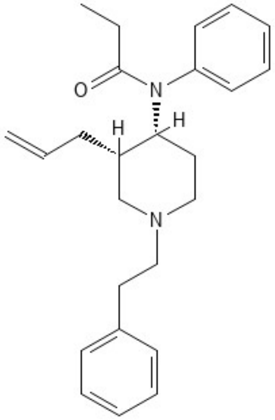	$C_{25}H_{32}N_2O$	**N**	**N**	**N**	**N**	**Y**

**6**	3-Fluorofentanyl	NFEPP	N-[3-Fluoro-1-(2-phenylethyl)piperidin-4-yl]-N-phenylpropanamide	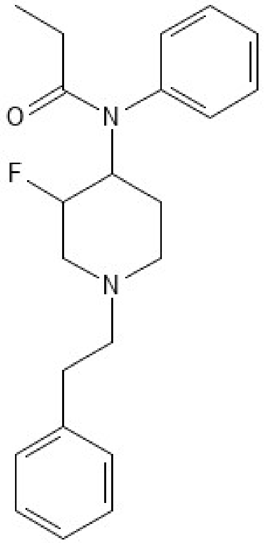	$C_{22}H_{27}FN_2O$	**Y**	**N**	**N**	**N**	**N**

**7**	3-Methyl-Butyrylfentanyl	3-MBF	N-[3-Methyl-1-(2-phenylethyl)piperidin-4-yl]-N-phenylbutanamide	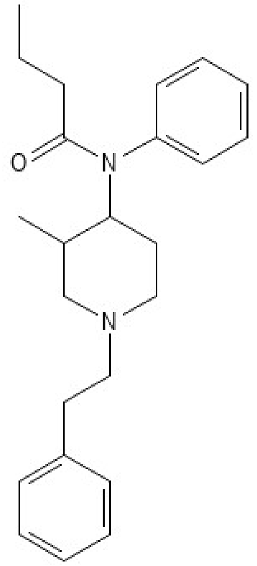	$C_{24}H_{32}N_2O$	**N**	**N**	**N**	**N**	**Y**
**8**	3-Methylcrotonylfentanyl		N.A	N.A	N.A	**Y**	**N**	**N**	**N**	**N**

**9**	3-Methylfentanyl (3-Mf)	Mefentanyl; 3-MF	N-[3-Methyl-1-(2-phenylethyl)piperidin-4-yl]-N-phenylpropanamide	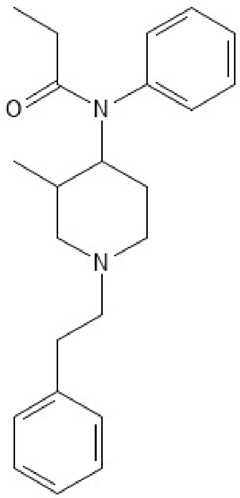	$C_{23}H_{30}N_2O$	**N**	**N**	**Y**	**N**	**N**
**10**	3-Methylthiofentanyl		N-{3-methyl-1-[2-(thiophen-2-yl)ethyl]piperidin-4-yl}-N-phenylpropanamide	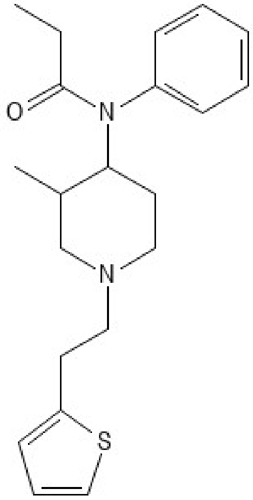	$C_{21}H_{28}N_2OS$	**N**	**N**	**Y**	**N**	**N**

**11**	3-Phenylpropanoylfentanyl	β'-Phenylfentanyl	N,3-Diphenyl-N-[1-(2-phenylethyl)piperidin-4-yl]propanamide	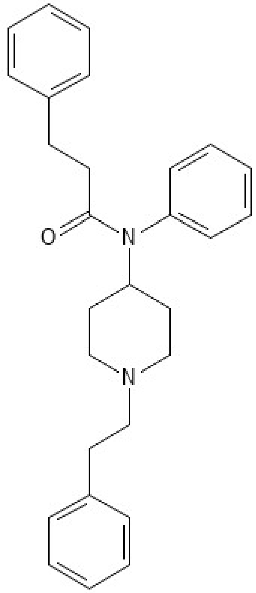	$C_{28}H_{32}N_2O$	**Y**	**N**	**N**	**N**	**N**

**12**	4-Chloroisobutyrfentanyl	Para-chloroisobutyryl fentanyl; 4-Cl-iBF; p-Cl-iBF	N-(4-Chlorophenyl)-2-methyl-N-[1-(2-phenylethyl)piperidin-4-yl]propanamide	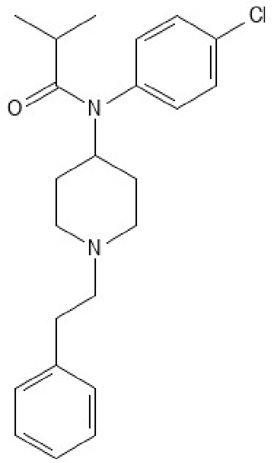	$C_{23}H_{29}ClN_2O$	**Y**	**N**	**N**	**N**	**N**
**13**	4'-Fluoro-Butyrylfentanyl	4-Fluoro-butyrylfentanyl; p-Fluoro-butyrylfentanyl; 4-FBF; p-FBF	N-(4-Fluorophenyl)-N-[1-(2-phenylethyl)piperidin-4-yl]butanamide	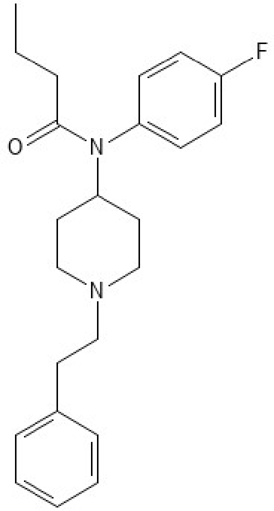	$C_{23}H_{29}FN_2O$	**N**	**N**	**Y**	**N**	**N**

**14**	4-Fluoro-Cyclopropylbenzylfentanyl	Para-fluoro cyclopropyl benzyl fentanyl	N-(1-Benzylpiperidin-4-yl)-N-(4-fluorophenyl)cyclopropanecarboxamide	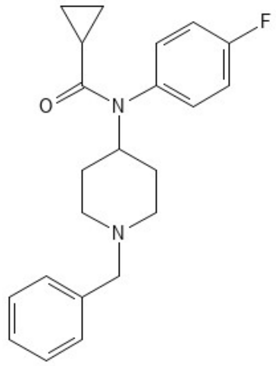	$C_{22}H_{25}FN_2O$	**Y**	**Y**	**N**	**N**	**N**
**15**	4-Fluorofentanyl	Parafluorofentanyl	N-[4-Fluoro-1-(2-phenylethyl)piperidin-4-yl]-N-phenylpropanamide	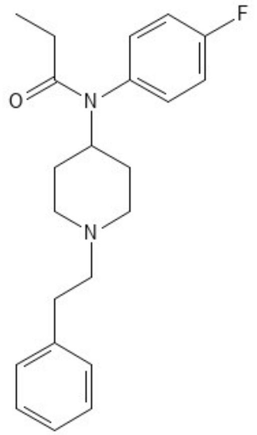	$C_{22}H_{27}FN_2O$	**N**	**Y**	**Y**	**N**	**N**

**16**	4-Fluoroisobutyrfentanyl	para-fluoroisobutyryl fentanyl; 4-F-iBF; 4-FiBF; 4F-iBF; FIBF; p-FIBF; p-FiBF	N-(4-fluorophenyl)-N-(1-phenethylpiperidin-4-yl)isobutyramide	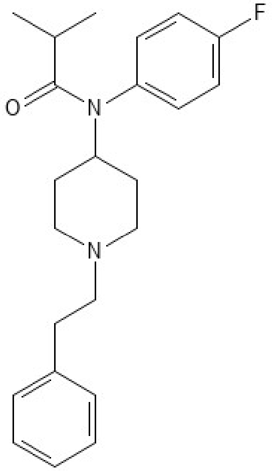	$C_{23}H_{29}FN_2O$	**Y**	**N**	**Y**	**N**	**N**
**17**	4'-Hydroxybutyrylfentanyl	p-Hydroxybutyrylfentanyl; 4-HO-BF	N-(4-Hydroxyphenyl)-N-[1-(2-phenylethyl)piperidin-4-yl]butanamide	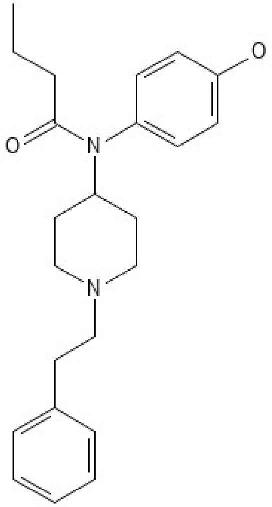	$C_{23}H_{30}N_2O_2$	**N**	**N**	**N**	**N**	**Y**

**18**	4-MeO-Butyrfentanyl	4-methoxy Butyryl fentanyl; p-methoxy Butyryl fentanyl; p-MeO Butyryl fentanyl; 4-MeO-BF	*N*-(4-methoxyphenyl)-*N*-[1-(2-phenylethyl)piperidin-4-yl]butanamide	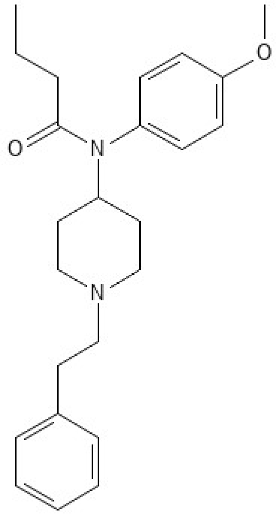	$C_{24}H_{32}N_2O_2$	**Y**	**N**	**N**	**N**	**N**
**19**	4"-Methoxyfentanyl	para-Methoxyfentanyl	*N*-(4-methoxyphenyl)-*N*-[1-(2-phenylethyl)piperidin-4-yl]propanamide	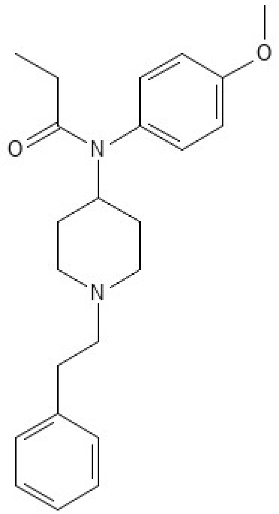	$C_{23}H_{30}N_2O_2$	**Y**	**N**	**N**	**N**	**N**

**20**	4-Methoxymethylfentanyl	R-30490	N-[4-(Methoxymethyl)-1-(2-phenylethyl)piperidin-4-yl]-N-phenylpropanamide	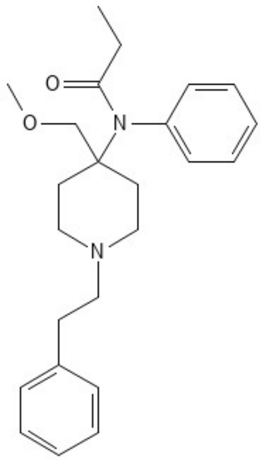	$C_{24}H_{32}N_2O_2$	**N**	**N**	**N**	**N**	**Y**
**21**	4-Phenylfentanyl		N-Phenyl-N-[4-phenyl-1-(2-phenylethyl)piperidin-4-yl]propanamide	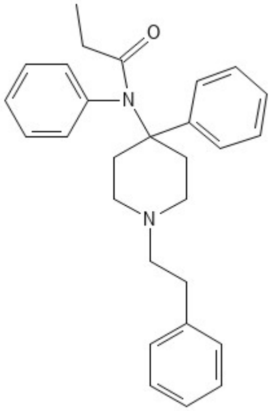	$C_{28}H_{32}N_2O$	**N**	**N**	**N**	**N**	**Y**

**22**	Acetylbenzylfentanyl		*N*-(1-benzylpiperidin-4-yl)-*N*-phenylacetamide	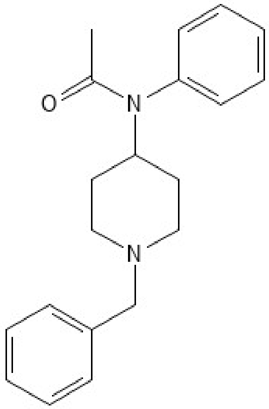	$C_{20}H_{24}N_2O$	**Y**	**N**	**N**	**N**	**N**
**23**	Acetyl-Carfentanil	N-Despropionyl N-Acetyl Carfentanil Methyl Ester	methyl 4-(*N*-acetylanilino)-1-(2-phenylethyl)piperidine-4-carboxylate	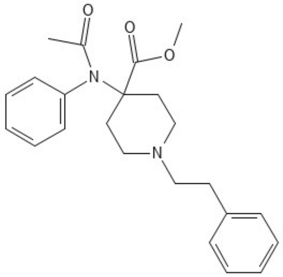	$C_{23}H_{28}N_2O_3$	**N**	**N**	**N**	**N**	**Y**

**24**	Acetylfentanyl	acetyl fentanyl	N-Phenyl-N-[1-(2-phenylethyl)piperidin-4-yl]acetamide	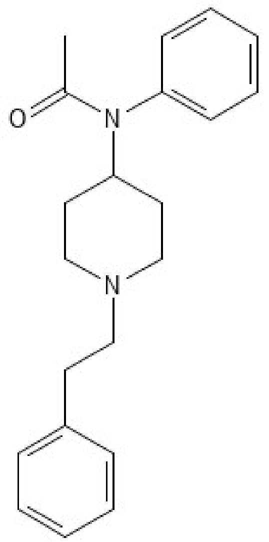	$C_{21}H_{26}N_2O$	**Y**	**Y**	**Y**	**N**	**N**

**25**	Acrylfentanyl	Acryloylfentanyl; Egyptenyl	N-Phenyl-N-[1-(2-phenylethyl)piperidin-4-yl]prop-2-enamide	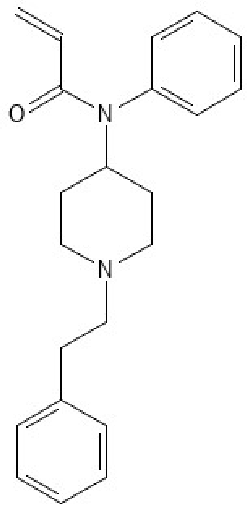	$C_{22}H_{26}N_2O$	**Y**	**Y**	**Y**	**N**	**N**
**26**	Alfentanil	R-39209; Alfenta; Rapifen	N-[1-[2-(4-ethyl-5-oxotetrazol-1-yl)ethyl]-4-(methoxymethyl)piperidin-4-yl]-N-phenylpropanamide	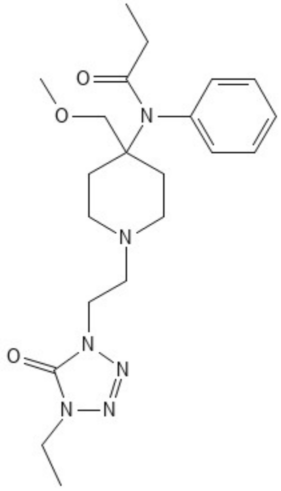	$C_{21}H_{32}N_6O_3$	**N**	**N**	**Y**	**N**	**N**

**27**	Alpha-Methylfentanyl	Alphamethyl fentanyl; α-Methylfentanyl; AMF	N-Phenyl-N-[1-(1-phenylpropan-2-yl)piperidin-4-yl]propanamide	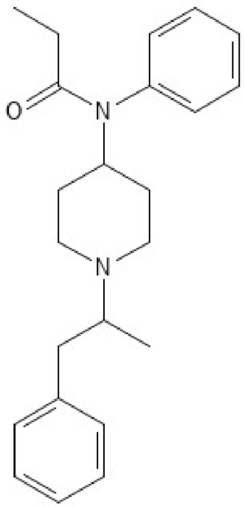	$C_{23}H_{30}N_2O$	**N**	**N**	**Y**	**N**	**N**
**28**	Alpha-Methylfentanyl Butanamide Analogue	BF; B-F	2-methyl-N-phenyl-N-[1-(1-phenylpropan-2-yl)piperidin-4-yl]propanamide	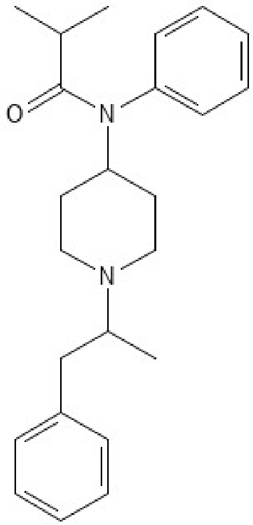	$C_{24}H_{32}N_2O$	**N**	**N**	**N**	**N**	**Y**

**29**	Alpha-Methylthiofentanyl	α-Methyl-thiofentanyl	*N*-phenyl-*N*-[1-(1-thiophen-2-ylpropan-2-yl)piperidin-4-yl]propanamide	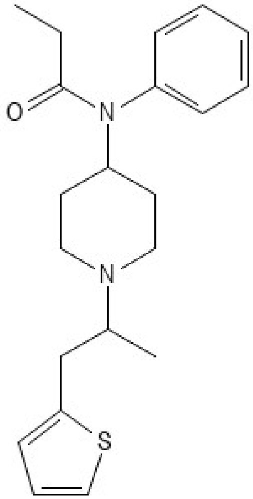	$C_{21}H_{28}N_2OS$	**N**	**N**	**Y**	**N**	**N**
**30**	A-Methylacetylfentanyl	α-Methyl-acetylfentany l	N-Phenyl-N-[1-(1-phenylpropan-2-yl)piperidin-4-yl]acetamide	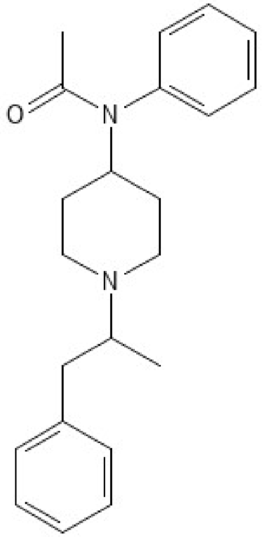	$C_{22}H_{28}N_2O$	**Y**	**N**	**N**	**N**	**N**

**31**	Benzodioxole-Fentanyl	Benzodioxolefentanyl	N-Phenyl-N-[1-(2-phenylethyl)piperidin-4-yl]-2H-1,3-benzodioxole-5-carboxamide	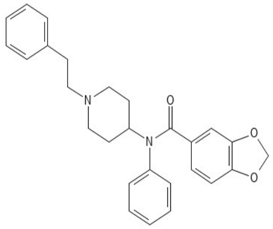	$C_{27}H_{28}N_2O_3$	**Y**	**N**	**N**	**N**	**N**
**32**	Benzofuranyl-Fentanyl		N-{1-[2-(1-Benzofuran-5-yl)ethyl]piperidin-4-yl}-N-phenylpropanamide	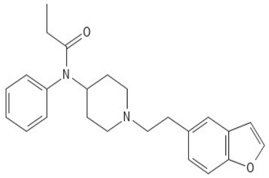	$C_{24}H_{28}N_2O_2$	**N**	**N**	**N**	**N**	**Y**
**33**	Benzoylbenzylfentanyl		*N*-(1-benzylpiperidin-4-yl)-*N*-phenylbenzamide	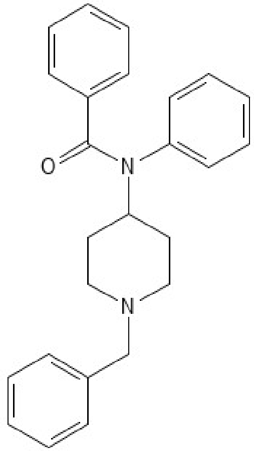	$C_{25}H_{26}N_2O$	**Y**	**N**	**N**	**N**	**N**

**34**	Benzoylfentanyl	Phenylfentanyl	N-Phenyl-N-[1-(2-phenylethyl)piperidin-4-yl]benzamide	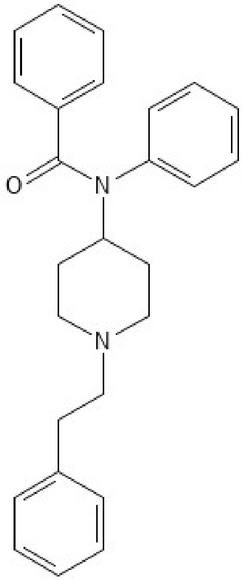	$C_{26}H_{28}N_2O$	**Y**	**Y**	**N**	**N**	**N**
**35**	Benzylfentanyl	N-Benzylfentanyl; R-4129	N-(1-Benzylpiperidin-4-yl)-N-phenylpropanamide	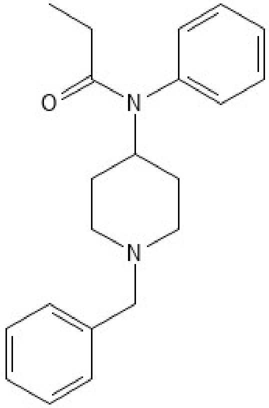	$C_{21}H_{26}N_2O$	**Y**	**N**	**N**	**N**	**N**

**36**	Beta-Hydroxyfentanyl	β-Hydroxyfentanyl; Fentanol	N-[1-(2-Hydroxy-2-phenylethyl)piperidin-4-yl]-N-phenylpropanamide	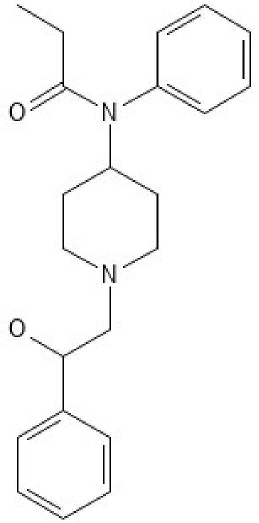	$C_{22}H_{28}N_2O_2$	**N**	**N**	**Y**	**N**	**N**
**37**	B-Hydroxy-3-Methyl-Thienylfentanyl	β-Hydroxy-3-methyl-thienylfentanyl; β-Hydroxy-3-methyl-thiofentanyl	N-{1-[2-Hydroxy-2-(thiophen-2-yl)ethyl]-3-methylpiperidin-4-yl}-N-phenylpropanamide	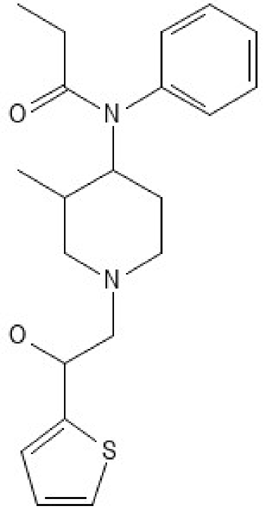	$C_{21}H_{28}N_2O_2S$	**N**	**N**	**N**	**N**	**Y**

**38**	B-Hydroxy-Thiofentanyl	β-Hydroxy-thiofentanyl	N-{1-[2-Hydroxy-2-(thiophen-2-yl)ethyl]piperidin-4-yl}-N-phenylpropanamide	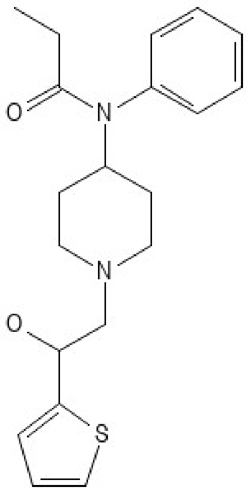	$C_{20}H_{26}N_2O_2S$	**Y**	**N**	**N**	**N**	**N**
**39**	B-Methylfentanyl	β-Methylfentanyl	N-Phenyl-N-[1-(2-phenylpropyl)piperidin-4-yl]propanamide	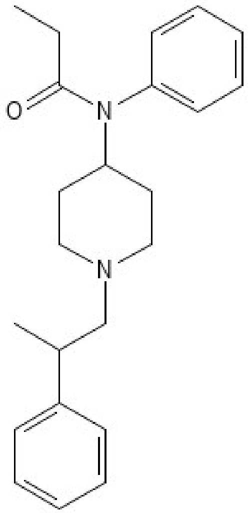	$C_{23}H_{30}N_2O$	**Y**	**N**	**N**	**N**	**N**

**40**	Brifentanil	A-3331	*N*-[(3*R*,4*S*)-1-[2-(4-ethyl-5-oxotetrazol-1-yl)ethyl]-3-methylpiperidin-4-yl]-*N*-(2-fluorophenyl)-2-methoxyacetamide	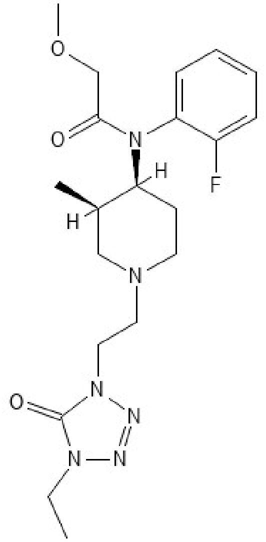	$C_{20}H_{29}FN_6O_3$	**N**	**N**	**N**	**N**	**Y**

**41**	Butyrylfentanyl	Butyrfentanyl; Bu-F; BUF; BF	N-Phenyl-N-[1-(2-phenylethyl)piperidin-4-yl]butanamide	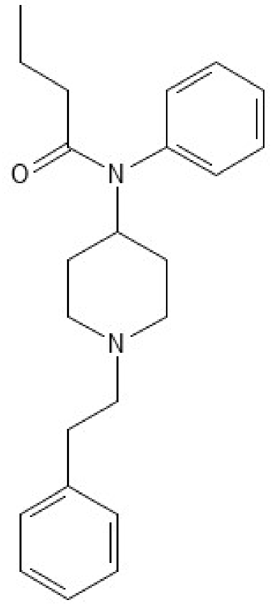	$C_{23}H_{30}N_2O$	**Y**	**Y**	**Y**	**N**	**N**
**42**	Carfentanil	carfentanyl; Wildnil	methyl 1-(2-phenylethyl)-4-(*N*-propanoylanilino)piperidine-4-carboxylate	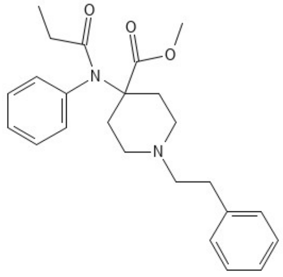	$C_{24}H_{30}N_2O_3$	**Y**	**Y**	**Y**	**N**	**N**

**43**	Crotonylfentanyl		(*E*)-*N*-phenyl-*N*-[1-(2-phenylethyl)piperidin-4-yl]but-2-enamide	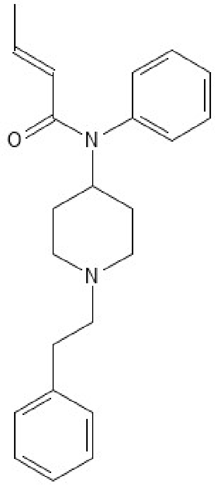	$C_{23}H_{28}N_2O$	**Y**	**Y**	**Y**	**N**	**N**

**44**	Cyclohexylfentanyl		N-Phenyl-N-[1-(2-phenylethyl)piperidin-4-yl]cyclohexanecarboxamide	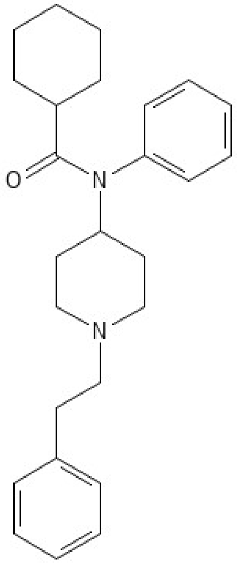	$C_{26}H_{34}N_2O$	**Y**	**N**	**N**	**N**	**N**

**45**	Cyclopentyl-Fentanyl	Cyclopentylfentanyl	N-Phenyl-N-[1-(2-phenylethyl)piperidin-4-yl]cyclopentanecarboxamide	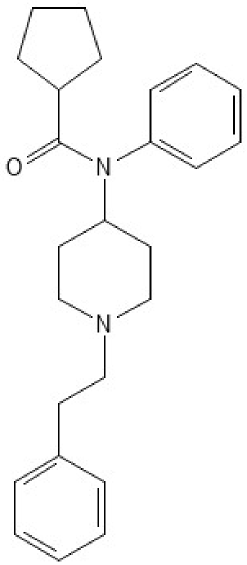	$C_{25}H_{32}N_2O$	**Y**	**N**	**N**	**N**	**N**

**46**	Cyclopropylfentanyl		N-Phenyl-N-[1-(2-phenylethyl)piperidin-4-yl]cyclopropanecarboxamide	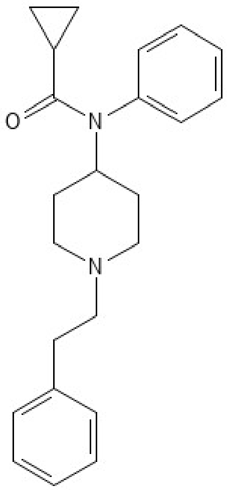	$C_{23}H_{28}N_2O$	**Y**	**Y**	**Y**	**N**	**N**
**47**	Despropionyl-2-Fluoro Fentanyl	Despropionyl-2-Fluorofentanyl; Despropionyl ortho-Fluorofentanyl	*N*-(2-fluorophenyl)-1-(2-phenylethyl)piperidin-4-amine	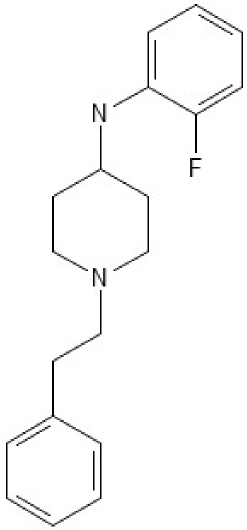	$C_{19}H_{23}FN_2$	**Y**	**N**	**N**	**N**	**N**

**48**	Furanylbenzylfentanyl		N-(1-Benzylpiperidin-4-yl)-N-phenylfuran-2-carboxamide	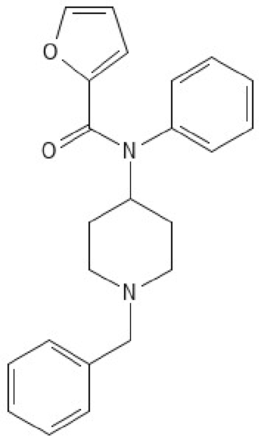	$C_{23}H_{24}N_2O_2$	**N**	**N**	**N**	**N**	**Y**
**49**	Furanylfentanyl	2-Furanylfentanyl; Fu-F; FUF	N-Phenyl-N-[1-(2-phenylethyl)piperidin-4-yl]furan-2-carboxamide	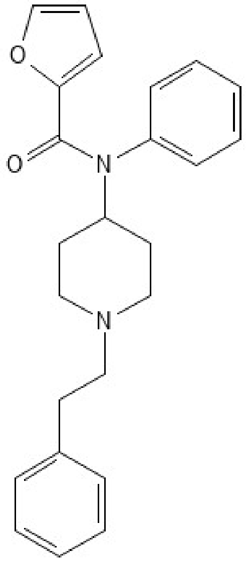	$C_{24}H_{26}N_2O_2$	**Y**	**Y**	**Y**	**N**	**N**

**50**	Furanyl-Norfentanyl		N-Phenyl-N-(piperidin-4-yl)furan-2-carboxamide	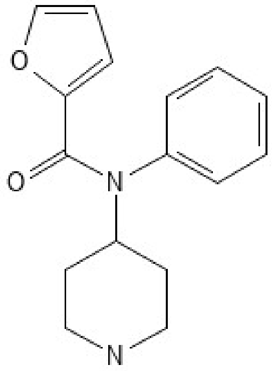	$C_{16}H_{18}N_2O_2$	**N**	**N**	**N**	**N**	**Y**
**51**	Isobutyrylfentanyl	IBF	2-Methyl-N-phenyl-N-[1-(2-phenylethyl)piperidin-4-yl]propanamide	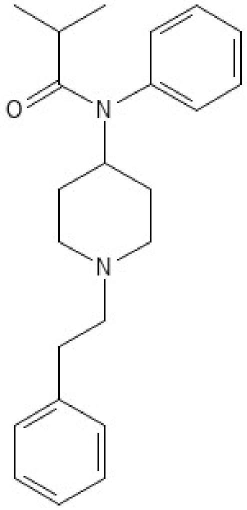	$C_{23}H_{30}N_2O$	**Y**	**N**	**N**	**N**	**N**

**52**	Lofentanil		methyl (3*S*,4*R*)-3-methyl-1-(2-phenylethyl)-4-(*N*-propanoylanilino)piperidine-4-carboxylate	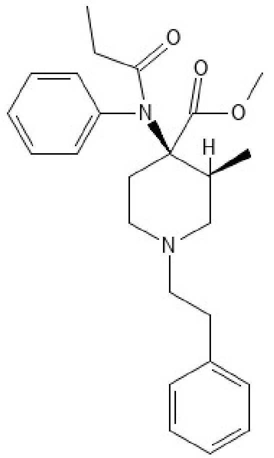	$C_{25}H_{32}N_2O_3$	**N**	**N**	**N**	**N**	**Y**
**53**	Methoxyacetyl-Fentanyl	Methoxyacetylfentanyl; Methoxyacetyl-F; MAF	2-Methoxy-N-phenyl-N-[1-(2-phenylethyl)piperidin-4-yl]acetamide	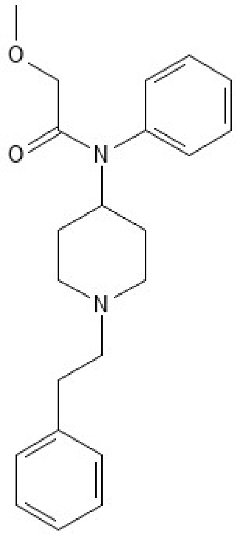	$C_{22}H_{28}N_2O_2$	**Y**	**Y**	**Y**	**N**	**N**

**54**	M-Fluoro-Methoxyacetylfentanyl	m-Fluoro-methoxyacetylfentanyl; 3′-Fluoro-methoxyacetylfentanyl	N-(3-Fluorophenyl)-2-methoxy-N-[1-(2-phenylethyl)piperidin-4-yl]acetamide	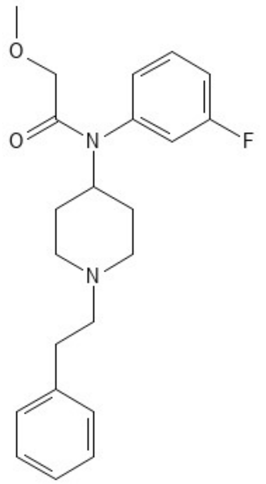	$C_{22}H_{27}FN_2O_2$	**N**	**N**	**N**	**N**	**Y**
**55**	Mirfentanyl		N-[1-(2-Phenylethyl)piperidin-4-yl]-N-(pyrazin-2-yl)furan-2-carboxamide	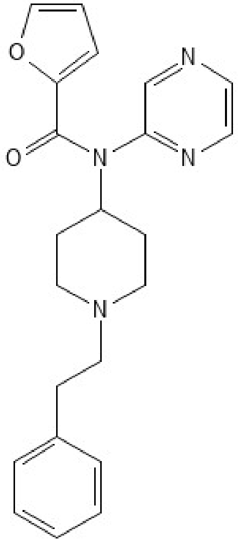	$C_{22}H_{24}N_4O_2$	**N**	**N**	**N**	**N**	**Y**

**56**	M-Methylfentanyl	m-Methylfentanyl; 3′-Methylfentanyl	N-(3-Methylphenyl)-N-[1-(2-phenylethyl)piperidin-4-yl]propanamide	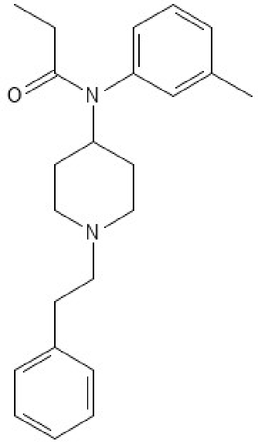	$C_{23}H_{30}N_2O$	**N**	**N**	**N**	**N**	**Y**
**57**	N-Methyl-Carfentanil	R-32395	methyl 1-methyl-4-(*N*-propanoylanilino)piperidine-4-carboxylate	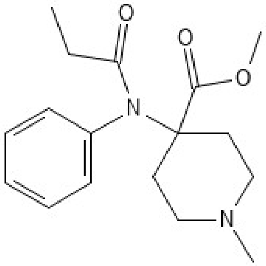	$C_{17}H_{24}N_2O_3$	**N**	**N**	**N**	**N**	**Y**

**58**	N-Methylfentanyl		N-(1-Methylpiperidin-4-yl)-N-phenylpropanamide	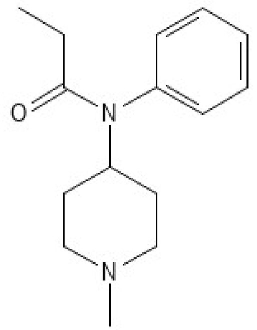	$C_{15}H_{22}N_2O$	**N**	**N**	**N**	**N**	**Y**
**59**	Ocfentanil	A-3217	*N*-(2-fluorophenyl)-2-methoxy-*N*-[1-(2-phenylethyl)piperidin-4-yl]acetamide	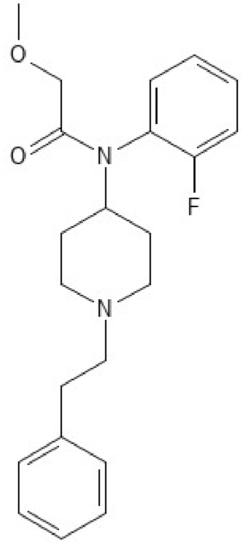	$C_{22}H_{27}FN_2O_2$	**Y**	**N**	**Y**	**N**	**N**

**60**	Ohmefentanyl	β-hydroxy-3-methylfentanyl; OMF; RTI-4614-4	N-[1-(2-hydroxy-2-phenylethyl)-3-methylpiperidin-4-yl]-N-phenylpropanamide	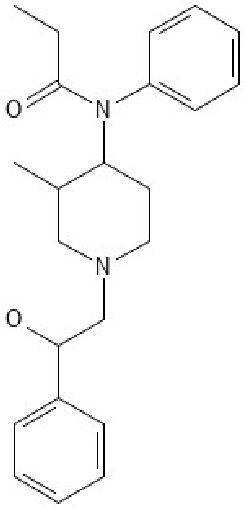	$C_{23}H_{30}N_2O_2$	**N**	**N**	**Y**	**N**	**N**
**61**	O-Isopropyl-Furanylfentanyl	2′-Isopropyl-furanylfentanyl	N-[1-(2-Phenylethyl)piperidin-4-yl]-N-[2-(propan-2-yl)phenyl]furan-2-carboxamide	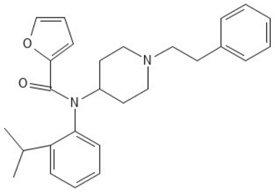	$C_{27}H_{32}N_2O_2$	**Y**	**N**	**N**	**N**	**N**

**62**	O-Methoxy-Furanylfentanyl	2′-Methoxy-furanylfentanyl; o-MeO-Fu-F	N-(2-Methoxyphenyl)-N-[1-(2-phenylethyl)piperidin-4-yl]furan-2-carboxamide	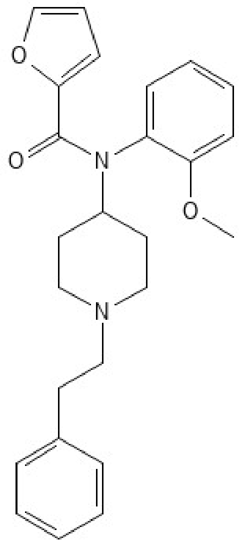	$C_{25}H_{28}N_2O_3$	**Y**	**N**	**N**	**N**	**N**

**63**	O-Methyl-Acetylfentanyl	2′-Methyl-acetylfentanyl; o-iPr-Fu-F	N-(2-Methylphenyl)-N-[1-(2-phenylethyl)piperidin-4-yl]acetamide	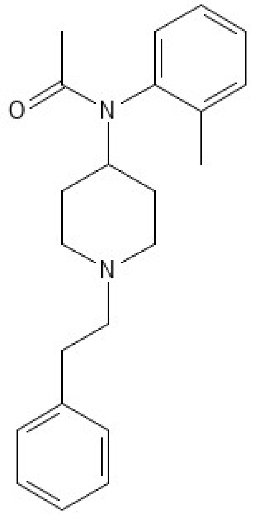	$C_{22}H_{28}N_2O$	**Y**	**N**	**N**	**N**	**N**
**64**	P-Methoxyfentanyl	p-Methoxyfentanyl; 4′-Methoxyfentanyl	N-(4-Methoxyphenyl)-N-[1-(2-phenylethyl)piperidin-4-yl]propanamide	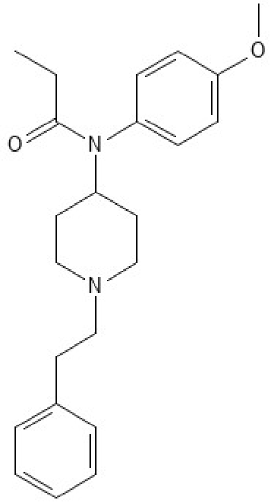	$C_{23}H_{30}N_2O_2$	**Y**	**N**	**N**	**N**	**N**

**65**	P-Methoxy-Furanylfentanyl	p-Methoxy-furanylfentanyl; 4′-Methoxy-furanylfentanyl	*N*-(4-methoxyphenyl)-*N*-[1-(2-phenylethyl)piperidin-4-yl]furan-2-carboxamide	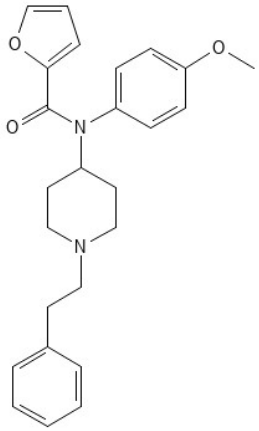	$C_{25}H_{28}N_2O_3$	**Y**	**Y**	**N**	**N**	**N**
**66**	Tetrahydrofuranylfentanyl	Tetrahydrofuran fentanyl; THF-F; THF-F	N-Phenyl-N-[1-(2-phenylethyl)piperidin-4-yl]oxolane-2-carboxamide	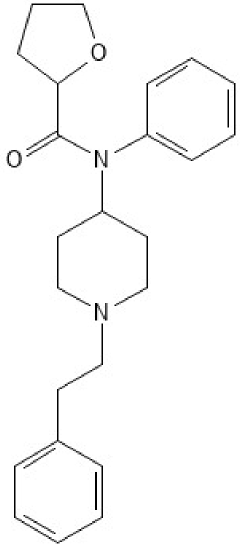	$C_{24}H_{30}N_2O_2$	**Y**	**N**	**Y**	**N**	**N**

**67**	Thenylfentanyl		N-Phenyl-N-{1-[(thiophen-2-yl)methyl]piperidin-4-yl}propanamide	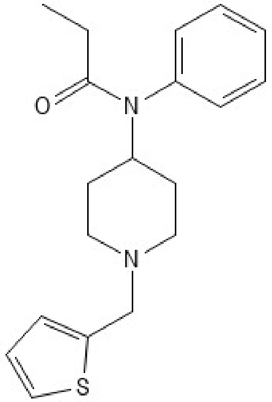	$C_{19}H_{24}N_2OS$	**N**	**N**	**N**	**N**	**Y**
**68**	Thiofentanyl	Thienylfentanil	N-Phenyl-N-{1-[2-(thiophen-2-yl)ethyl]piperidin-4-yl}propanamide	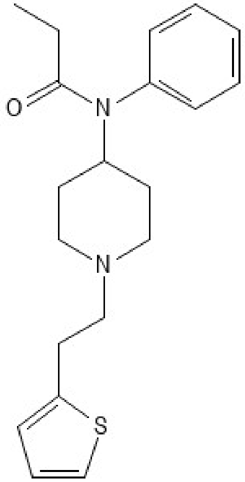	$C_{20}H_{26}N_2OS$	**N**	**N**	**Y**	**N**	**N**

**69**	Thiophenefentanyl	Thiofuranylfentanyl hydrochloride; 2-Thiofuranyl fentanyl; 2-Thiophenoyl fentanyl	*N*-phenyl-*N*-[1-(2-phenylethyl)piperidin-4-yl]thiophene-2-carboxamide;hydrochloride	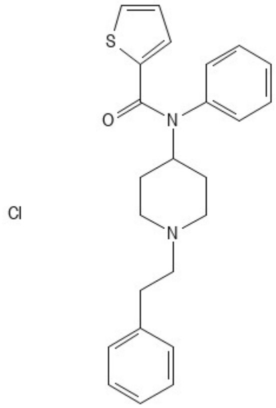	$C_{24}H_{27}ClN_2OS$	**Y**	**N**	**N**	**N**	**N**
**70**	TMCP-F	Tetramethylcyclopropyl-fentanyl	2,2,3,3-Tetramethyl-N-phenyl-N-[1-(2-phenylethyl)piperidin-4-yl]cyclopropane-1-carboxamide	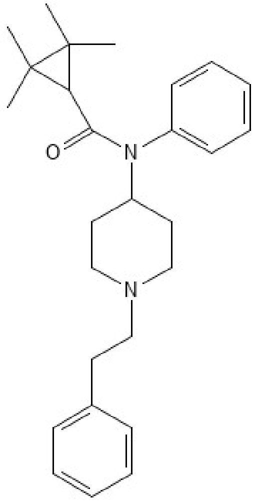	$C_{27}H_{36}N_2O$	**N**	**N**	**N**	**N**	**Y**

**71**	Trefentanil	A-3665	*N*-[1-[2-(4-ethyl-5-oxotetrazol-1-yl)ethyl]-4-phenylpiperidin-4-yl]-*N*-(2-fluorophenyl)propanamide	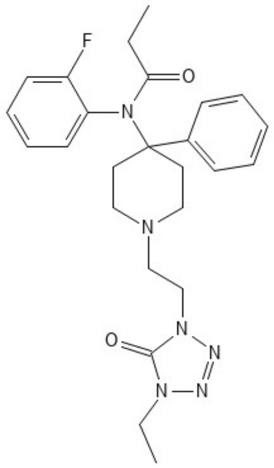	$C_{25}H_{31}FN_6O_2$	**N**	**N**	**N**	**N**	**Y**
**72**	Valerylfentanyl	VF	N-Phenyl-N-[1-(2-phenylethyl)piperidin-4-yl]pentanamide	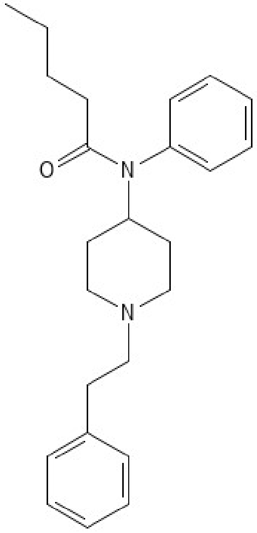	$C_{24}H_{32}N_2O$	**N**	**Y**	**Y**	**N**	**N**

**73**	A-Methyl-Butyrylfentanyl	α-Methyl-butyrylfentanyl; α-Me-BF	N-Phenyl-N-[1-(1-phenylpropan-2-yl)piperidin-4-yl]butanamide	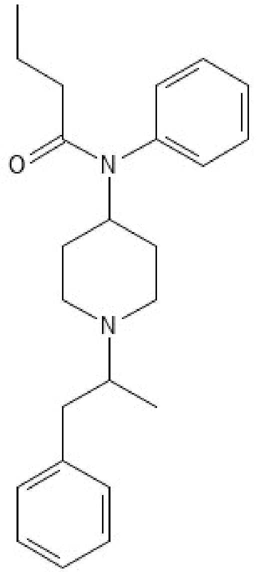	$C_{24}H_{32}N_2O$	**N**	**N**	**N**	**N**	**Y**
**74**	2',2''-Difluorofentanyl	2′,2″-DFF; 2″-Fluoro-o-Fluorofentanyl	N-(2-Fluorophenyl)-N-{1-[2-(2-fluorophenyl)ethyl]piperidin-4-yl}propanamide	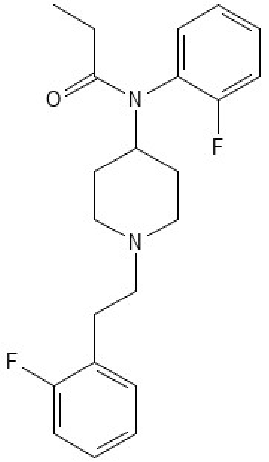	$C_{22}H_{26}F_2N_2O$	**N**	**N**	**N**	**N**	**Y**

**75**	2,3-Secofentanyl		N-{4-[Methyl(2-phenylethyl)amino]butan-2-yl}-N-phenylpropanamide	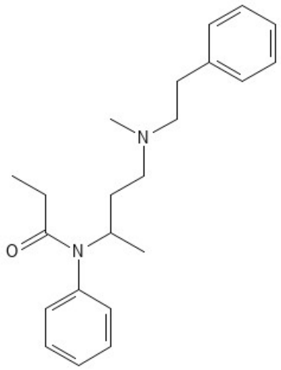	$C_{22}H_{30}N_2O$	**N**	**N**	**N**	**N**	**Y**
**76**	2'-Fluoro-Butyrylfentanyl	o-Fluoro-butyrylfentanyl	N-(2-Fluorophenyl)-N-[1-(2-phenylethyl)piperidin-4-yl]butanamide	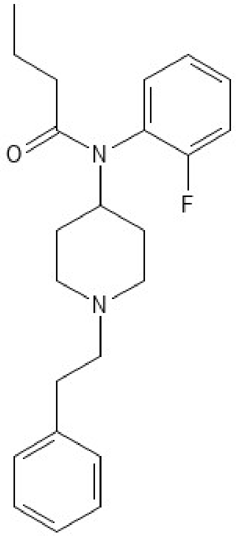	$C_{23}H_{29}FN_2O$	**N**	**N**	**N**	**N**	**Y**

**77**	2'-Fluoro-Isobutyrylfentanyl	o-Fluoro-isobutyrylfentanyl; 2-FIBF	N-(2-Fluorophenyl)-2-methyl-N-[1-(2-phenylethyl)piperidin-4-yl]propanamide	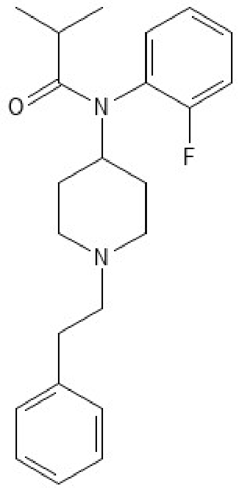	$C_{23}H_{29}FN_2O$	**N**	**N**	**N**	**N**	**Y**
**78**	2-Methyl Carfentanil		Methyl 4-[(2-methylphenyl)(propanoyl)amino]-1-(2-phenylethyl)piperidine-4-carboxylate	N.A	N.A	**N**	**N**	**N**	**N**	**Y**

**79**	3,3-Dimethylfentanyl		N-[3,3-Dimethyl-1-(2-phenylethyl)piperidin-4-yl]-N-phenylpropanamide	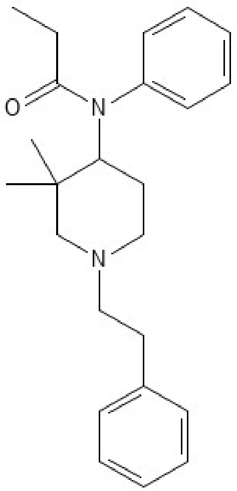	$C_{24}H_{32}N_2O$	**N**	**N**	**N**	**N**	**Y**

**80**	3,5-Dimethyl-Cyclopropylfentanyl		N-[3,5-Dimethyl-1-(2-phenylethyl)piperidin-4-yl]-N-phenylcyclopentanecarboxamide	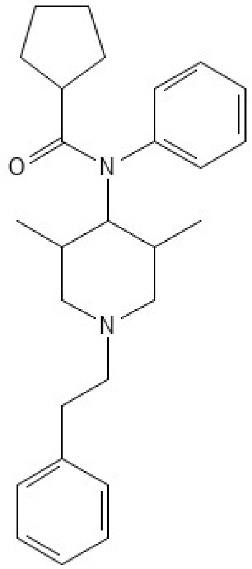	$C_{27}H_{36}N_2O$	**N**	**N**	**N**	**N**	**Y**

**81**	3,5-Dimethylfentanyl		N-[3,5-Dimethyl-1-(2-phenylethyl)piperidin-4-yl]-N-phenylpropanamide	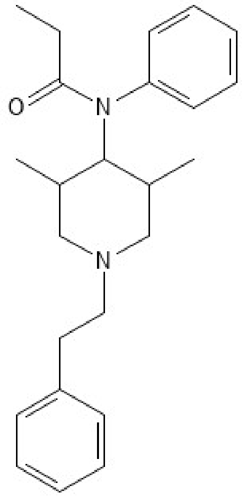	$C_{24}H_{32}N_2O$	**N**	**N**	**N**	**N**	**Y**
**82**	3′-4′-Methylenedioxyfentanyl	MD-F	N-(2H-1,3-Benzodioxol-5-yl)-N-[1-(2-phenylethyl)piperidin-4-yl]propanamide	N.A	N.A	**N**	**N**	**N**	**N**	**Y**

**83**	3′-Me-4F-iBF		N-(4-Fluorophenyl)-2-methyl-N-{1-[2-(3-methylphenyl)ethyl]piperidin-4-yl}propanamide	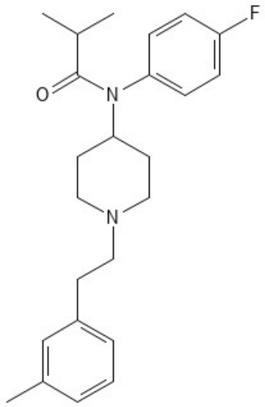	$C_{24}H_{31}FN_2O$	**N**	**N**	**N**	**N**	**Y**
**84**	3′-Methyl-Methoxyacetylfentanyl	m-Methyl-methoxyacetylfentanyl	2-Methoxy-N-(3-methylphenyl)-N-[1-(2-phenylethyl)piperidin-4-yl]acetamide	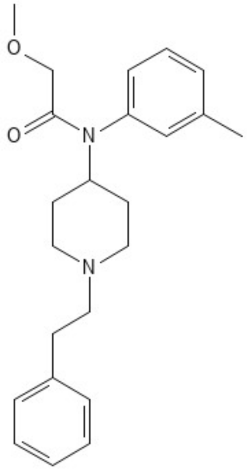	$C_{23}H_{30}N_2O_2$	**N**	**N**	**N**	**N**	**Y**

**85**	3'-4'-Dichloro-3"-Fluorofentanyl		N-(3,4-Dichlorophenyl)-N-{1-[2-(3-fluorophenyl)ethyl]piperidin-4-yl}propanamide	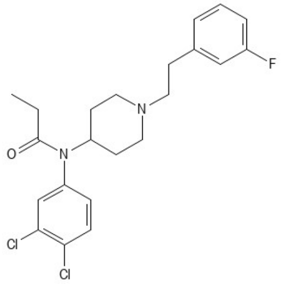	$C_{22}H_{25}Cl_2FN_2O$	**N**	**N**	**N**	**N**	**Y**
**86**	3-Ethylfentanyl		N-[3-Ethyl-1-(2-phenylethyl)piperidin-4-yl]-N-phenylpropanamide	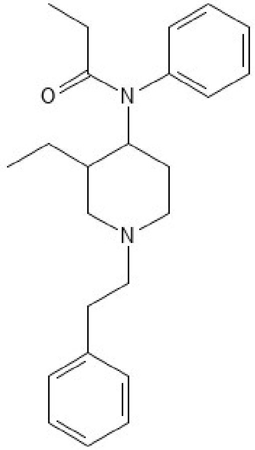	$C_{24}H_{32}N_2O$	**N**	**N**	**N**	**N**	**Y**

**87**	3'-Fluoro-Butyrylfentanyl	m-Fluoro-butyrylfentanyl	N-(3-Fluorophenyl)-N-[1-(2-phenylethyl)piperidin-4-yl]butanamide	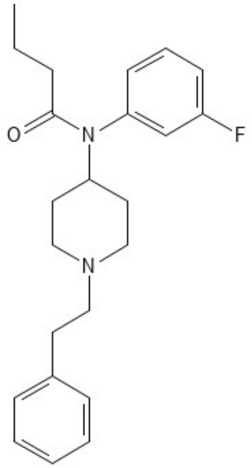	$C_{23}H_{29}FN_2O$	**N**	**N**	**N**	**N**	**Y**
**88**	3'-Fluoro-Isobutyrylfentanyl	m-Fluoro-isobutyrylfentanyl	N-(3-Fluorophenyl)-2-methyl-N-[1-(2-phenylethyl)piperidin-4-yl]propanamide	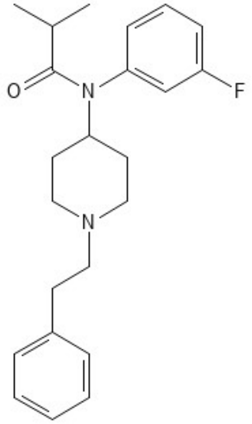	$C_{23}H_{29}FN_2O$	**N**	**N**	**N**	**N**	**Y**

**89**	3-Methoxyfentanyl		N-[3-Methoxy-1-(2-phenylethyl)piperidin-4-yl]-N-phenylpropanamide	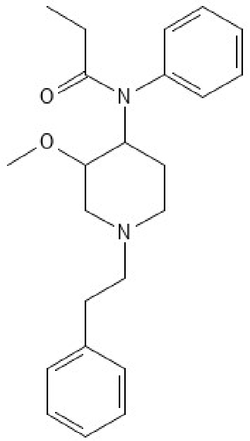	$C_{23}H_{30}N_2O_2$	**N**	**N**	**N**	**N**	**Y**
**90**	3-Methyl Phenoxy Acetylfentanil		N-[3-Methyl-1-(2-phenoxyethyl)piperidin-4-yl]-N-phenylacetamide	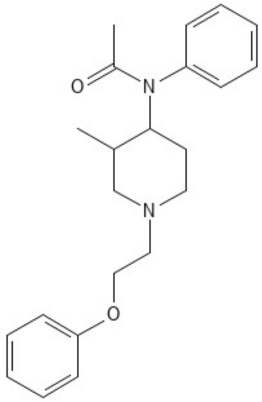	$C_{22}H_{28}N_2O_2$	**N**	**N**	**N**	**N**	**Y**

**91**	3-Methyl-Furanylfentanyl	3MFUF; TMFUF	N-[3-Methyl-1-(2-phenylethyl)piperidin-4-yl]-N-phenylfuran-2-carboxamide	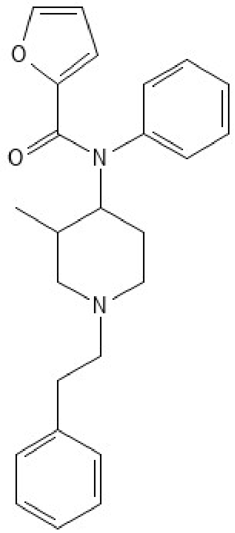	$C_{25}H_{28}N_2O_2$	**N**	**N**	**N**	**N**	**Y**
**92**	4-(m-Hydroxyphenyl)Fentanyl		N-[4-(3-Hydroxyphenyl)-1-(2-phenylethyl)piperidin-4-yl]-N-phenylpropanamide	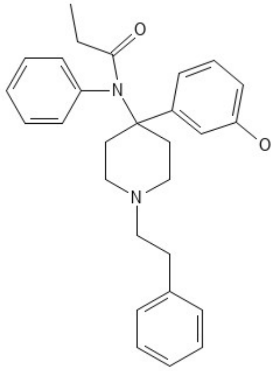	$C_{28}H_{32}N_2O_2$	**N**	**N**	**N**	**N**	**Y**

**93**	4′-Chloro-Butyrylfentanyl	4Cl-Butyryl fentanyl; p-Chloro-butyrylfentanyl; p-CBF	N-(4-Chlorophenyl)-N-[1-(2-phenylethyl)piperidin-4-yl]butanamide	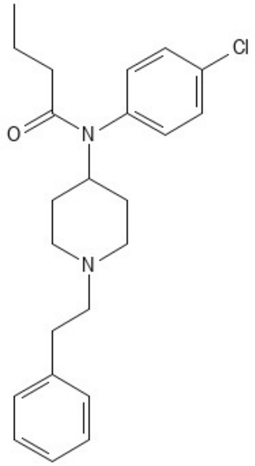	$C_{23}H_{29}ClN_2O$	**N**	**N**	**N**	**N**	**Y**
**94**	4′-Methylfentanyl	p-Methylfentanyl; 4′-MF	N-(4-Methylphenyl)-N-[1-(2-phenylethyl)piperidin-4-yl]propanamide	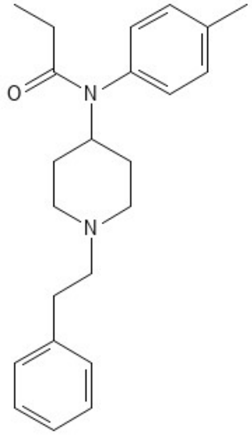	$C_{23}H_{30}N_2O$	**Y**	**N**	**N**	**N**	**N**

**95**	4′-Methyl-Methoxyacetylfentanyl	p-Methyl-methoxyacetylfentanyl	2-Methoxy-N-(4-methylphenyl)-N-[1-(2-phenylethyl)piperidin-4-yl]acetamide	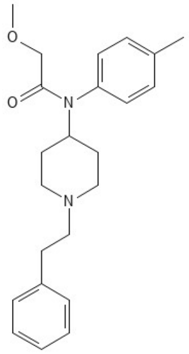	$C_{23}H_{30}N_2O_2$	**N**	**N**	**N**	**N**	**Y**
**96**	4′-Methyl-Tetrahydrofuranylfentanyl	p-Methyl-tetrahydrofuranylfentanyl	N-(4-Methylphenyl)-N-[1-(2-phenylethyl)piperidin-4-yl]oxolane-2-carboxamide	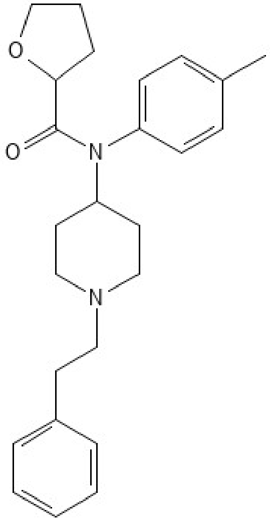	$C_{25}H_{32}N_2O_2$	**N**	**Y**	**N**	**N**	**N**

**97**	4″-Nitrofentanyl		N-{1-[2-(4-Nitrophenyl)ethyl]piperidin-4-yl}-N-phenylpropanamide	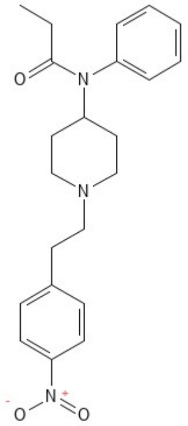	$C_{22}H_{27}N_3O_3$	**N**	**N**	**N**	**N**	**Y**
**98**	4-Anilino-N-phenylethyl-4-piperidine	4-ANPP; 1-Phenethyl-N-phenylpiperidin-4-amine	N-phenyl-1-(2-phenylethyl)piperidin-4-amine	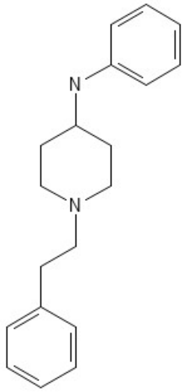	$C_{19}H_{24}N_2$	**Y**	**N**	**N**	**N**	**N**

**99**	4"-Bromo-Ohmefentanyl		*N*-[1-[2-(4-bromophenyl)-2-hydroxyethyl]-3-methylpiperidin-4-yl]-*N*-phenylpropanamide;hydrochloride	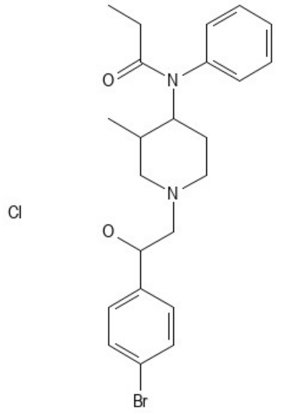	$C_{23}H_{30}BrClN_2O_2$	**N**	**N**	**N**	**N**	**Y**
**100**	4'-Chloro-Cyclobutylfentanyl	p-Chloro-cyclobutylfentanyl	N-(4-Chlorophenyl)-N-[1-(2-phenylethyl)piperidin-4-yl]cyclobutanecarboxamide	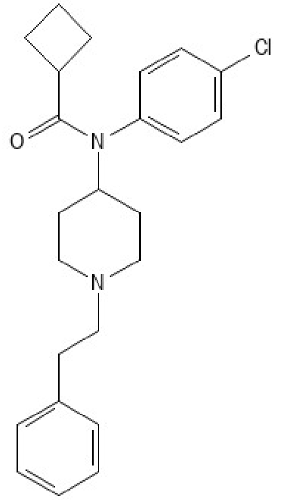	$C_{24}H_{29}ClN_2O$	**N**	**N**	**N**	**N**	**Y**

**101**	4'-Chloro-Cyclopentylfentanyl	p-Chloro-cyclopentylfentanyl	N-(4-Chlorophenyl)-N-[1-(2-phenylethyl)piperidin-4-yl]cyclopentanecarboxamide	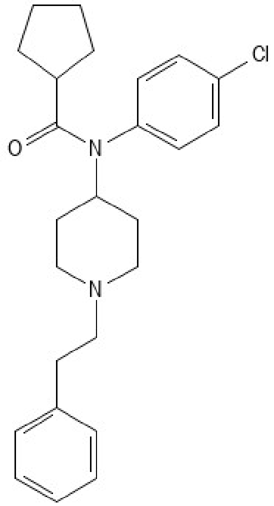	$C_{25}H_{31}ClN_2O$	**N**	**N**	**N**	**N**	**Y**
**102**	4'-Chloro-Cyclopropylfentanyl	p-Chloro-cyclopropylfentanyl; 4′-Cl-Cyclopropyl-F	N-(4-Chlorophenyl)-N-[1-(2-phenylethyl)piperidin-4-yl]cyclopropanecarboxamide	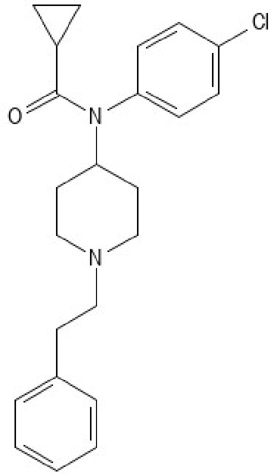	$C_{23}H_{27}ClN_2O$	**N**	**N**	**N**	**N**	**Y**

**103**	4'-Chlorofentanyl	p-Chlorofentanyl; p-CF	N-(4-Chlorophenyl)-N-[1-(2-phenylethyl)piperidin-4-yl]propanamide	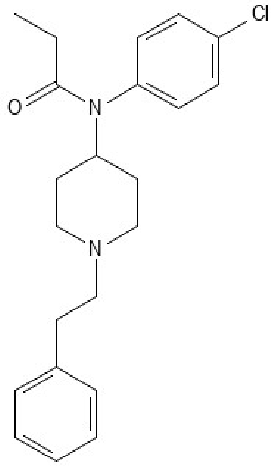	$C_{22}H_{27}ClN_2O$	**Y**	**N**	**N**	**N**	**N**
**104**	4'-Fluoro-Acetylfentanyl	p-Fluoro-acetylfentanyl; p-F-AF	N-(4-Fluorophenyl)-N-[1-(2-phenylethyl)piperidin-4-yl]acetamide	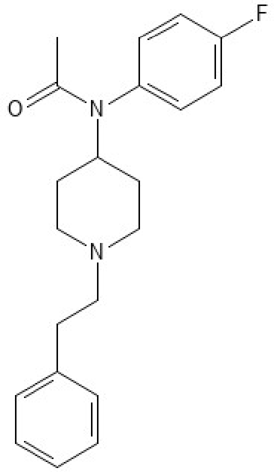	$C_{21}H_{25}FN_2O$	**Y**	**N**	**N**	**N**	**N**

**105**	4'-Fluoro-Acrylfentanyl	p-Fluoro-acrylfentanyl	N-(4-Fluorophenyl)-N-[1-(2-phenylethyl)piperidin-4-yl]prop-2-enamide	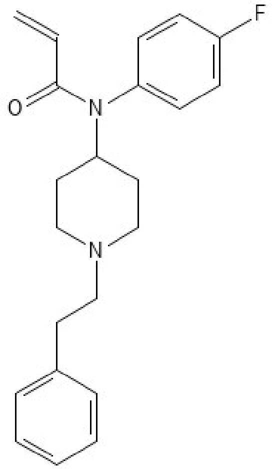	$C_{22}H_{25}FN_2O$	**N**	**N**	**N**	**N**	**Y**
**106**	4"-Fluorofentanyl		N-{1-[2-(4-Fluorophenyl)ethyl]piperidin-4-yl}-N-phenylpropanamide	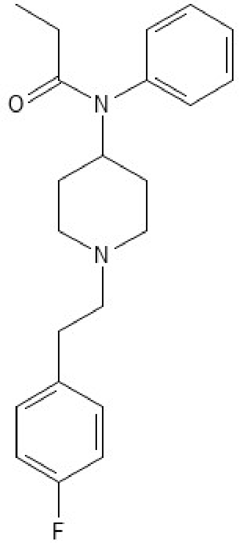	$C_{22}H_{27}FN_2O$	**N**	**N**	**N**	**N**	**Y**

**107**	4''-Fluoro-Ohmefentanyl		N-{1-[2-(4-Fluorophenyl)-2-hydroxyethyl]-3-methylpiperidin-4-yl}-N-phenylpropanamide	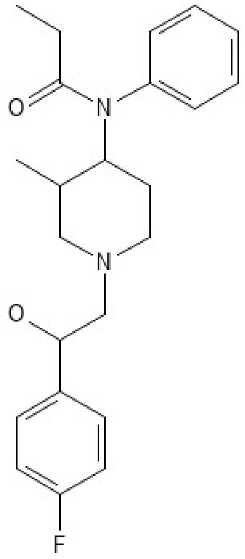	$C_{23}H_{29}FN_2O_2$	**N**	**N**	**N**	**N**	**Y**
**108**	4'-Fluoro-β-Hydroxy-Thiobutyrylfentanyl	p-Fluoro-β-hydroxy-thiobutyrylfentanyl; BHT-FBF	N-(4-Fluorophenyl)-N-{1-[2-hydroxy-2-(thiophen-2-yl)ethyl]piperidin-4-yl}butanamide	N.A	N.A	**N**	**N**	**N**	**N**	**Y**

**109**	4-Methyl Furanyl Fentanyl	p-methyl Furanyl fentanyl; para-methyl Fu-F; p-methyl Fu-F; 4-methyl Fu-F	*N*-(4-methylphenyl)-*N*-[1-(2-phenylethyl)piperidin-4-yl]furan-2-carboxamide	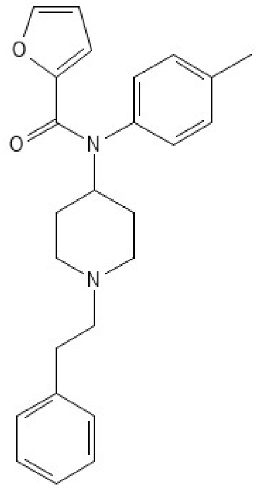	$C_{25}H_{28}N_2O_2$	**N**	**N**	**N**	**N**	**Y**
**110**	4''-Methyl-Acetylfentanyl	4-methylphenethylacetylfentanyl	N-{1-[2-(4-Methylphenyl)ethyl]piperidin-4-yl}-N-phenylacetamide	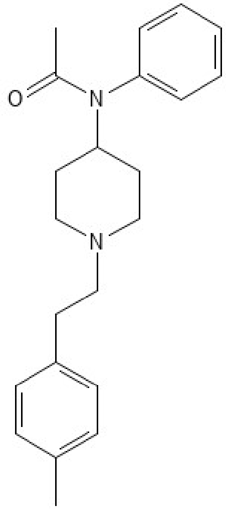	$C_{22}H_{28}N_2O$	**N**	**N**	**N**	**N**	**Y**

**111**	4"-Methylfentanyl		N-{1-[2-(4-Methylphenyl)ethyl]piperidin-4-yl}-N-phenylpropanamide	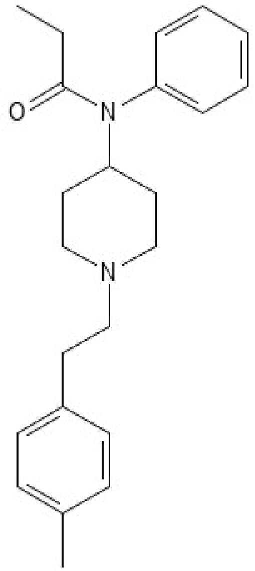	$C_{23}H_{30}N_2O$	**N**	**N**	**N**	**N**	**Y**
**112**	4-Methylfentanyl		N-[4-Methyl-1-(2-phenylethyl)piperidin-4-yl]-N-phenylpropanamide	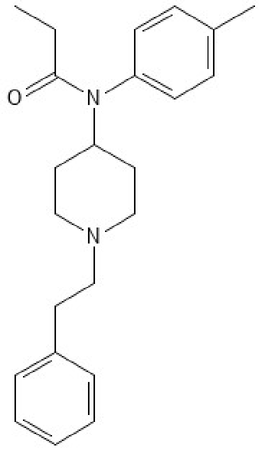	$C_{23}H_{30}N_2O$	**Y**	**N**	**N**	**N**	**N**

**113**	A,3-Dimethylfentanyl	α,3-Dimethylfentanyl	N-[3-Methyl-1-(1-phenylpropan-2-yl)piperidin-4-yl]-N-phenylpropanamide	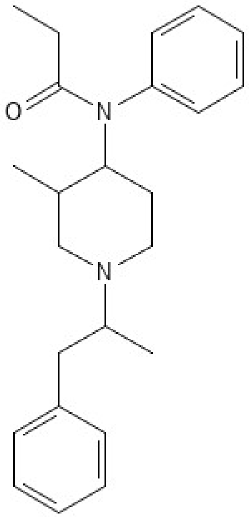	$C_{24}H_{32}N_2O$	**N**	**N**	**N**	**N**	**Y**

**114**	A'-Methoxyfentanyl	α′-Methoxyfentanyl	2-Methoxy-N-phenyl-N-[1-(2-phenylethyl)piperidin-4-yl]propanamide	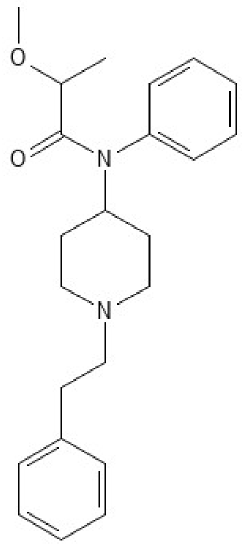	$C_{23}H_{30}N_2O_2$	**N**	**N**	**N**	**N**	**Y**

**115**	A-Methyl-Acrylfentanyl	α-Methyl-acrylfentanyl; Acryloyl-α-methylfentanyl	N-Phenyl-N-[1-(1-phenylpropan-2-yl)piperidin-4-yl]prop-2-enamide	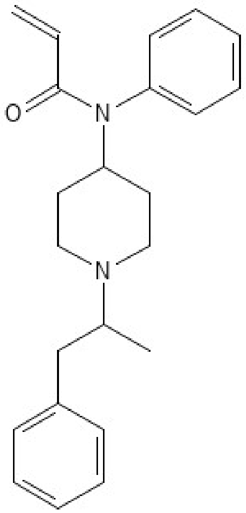	$C_{23}H_{28}N_2O$	**N**	**N**	**N**	**N**	**Y**
**116**	A'-Methyl-Butyrylfentanyl	α′-Methyl-butyrylfentanyl	2-Methyl-N-phenyl-N-[1-(2-phenylethyl)piperidin-4-yl]butanamide	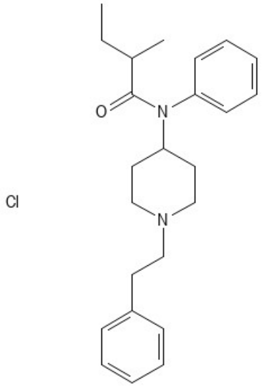	$C_{24}H_{33}ClN_2O$	**Y**	**N**	**N**	**N**	**N**

**117**	A-Methyl-P-Fluorofentanyl	α-Methyl-p-fluorofentanyl; AM-p-F-F	N-(4-Fluorophenyl)-N-[1-(1-phenylpropan-2-yl)piperidin-4-yl]propanamide	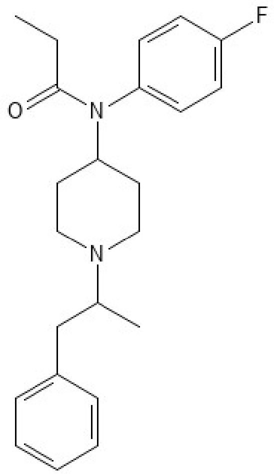	$C_{23}H_{29}FN_2O$	**N**	**N**	**N**	**N**	**Y**
**118**	B-Hydroxy-Carfentanil	β-Hydroxy-carfentanil	methyl 1-(2-hydroxy-2-phenylethyl)-4-(*N*-propanoylanilino)piperidine-4-carboxylate	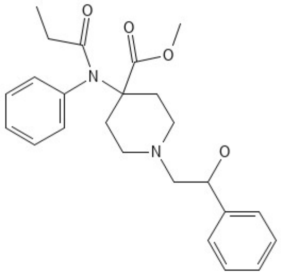	$C_{24}H_{30}N_2O_4$	**N**	**N**	**N**	**N**	**Y**

**119**	B-Hydroxy-P-Fluorofentanyl	β-Hydroxy-p-fluorofentanyl; BH-p-F-F	N-(4-Fluorophenyl)-N-[1-(2-hydroxy-2-phenylethyl)piperidin-4-yl]propanamide	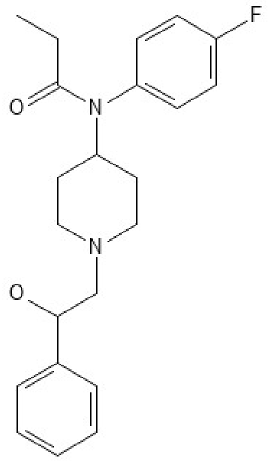	$C_{22}H_{27}FN_2O_2$	**N**	**N**	**N**	**N**	**Y**
**120**	B-Hydroxy-Sufentanil	β-Hydroxy-sufentanil	N-{1-[2-Hydroxy-2-(thiophen-2-yl)ethyl]-4-(methoxymethyl)piperidin-4-yl}-N-phenylpropanamide	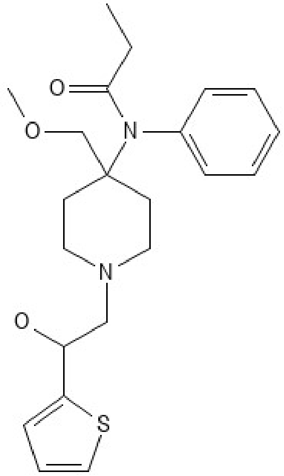	$C_{22}H_{30}N_2O_3S$	**N**	**N**	**N**	**N**	**Y**

**121**	Butyryl-Carfentanyl		Methyl 4-[butanoyl(phenyl)amino]-1-(2-phenylethyl)piperidine-4-carboxylate	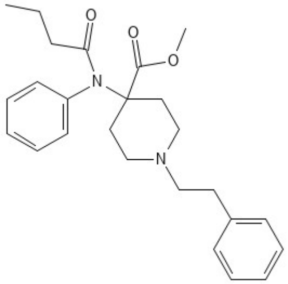	$C_{25}H_{32}N_2O_3$	**N**	**N**	**N**	**N**	**Y**
**122**	Butyrylremifentanil		Methyl 4-[butanoyl(phenyl)amino]-1-(3-methoxy-3-oxopropyl)piperidine-4-carboxylate	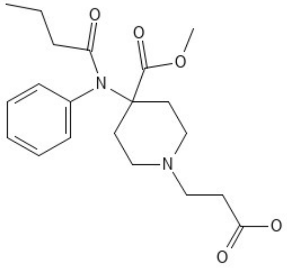	$C_{20}H_{28}N_2O_5$	**N**	**N**	**N**	**N**	**Y**

**123**	Cyclobutylfentanyl		N-Phenyl-N-[1-(2-phenylethyl)piperidin-4-yl]cyclobutanecarboxamide	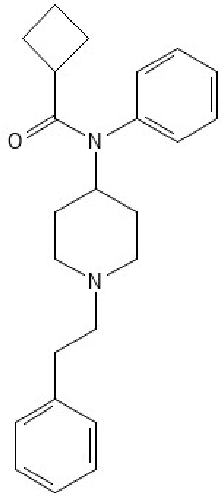	$C_{24}H_{30}N_2O$	**N**	**N**	**N**	**N**	**Y**
**124**	Cyclopentenylfentanyl		N-Phenyl-N-[1-(2-phenylethyl)piperidin-4-yl]cyclopent-1-ene-1-carboxamide	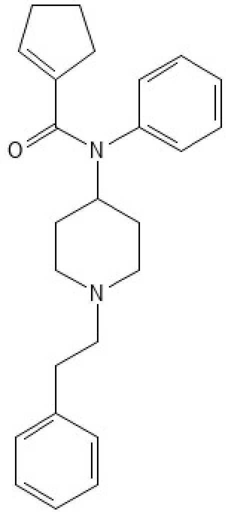	$C_{25}H_{30}N_2O$	**N**	**N**	**N**	**N**	**Y**

**125**	Ethyl [1-(2-Hydroxy-2-Phenylethyl)-3-Methylpiperidin-4-Yl]Phenylcarbamate		Ethyl [1- (2-Hydroxy-2-Phenylethyl)-3-Methylpiperidin-4-Yl]Phenylcarbamate	N.A	N.A	**N**	**N**	**N**	**N**	**Y**
**126**	Fentanyl	fentanil; Actiq; Duragesic; Fentora	N-phenyl-N-[1-(2-phenylethyl)piperidin-4-yl]propanamide	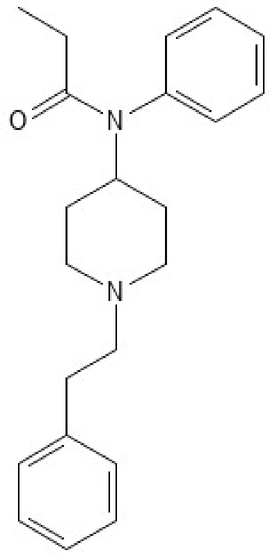	$C_{22}H_{28}N_2O$	**N**	**N**	**Y**	**N**	**N**

**127**	Fentranyl	trans-Phenylcyclopropyl-norfentanyl	N-Phenyl-N-[1-(2-phenylcyclopropyl)piperidin-4-yl]propanamide	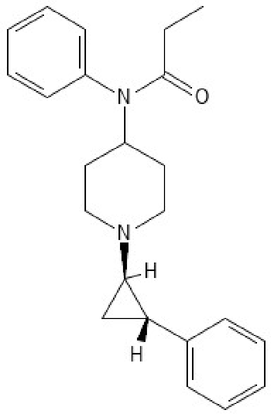	$C_{23}H_{28}N_2O$	**N**	**N**	**N**	**N**	**Y**
**128**	Fluoropentyl-Norcarfentanil		Methyl 1-(5-fluoropentyl)-4-[phenyl(propanoyl)amino]piperidine-4-carboxylate	N.A	N.A	**N**	**N**	**N**	**N**	**Y**
**129**	Isocarfentanyl	3-Carbomethoxyfentanyl; 3-Methoxycarbonylfentanyl	Methyl 1-(2-phenylethyl)-4-[phenyl(propanoyl)amino]piperidine-3-carboxylate	N.A	N.A	**N**	**N**	**N**	**N**	**Y**

**130**	Isofentanyl	N-Benzyl-3-methylfentanyl; 3-Methyl-benzylfentanyl	N-(1-Benzyl-3-methylpiperidin-4-yl)-N-phenylpropanamide	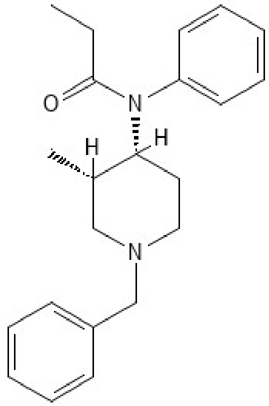	$C_{22}H_{28}N_2O$	**N**	**N**	**N**	**N**	**Y**
**131**	Isovaleroylfentanyl	Isovalerylfentanyl	3-Methyl-N-phenyl-N-[1-(2-phenylethyl)piperidin-4-yl]butanamide	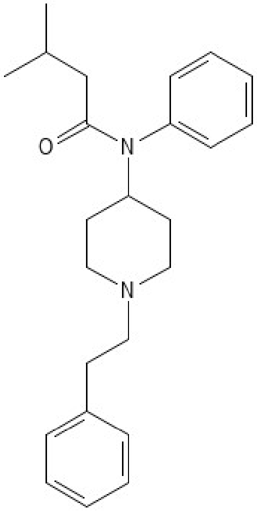	$C_{24}H_{32}N_2O$	**N**	**N**	**N**	**N**	**Y**

**132**	Methacroylfentanyl		2-Methyl-N-phenyl-N-[1-(2-phenylethyl)piperidin-4-yl]prop-2-enamide	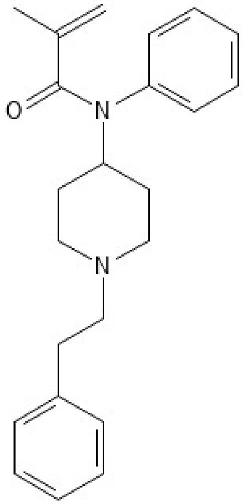	$C_{23}H_{28}N_2O$	**N**	**N**	**N**	**N**	**Y**
**133**	Methyl 1-(2-Hydroxy-2-Phenylethyl)-3-Methyl-4-[Phenyl (Propanoyl)Amino]Piperidine-4-Carboxylate		Methyl 1-(2-hydroxy-2-phenylethyl)-3-methyl-4-[phenyl(propanoyl)amino]piperidine-4-carboxylate	N.A	N.A	**N**	**N**	**N**	**N**	**Y**
**134**	Methyl 1-[(2,3-Dihydro-1,4-Benzodioxin-2-Yl)Methyl]-4-[Phenyl (Propanoyl)Amino]Piperidine-4-Carboxylate		Methyl 1-[(2,3-dihydro-1,4-benzodioxin-2-yl)methyl]-4-[phenyl(propanoyl)amino]piperi	N.A	N.A	**N**	**N**	**N**	**N**	**Y**

			dine-4-carboxylate							
**135**	Methyl 1-[2-(2-Oxo-1,3-Benzoxazol-3(2h)-Yl)Ethyl]-4-[Phenyl(Propanoyl)Amino]Piperidine-4-Carboxylate		Methyl 1-[2-(2-oxo-1,3-benzoxazol-3(2H)-yl)ethyl]-4-[phenyl(propanoyl)amino]piperidine-4-carboxylate	N.A	N.A	**N**	**N**	**N**	**N**	**Y**
**136**	Methyl 1-[2-(2-Oxo-2,3-Dihydro-1h-Indol-1-Yl)Ethyl]-4-[Phenyl(Propanoyl)Amino]Piperidine-4-Carboxylate		Methyl 1-[2-(2-oxo-2,3-dihydro-1H-indol-1-yl)ethyl]-4-[phenyl(propanoyl)amino]piperidine-4-carboxylate	N.A	N.A	**N**	**N**	**N**	**N**	**Y**
**137**	Methyl 1-[2-(3-Oxo-2,3-Dihydro-4h-1,4-Benzothiazin-4-Yl)Ethyl]-4-[Phenyl(Propanoyl)Amino]Piperidine-4-Carboxylate		Methyl 1-[2-(3-oxo-2,3-dihydro-4H-1,4-benzothiazin-4-yl)ethyl]-4-[phenyl(propanoyl)amino]piperi	N.A	N.A	**N**	**N**	**N**	**N**	**Y**

			dine-4-carboxylate							
**138**	Methyl 1-[2-(4-Methyl-1,3-Thiazol-5-Yl)Ethyl]-4-[Phenyl (Propanoyl)Amino]Piperidine-4-Carboxylate		Methyl 1-[2-(4-methyl-1,3-thiazol-5-yl)ethyl]-4-[phenyl(propanoyl)amino]piperidine-4-carboxylate	N.A	N.A	**N**	**N**	**N**	**N**	**Y**
**139**	Methyl 1-[2-Hydroxy-2-(Thiophen-2-Yl)Ethyl]-4-[Phenyl (Propanoyl)Amino]Piperidine-4-Carboxylate		Methyl 1-[2-hydroxy-2-(thiophen-2-yl)ethyl]-4-[phenyl(propanoyl)amino]piperidine-4-carboxylate	N.A	N.A	**N**	**N**	**N**	**N**	**Y**
**140**	Methyl 1-[2-Oxo-2-(Thiophen-2-Yl)Ethyl]-4-[Phenyl (Propanoyl)Amino]Piperidine-4-Carboxylate		Methyl 1-[2-Oxo-2-(Thiophen-2-Yl)Ethyl]-4-[Phenyl (Propanoyl)Amino]Piperidine-4-Carboxylate	N.A	N.A	**N**	**N**	**N**	**N**	**Y**

**141**	Methyl 1-{2-[(1-Methyl-1h-Imidazol-2-Yl)Sulfanyl]Ethyl}-4-[Phenyl (Propanoyl)Amino]Piperidine-4-Carboxylate		Methyl 1-{2-[(1-Methyl-1h-Imidazol-2-Yl)Sulfanyl]Ethyl}-4-[Phenyl (Propanoyl)Amino]Piperidine-4-Carboxylate	N.A	N.A	**N**	**N**	**N**	**N**	**Y**
**142**	Methyl 1-{2-[5-Methyl-2-(Methylsulfanyl)-6-Oxopyrimidin-1 (6h)-Yl]Ethyl}-4-[Phenyl (Propanoyl)Amino]Piperidine-4-Carboxylate		Methyl 1-{2-[5-Methyl-2-(Methylsulfanyl)-6-Oxopyrimidin-1 (6h)-Yl]Ethyl}-4-[Phenyl (Propanoyl)Amino]Piperidine-4-Carboxylate	N.A	N.A	**N**	**N**	**N**	**N**	**Y**
**143**	Methyl 4-[Phenyl (Propanoyl)Amino]-1-[2-(1h-Pyrazol-1-Yl)Ethyl]Piperidine-4-Carboxylate		Methyl 4-[Phenyl (Propanoyl)Amino]-1-[2-(1h-Pyrazol-1-Yl)Ethyl]Piperidine-4-Carboxylate	N.A	N.A	**N**	**N**	**N**	**N**	**Y**
**144**	Methyl 4-[Phenyl (Propanoyl)Amino]-1-		Methyl 4-[Phenyl	N.A	N.A	**N**	**N**	**N**	**N**	**Y**

	[2-(1h-Pyrrol-1-Yl)Ethyl]Piperidine-4-Carboxylate		(Propanoyl)Amino]-1-[2-(1h-Pyrrol-1-Yl)Ethyl]Piperidine-4-Carboxylate							
**145**	Methyl 4-[Phenyl (Propanoyl)Amino]-1-[2-(2h-Tetrazol-2-Yl)Ethyl]Piperidine-4-Carboxylate		Methyl 4-[Phenyl (Propanoyl)Amino]-1-[2-(2h-Tetrazol-2-Yl)Ethyl]Piperidine-4-Carboxylate	N.A	N.A	**N**	**N**	**N**	**N**	**Y**
**146**	Methyl 4-[Phenyl (Propanoyl)Amino]-1-[2-(Pyridin-2-Yl)Ethyl]Piperidine-4-Carboxylate		Methyl 4-[Phenyl (Propanoyl)Amino]-1-[2-(Pyridin-2-Yl)Ethyl]Piperidine-4-Carboxylate	N.A	N.A	**N**	**N**	**N**	**N**	**Y**
**147**	Methyl 4-[Phenyl (Propanoyl)Amino]-1-[2-(Thiophen-3-Yl)Ethyl]Piperidine-4-Carboxylate		Methyl 4-[Phenyl (Propanoyl)Amino]-1-[2-(Thiophen-3-Yl)Ethyl]Piperid	N.A	N.A	**N**	**N**	**N**	**N**	**Y**

			ine-4-<br>Carboxylate							
**148**	N-(2-Fluorophenyl)-N-[1-(2-Hydroxy-2-Phenylethyl)-3-Methylpiperidin-4-Yl]Propanamide		N-(2-Fluorophenyl)-N-[1-(2-hydroxy-2-phenylethyl)-3-methylpiperidin-4-yl]propanamide	N.A	N.A	**N**	**N**	**N**	**N**	**Y**
**149**	N-(2-Fluorophenyl)-N-[1-(2-Phenylethyl)-4-(1,3-Thiazol-2-Yl)Piperidin-4-Yl]Propanamide		N-(2-Fluorophenyl)-N-[1-(2-phenylethyl)-4-(1,3-thiazol-2-yl)piperidin-4-yl]propanamide	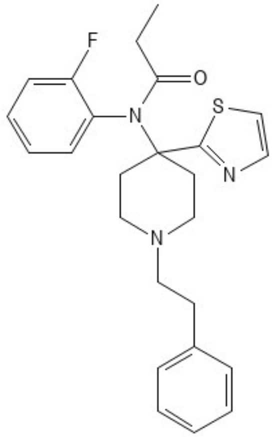	$C_{25}H_{28}FN_3OS$	**N**	**N**	**N**	**N**	**Y**
**150**	N-(2-Fluorophenyl)-N-[1-(2-Phenylethyl)-4-(Pyridin-2-Yl)Piperidin-4-Yl]Propanamide		N-(2-Fluorophenyl)-N-[1-(2-phenylethyl)-4-(pyridin-2-	N.A	N.A	**N**	**N**	**N**	**N**	**Y**

			yl)piperidin-4-yl]propanamide							
**151**	N-(2-Fluorophenyl)-N-[4-Phenyl-1-(2-Phenylethyl)Piperidin-4-Yl]Propanamide		N-(2-Fluorophenyl)-N-[4-phenyl-1-(2-phenylethyl)piperidin-4-yl]propanamide	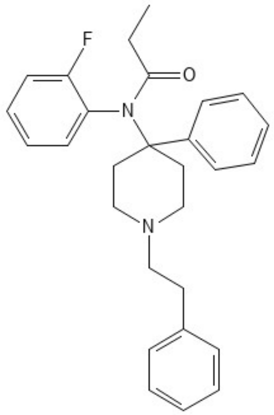	$C_{28}H_{31}FN_2O$	**N**	**N**	**N**	**N**	**Y**
**152**	N-(2-Fluorophenyl)-N-{1-[2-(1h-Pyrazol-1-Yl)Ethyl]-4-(Pyridin-2-Yl)Piperidin-4-Yl}Propanamide		N-(2-Fluorophenyl)-N-{1-[2-(1H-pyrazol-1-yl)ethyl]-4-(pyridin-2-yl)piperidin-4-yl}propanamide	N.A	N.A	**N**	**N**	**N**	**N**	**Y**

**153**	N-(2-Fluorophenyl)-N-{1-[2-(4-Methyl-1,3-Thiazol-5-Yl)Ethyl]-4-Phenylpiperidin-4-Yl}Propanamide		N-(2-Fluorophenyl)-N-{1-[2-(4-Methyl-1,3-Thiazol-5-Yl)Ethyl]-4-Phenylpiperidin-4-Yl}Propanamide	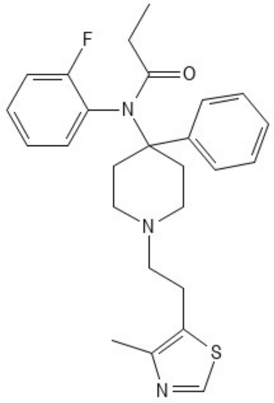	$C_{26}H_{30}FN_3OS$	**N**	**N**	**N**	**N**	**Y**
**154**	N-(2-Fluorophenyl)-N-{4-(4-Methyl-1,3-Thiazol-2-Yl)-1-[2-(1h-Pyrazol-1-Yl)Ethyl]Piperidin-4-Yl}Propanamide		N-(2-Fluorophenyl)-N-{4-(4-Methyl-1,3-Thiazol-2-Yl)-1-[2- (1h-Pyrazol-1-Yl)Ethyl]Piperidin-4-Yl}Propanamide	N.A	N.A	**N**	**N**	**N**	**N**	**Y**

**155**	N-(2-Fluorophenyl)-N-{4-(4-Methyl-1,3-Thiazol-2-Yl)-1-[2-(4-Methyl-1,3-Thiazol-5-Yl)Ethyl]Piperidin-4-Yl}Propanamide		N-(2-Fluorophenyl)-N-{4-(4-Methyl-1,3-Thiazol-2-Yl)-1-[2- (4-Methyl-1,3-Thiazol-5-Yl)Ethyl]Piperidin-4-Yl}Propanamide	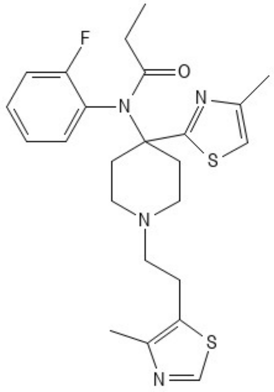	$C_{24}H_{29}FN_4OS_2$	**N**	**N**	**N**	**N**	**Y**
**156**	N-(2-Fluorophenyl)-N-{4-(4-Methyl-1,3-Thiazol-2-Yl)-1-[2-(Thiophen-3-Yl)Ethyl]Piperidin-4-Yl}Propanamide		N-(2-Fluorophenyl)-N-{4-(4-Methyl-1,3-Thiazol-2-Yl)-1-[2- (Thiophen-3-Yl)Ethyl]Piperidin-4-Yl}Propanamide	N.A	N.A	**N**	**N**	**N**	**N**	**Y**
**157**	N-(2-Fluorophenyl)-N-{4-Phenyl-1-[2-(1h-Pyrazol-1-Yl)Ethyl]Piperidin-4-Yl}Propanamide		N-(2-Fluorophenyl)-N-{4-Phenyl-1-[2-(1h-Pyrazol-1-Yl)Ethyl]Piperidin-4-	N.A	N.A	**N**	**N**	**N**	**N**	**Y**

			Yl}Propanamide							
**158**	N-(2-Fluorophenyl)-N-{4-Phenyl-1-[2-(Thiophen-2-Yl)Ethyl]Piperidin-4-Yl}Propanamide		N-(2-Fluorophenyl)-N-{4-Phenyl-1-[2-(Thiophen-2-Yl)Ethyl]Piperidin-4-Yl}Propanamide	N.A	N.A	**N**	**N**	**N**	**N**	**Y**
**159**	N-(2-Fluorophenyl)-N-{4-Phenyl-1-[2-(Thiophen-3-Yl)Ethyl]Piperidin-4-Yl}Propanamide		N-(2-Fluorophenyl)-N-{4-Phenyl-1-[2-(Thiophen-3-Yl)Ethyl]Piperidin-4-Yl}Propanamide	N.A	N.A	**N**	**N**	**N**	**N**	**Y**
**160**	N-(3-Fluorophenyl)-N-[1-(2-Hydroxy-2-Phenylethyl)-3-Methylpiperidin-4-Yl]Propanamide		N-(3-Fluorophenyl)-N-[1-(2-Hydroxy-2-Phenylethyl)-3-Methylpiperidin-4-Yl]Propanamide	N.A	N.A	**N**	**N**	**N**	**N**	**Y**

**161**	N-(4-Fluorophenyl)-N-[1-(2-Hydroxy-2-Phenylethyl)-3-Methylpiperidin-4-Yl]Propanamide		N-(4-Fluorophenyl)-N-[1-(2-Hydroxy-2-Phenylethyl)-3-Methylpiperidin-4-Yl]Propanamide	N.A	N.A	**N**	**N**	**N**	**N**	**Y**
**162**	N-[1-(2-Cyclopropyl-2-Hydroxyethyl)-3-Methylpiperidin-4-Yl]-N-Phenylpropanamide		N-[1-(2-Cyclopropyl-2-Hydroxyethyl)-3-Methylpiperidin-4-Yl]-N-Phenylpropanamide	N.A	N.A	**N**	**N**	**N**	**N**	**Y**
**163**	N-[1-(2-Hydroxy-2-Phenylethyl)-3-Methylpiperidin-4-Yl]-2-Methoxy-N-Phenylacetamide		N-[1-(2-Hydroxy-2-Phenylethyl)-3-Methylpiperidin-4-Yl]-2-Methoxy-N-Phenylacetamide	N.A	N.A	**N**	**N**	**N**	**N**	**Y**
**164**	N-[1-(2-Hydroxy-2-Phenylethyl)-3-Methylpiperidin-4-Yl]-N-(3-		N-[1-(2-Hydroxy-2-Phenylethyl)-3-Methylpiperidin	N.A	N.A	**N**	**N**	**N**	**N**	**Y**

	Methoxyphenyl)Propanamide		-4-Yl]-N-(3-Methoxyphenyl) Propanamide							
**165**	N-[1-(2-Hydroxy-2-Phenylethyl)-3-Methylpiperidin-4-Yl]-N-(Pyridin-2-Yl)Propanamide		N-[1-(2-Hydroxy-2-Phenylethyl)-3-Methylpiperidin-4-Yl]-N-(Pyridin-2-Yl)Propanamide	N.A	N.A	**N**	**N**	**N**	**N**	**Y**
**166**	N-[1-(2-Hydroxy-2-Phenylethyl)-3-Methylpiperidin-4-Yl]-N-(Pyridin-3-Yl)Propanamide		N-[1-(2-Hydroxy-2-Phenylethyl)-3-Methylpiperidin-4-Yl]-N-(Pyridin-3-Yl)Propanamide	N.A	N.A	**N**	**N**	**N**	**N**	**Y**
**167**	N-[1-(2-Hydroxy-2-Phenylethyl)-3-Methylpiperidin-4-Yl]-N-Phenylfuran-2-Carboxamide		N-[1-(2-Hydroxy-2-Phenylethyl)-3-Methylpiperidin-4-Yl]-N-Phenylfuran-2-Carboxamide	N.A	N.A	**N**	**N**	**N**	**N**	**Y**
**168**	N-[1-(2-Hydroxy-2-Phenylethyl)-3-Methylpiperidin-4-Yl]-		N-[1-(2-Hydroxy-2-Phenylethyl)-3-	N.A	N.A	**N**	**N**	**N**	**N**	**Y**

	N-Phenylfuran-3-Carboxamide		Methylpiperidin-4-Yl]-N-Phenylfuran-3-Carboxamide							
**169**	N-[1-(2-Hydroxy-2-Phenylethyl)-3-Methylpiperidin-4-Yl]-N-Phenylthiophene-2-Carboxamide		N-[1-(2-Hydroxy-2-Phenylethyl)-3-Methylpiperidin-4-Yl]-N-Phenylthiophene-2-Carboxamide	N.A	N.A	**N**	**N**	**N**	**N**	**Y**
**170**	N-[1-(2-Hydroxy-2-Phenylethyl)-3-Methylpiperidin-4-Yl]-N-Phenylthiophene-3-Carboxamide		N-[1-(2-Hydroxy-2-Phenylethyl)-3-Methylpiperidin-4-Yl]-N-Phenylthiophene-3-Carboxamide	N.A	N.A	**N**	**N**	**N**	**N**	**Y**
**171**	N-[1-(2-Hydroxy-2-Phenylethyl)-4-(Methoxymethyl)-3-Methylpiperidin-4-Yl]-N-Phenylpropanamide		N-[1-(2-Hydroxy-2-Phenylethyl)-4-(Methoxymethyl)-3-Methylpiperidin-4-Yl]-N-Phenylpropanamide	N.A	N.A	**N**	**N**	**N**	**N**	**Y**

**172**	N-[4-(4-Methyl-1,3-Thiazol-2-Yl)-1- (2-Phenylethyl)Piperidin-4-Yl]-N-Phenylpropanamide		N-[4-(4-Methyl-1,3-Thiazol-2-Yl)-1- (2-Phenylethyl)Piperidin-4-Yl]-N-Phenylpropanamide	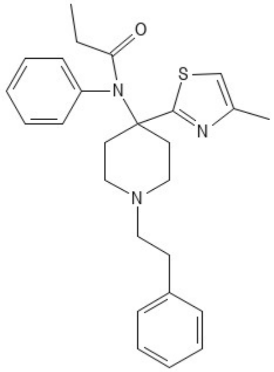	$C_{26}H_{31}N_3OS$	**N**	**N**	**N**	**N**	**Y**
**173**	N-{1-[(2r,3r)-3-Hydroxy-1,2,3,4-Tetrahydronaphthalen-2-Yl]-3-Methylpiperidin-4-Yl}-N-Phenylpropanamide		N-{1-[(2r,3r)-3-Hydroxy-1,2,3,4-Tetrahydronaphthalen-2-Yl]-3-Methylpiperidin-4-Yl}-N-Phenylpropanamide	N.A	N.A	**N**	**N**	**N**	**N**	**Y**
**174**	N-{1-[2-(3,5-Dimethyl-1h-Pyrazol-1-Yl)Ethyl]-4-Phenylpiperidin-4-Yl}-N- (2-Fluorophenyl)Propanamide		N-{1-[2-(3,5-Dimethyl-1h-Pyrazol-1-Yl)Ethyl]-4-Phenylpiperidin-4-Yl}-N- (2-Fluorophenyl)Propanamide	N.A	N.A	**N**	**N**	**N**	**N**	**Y**

**175**	N-{1-[2-(4-Ethyl-5-Oxo-4,5-Dihydro-1h-Tetrazol-1-Yl)Ethyl]-4- (1,3-Thiazol-2-Yl)Piperidin-4-Yl}-N-(2-Fluorophenyl)Propanamide		N-{1-[2-(4-Ethyl-5-Oxo-4,5-Dihydro-1h-Tetrazol-1-Yl)Ethyl]-4-(1,3-Thiazol-2-Yl)Piperidin-4-Yl}-N- (2-Fluorophenyl)Propanamide	N.A	N.A	**N**	**N**	**N**	**N**	**Y**
**176**	N-{1-[2-(Furan-2-Yl)-2-Hydroxyethyl]-4-(Methoxymethyl)Piperidin-4-Yl}-N-Phenylpropanamide		N-{1-[2-(Furan-2-Yl)-2-Hydroxyethyl]-4-(Methoxymethyl)Piperidin-4-Yl}-N-Phenylpropanamide	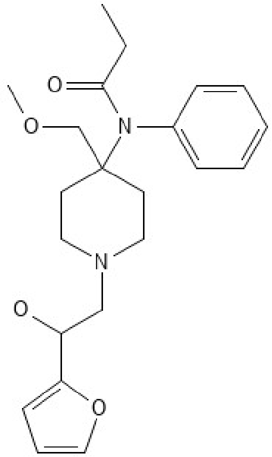	$C_{22}H_{30}N_2O_4$	**N**	**N**	**N**	**N**	**Y**
**177**	N-{1-[2-Hydroxy-2-(1-Methyl-1h-Pyrrol-2-Yl)Ethyl]-3-Methylpiperidin-4-		N-{1-[2-Hydroxy-2-(1-Methyl-1h-Pyrrol-2-Yl)Ethyl]-3-Methylpiperidin	N.A	N.A	**N**	**N**	**N**	**N**	**Y**

	Yl}-N-Phenylpropanamide		-4-Yl}-N-Phenylpropanamide							
**178**	N-{1-[2-Hydroxy-2-(Pyridin-3-Yl)Ethyl]-3-Methylpiperidin-4-Yl}-N-Phenylpropanamide		N-{1-[2-Hydroxy-2-(Pyridin-3-Yl)Ethyl]-3-Methylpiperidin-4-Yl}-N-Phenylpropanamide	N.A	N.A	**N**	**N**	**N**	**N**	**Y**
**179**	N-{1-[2-Hydroxy-2-(Pyridin-4-Yl)Ethyl]-3-Methylpiperidin-4-Yl}-N-Phenylpropanamide		N-{1-[2-Hydroxy-2-(Pyridin-4-Yl)Ethyl]-3-Methylpiperidin-4-Yl}-N-Phenylpropanamide	N.A	N.A	**N**	**N**	**N**	**N**	**Y**
**180**	N-{1-[2-Hydroxy-2-(Thiophen-2-Yl)Ethyl]-3-Methylpiperidin-4-Yl}-N-(3-Methoxyphenyl)Propanamide		N-{1-[2-Hydroxy-2-(Thiophen-2-Yl)Ethyl]-3-Methylpiperidin-4-Yl}-N-(3-Methoxyphenyl)Propanamide	N.A	N.A	**N**	**N**	**N**	**N**	**Y**

**181**	N-Adamantyl-Fentanyl		N-(Adamantan-1-yl)-N-[1-(2-phenylethyl)piperidin-4-yl]propanamide	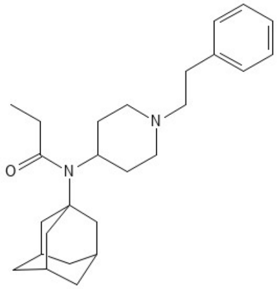	$C_{26}H_{38}N_2O$	**N**	**N**	**N**	**N**	**Y**
**182**	N-Benzoxazolyl-Fentanyl		N-(1,3-Benzoxazol-6-yl)-N-[1-(2-phenylethyl)piperidin-4-yl]propanamide	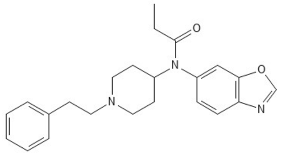	$C_{23}H_{27}N_3O_2$	**N**	**N**	**N**	**N**	**Y**
**183**	N-Benzyl-Acetylfentanyl		N-(1-Benzylpiperidin-4-yl)-N-phenylacetamide	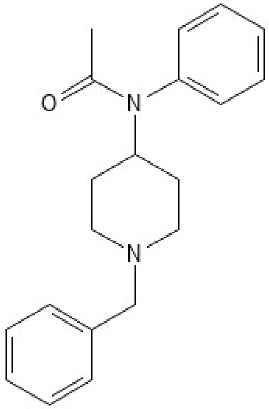	$C_{20}H_{24}N_2O$	**N**	**N**	**N**	**N**	**Y**

**184**	N-Benzyl-Butyrylfentanyl		N-(1-Benzylpiperidin-4-yl)-N-phenylbutanamide	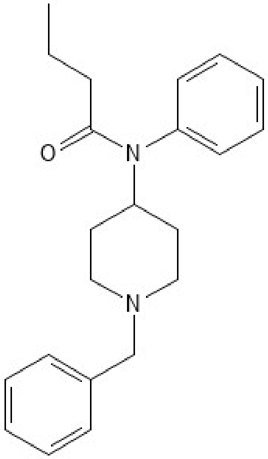	$C_{22}H_{28}N_2O$	**N**	**N**	**N**	**N**	**Y**
**185**	N-Benzylcarfentanil	Benzylcarfentanil	Methyl 1-benzyl-4-[phenyl(propanoyl)amino]piperidine-4-carboxylate	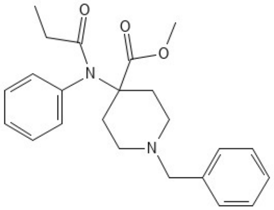	$C_{23}H_{28}N_2O_3$	**N**	**N**	**N**	**N**	**Y**

**186**	N-Benzyl-P-Fluoro-Isobutyrylfentanyl		1-(1-Benzylpiperidin-4-yl)-1-(4-fluorophenyl)-3-methylbutan-2-one	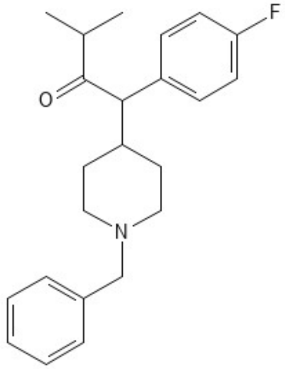	$C_{23}H_{28}FNO$	**N**	**N**	**N**	**N**	**Y**
**187**	N-Furanylethylfentanyl		N-{1-[2-(Furan-2-yl)ethyl]piperidin-4-yl}-N-phenylpropanamide	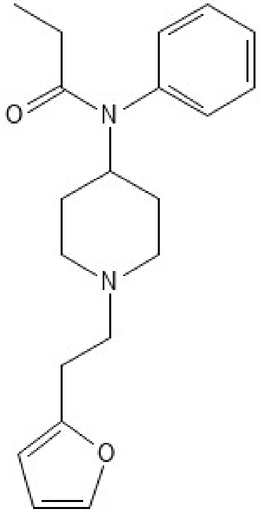	$C_{20}H_{26}N_2O_2$	**N**	**N**	**N**	**N**	**Y**

**188**	N-Methyl-Acetylfentanyl	N-Methyl-acetyl-norfentanyl	N-(1-Methylpiperidin-4-yl)-N-phenylacetamide	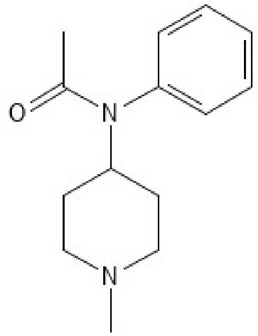	$C_{14}H_{20}N_2O$	**N**	**N**	**N**	**N**	**Y**
**189**	N-Methyl-Butyrylfentanyl		N-(1-Methylpiperidin-4-yl)-N-phenylbutanamide	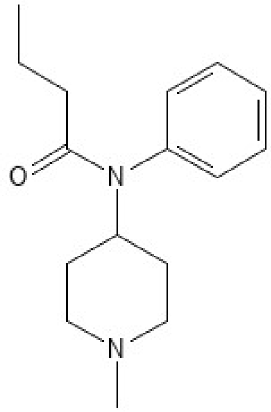	$C_{16}H_{24}N_2O$	**N**	**N**	**N**	**N**	**Y**

**190**	N-Phenyl-N-[1-(2-Phenylethyl)-4- (1,3-Thiazol-2-Yl)Piperidin-4-Yl]Propanamide		N-Phenyl-N-[1-(2-phenylethyl)-4-(1,3-thiazol-2-yl)piperidin-4-yl]propanamide	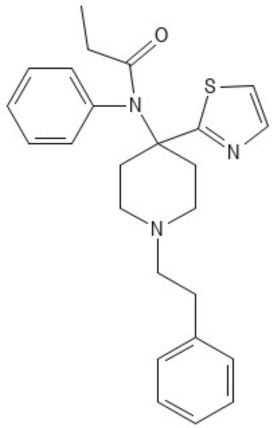	$C_{25}H_{29}N_3OS$	**N**	**N**	**N**	**N**	**Y**
**191**	N-Phenyl-N-{4-Phenyl-1-[2-(1h-Pyrazol-1-Yl)Ethyl]Piperidin-4-Yl}Propanamide		N-Phenyl-N-{4-Phenyl-1-[2-(1h-Pyrazol-1-Yl)Ethyl]Piperidin-4-Yl}Propanamide	N.A	N.A	**N**	**N**	**N**	**N**	**Y**
**192**	N-Phenyl-N-{4-Phenyl-1-[2-(Pyridin-2-Yl)Ethyl]Piperidin-4-Yl}Propanamide		N-Phenyl-N-{4-Phenyl-1-[2-(Pyridin-2-Yl)Ethyl]Piperidin-4-Yl}Propanamide	N.A	N.A	**N**	**N**	**N**	**N**	**Y**
**193**	N-Phenyl-N-{4-Phenyl-1-[2-		N-Phenyl-N-{4-Phenyl-1-[2-	N.A	N.A	**N**	**N**	**N**	**N**	**Y**

	(Thiophen-2-Yl)Ethyl]Piperidin-4-Yl}Propanamide		(Thiophen-2-Yl)Ethyl]Piperidin-4-Yl}Propanamide							
**194**	N-Phenyl-N-{4-Phenyl-1-[2-(Thiophen-3-Yl)Ethyl]Piperidin-4-Yl}Propanamide		N-Phenyl-N-{4-Phenyl-1-[2-(Thiophen-3-Yl)Ethyl]Piperidin-4-Yl}Propanamide	N.A	N.A	**N**	**N**	**N**	**N**	**Y**
**195**	N-Quinolinyl-Fentanyl		N-[1-(2-Phenylethyl)piperidin-4-yl]-N-(quinolin-8-yl)propanamide	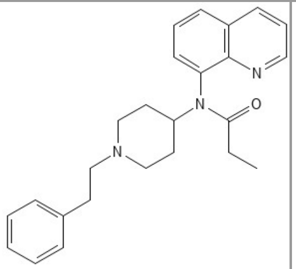	$C_{25}H_{29}N_3O$	**N**	**N**	**N**	**N**	**Y**

**196**	O-Fluoro-Despropionoylfentanyl	2′-Fluoro-despropionoylfentanyl	N-(2-Fluorophenyl)-1-(2-phenylethyl)piperidin-4-amine	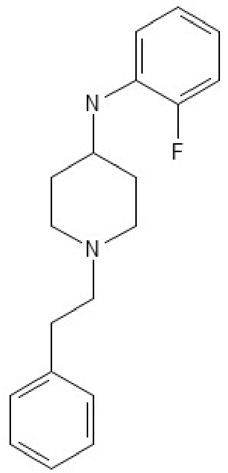	$C_{19}H_{23}FN_2$	**N**	**N**	**N**	**N**	**Y**
**197**	O-Methyl-Benzoylfentanyl	2′-Methyl-benzoylfentanyl	N-(2-Methylphenyl)-N-[1-(2-phenylethyl)piperidin-4-yl]benzamide	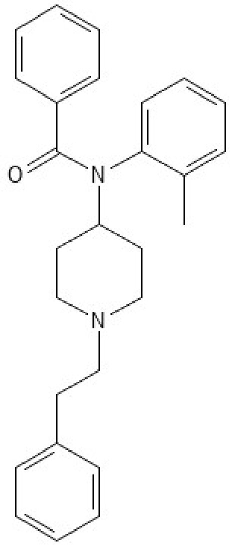	$C_{27}H_{30}N_2O$	**N**	**N**	**N**	**N**	**Y**

**198**	O-Methyl-Cyclopropylfentanyl	2′-Methyl-cyclopropylfentanyl	N-(2-Methylphenyl)-N-[1-(2-phenylethyl)piperidin-4-yl]cyclopropanecarboxamide	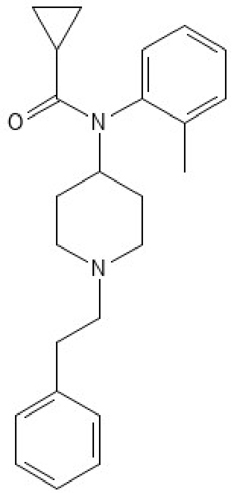	$C_{24}H_{30}N_2O$	**Y**	**N**	**N**	**N**	**N**
**199**	O-Methylfentanyl	2′-Methylfentanyl	N-(2-Methylphenyl)-N-[1-(2-phenylethyl)piperidin-4-yl]propanamide	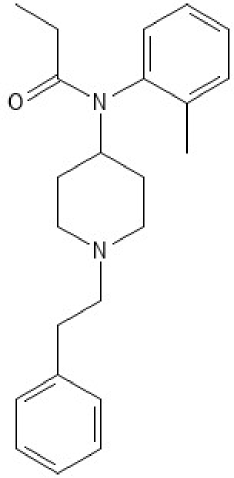	$C_{23}H_{30}N_2O$	**Y**	**Y**	**N**	**N**	**N**

**200**	Ortho-Methyl Furanyl Fentanyl	o-Methyl-furanylfentanyl; 2′-Methyl-furanylfentanyl; 2′-Me-Fu-F	N-(2-Methylphenyl)-N-[1-(2-phenylethyl)piperidin-4-yl]furan-2-carboxamide	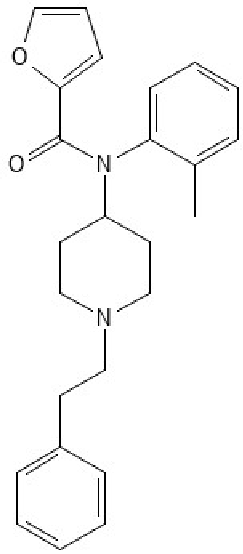	$C_{25}H_{28}N_2O_2$	**Y**	**N**	**N**	**N**	**N**

**201**	Para-Chlorofuranylfentanyl	p-Chloro-furanylfentanyl; 4′-Chloro-furanylfentanyl; p-Cl-Fu-F	N-(4-Chlorophenyl)-N-[1-(2-phenylethyl)piperidin-4-yl]furan-2-carboxamide	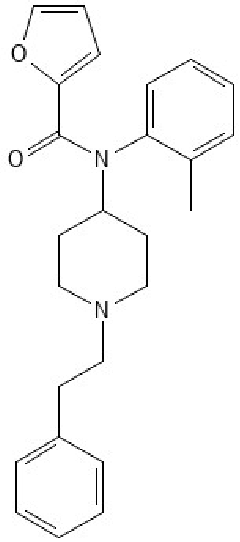	$C_{24}H_{25}ClN_2O_2$	**Y**	**N**	**N**	**N**	**N**
**202**	P-Bromofentanyl	p-Bromofentanyl; 4′-Bromofentanyl	N-(4-Bromophenyl)-N-[1-(2-phenylethyl)piperidin-4-yl]propanamide	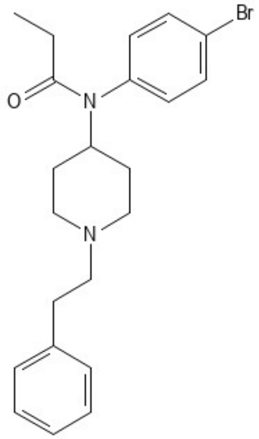	$C_{22}H_{27}BrN_2O$	**Y**	**N**	**N**	**N**	**N**

**203**	P-Fluoro-Crotonylfentanyl	p-Fluoro-crotonylfentanyl; 4′-Fluoro-crotonylfentanyl	(2E)-N-(4-Fluorophenyl)-N-[1-(2-phenylethyl)piperidin-4-yl]but-2-enamide	N.A	N.A	**N**	**N**	**N**	**N**	**Y**
**204**	P-Fluoro-Cyclopentylfentanyl	p-Fluoro-cyclopentylfentanyl; 4′-Fluoro-cyclopentylfentanyl	N-(4-Fluorophenyl)-N-[1-(2-phenylethyl)piperidin-4-yl]cyclopentanecarboxamide	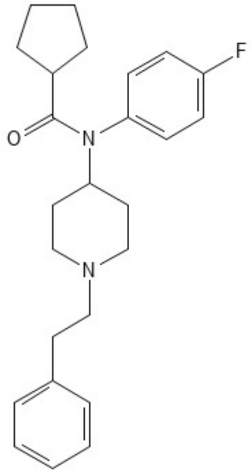	$C_{25}H_{31}FN_2O$	**N**	**N**	**N**	**N**	**Y**

**205**	P-Fluoro-Cyclopropylfentanyl	p-Fluoro-cyclopropylfentanyl; 4′-Fluoro-cyclopropylfentanyl	N-(4-Fluorophenyl)-N-[1-(2-phenylethyl)piperidin-4-yl]cyclopropanecarboxamide	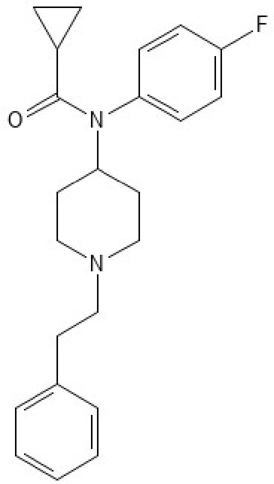	$C_{23}H_{27}FN_2O$	**N**	**N**	**N**	**N**	**Y**
**206**	P-Fluoro-Furan-3-Ylfentanyl	p-Fluoro-furan-3-ylfentanyl; 4′-Fluoro-furan-3-ylfentanyl	N-(4-Fluorophenyl)-N-[1-(2-phenylethyl)piperidin-4-yl]furan-3-carboxamide	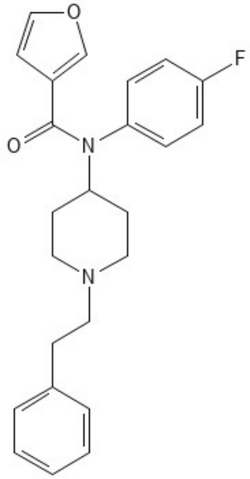	$C_{24}H_{25}FN_2O_2$	**N**	**N**	**N**	**N**	**Y**

**207**	P-Fluoro-Furanylethylfentanyl	p-Fluoro-furanylethylfentanyl; 4′-Fluoro-furanylethylfentanyl	1-(4-fluorophenyl)-1-[1-[2-(furan-2-yl)ethyl]piperidin-4-yl]butan-2-one	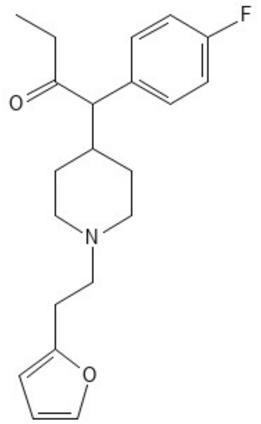	$C_{21}H_{26}FNO_2$	**N**	**N**	**N**	**N**	**Y**
**208**	P-Fluoro-Furanylfentanyl	p-Fluoro-furanylfentanyl; 4′-Fluoro-furanylfentanyl; p-F-Fu-F	N-(4-Fluorophenyl)-N-[1-(2-phenylethyl)piperidin-4-yl]furan-2-carboxamide	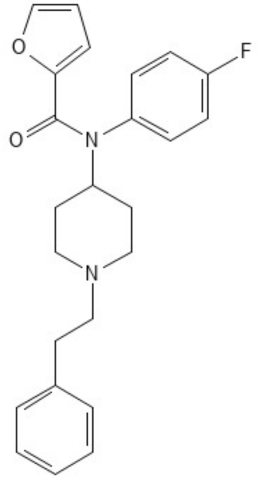	$C_{24}H_{25}FN_2O_2$	**Y**	**N**	**N**	**N**	**N**

**209**	P-Fluoro-Furanylremifentanil	p-Fluoro-furanylremifentanil	Methyl 4-[(4-fluorophenyl)(furan-2-carbonyl)amino]-1-(3-methoxy-3-oxopropyl)piperidine-4-carboxylate	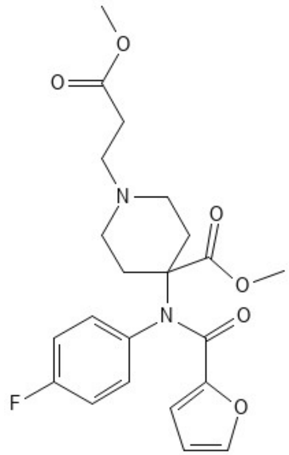	$C_{22}H_{25}FN_2O_6$	**N**	**N**	**N**	**N**	**Y**
**210**	P-Fluoro-Methoxyacetylfentanyl	p-Fluoro-methoxyacetylfentanyl; 4′-Fluoro-methoxyacetylfentanyl	N-(4-Fluorophenyl)-2-methoxy-N-[1-(2-phenylethyl)piperidin-4-yl]acetamide	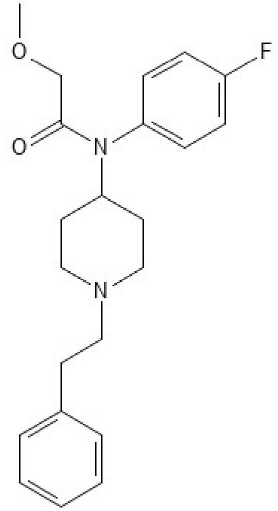	$C_{22}H_{27}FN_2O_2$	**N**	**N**	**N**	**N**	**Y**

**211**	P-Fluoro-Tetrahydrofuranylfentanyl	p-Fluoro-tetrahydrofuranylfentanyl; 4′-Fluoro-tetrahydrofuranylfentanyl	*N*-(4-fluorophenyl)-*N*-[1-(2-phenylethyl)piperidin-4-yl]oxolane-2-carboxamide -4-yl]oxolane-2-carboxamide	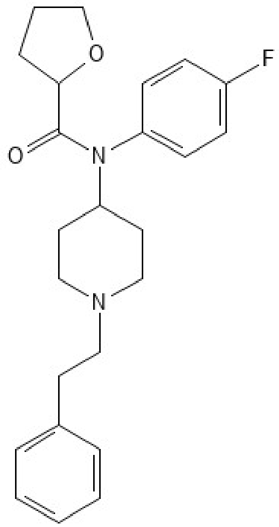	$C_{24}H_{29}FN_2O_2$	**N**	**N**	**N**	**N**	**Y**
**212**	P-Fluoro-Thiofentanyl	p-Fluoro-thiofentanyl; 4′-Fluoro-thiofentanyl	N-(4-Fluorophenyl)-N-{1-[2-(thiophen-2-yl)ethyl]piperidin-4-yl}propanamide	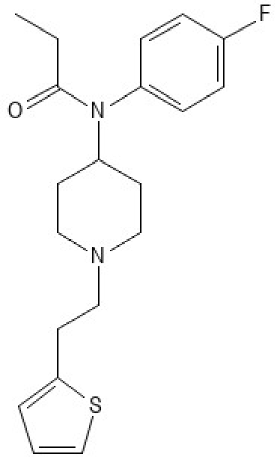	$C_{20}H_{25}FN_2OS$	**N**	**N**	**N**	**N**	**Y**

**213**	Pharaohfentanyl		N-[1-(2-Hydroxy-2-phenylethyl)-4-methylpiperidin-4-yl]-N-phenylpropanamide	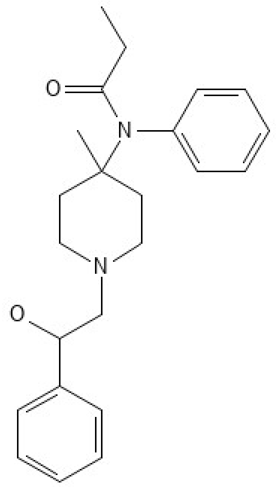	$C_{23}H_{30}N_2O_2$	**N**	**N**	**N**	**N**	**Y**
**214**	Phenaridine	2,5-Dimethylfentanyl	N-[2,5-Dimethyl-1-(2-phenylethyl)piperidin-4-yl]-N-phenylpropanamide	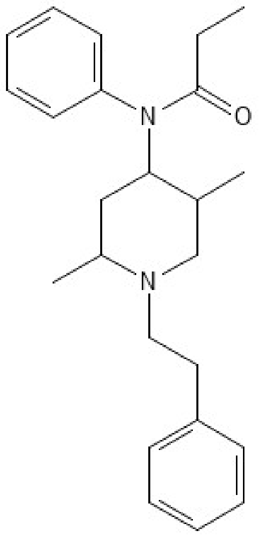	$C_{24}H_{32}N_2O$	**N**	**N**	**N**	**N**	**Y**

**215**	Phenoxyethyl-Norfentanyl	POE-F	N-[1-(2-Phenoxyethyl)piperidin-4-yl]-N-phenylpropanamide	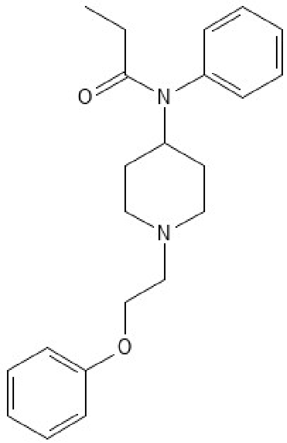	$C_{22}H_{28}N_2O_2$	**N**	**N**	**N**	**N**	**Y**
**216**	Phenylacetylfentanyl		N,2-Diphenyl-N-[1-(2-phenylethyl)piperidin-4-yl]acetamide	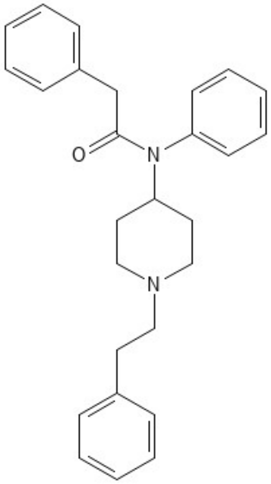	$C_{27}H_{30}N_2O$	**N**	**N**	**N**	**N**	**Y**

**217**	Phenylpropyl-Norfentanyl	PP-F	N-Phenyl-N-[1-(3-phenylpropyl)piperidin-4-yl]propanamide	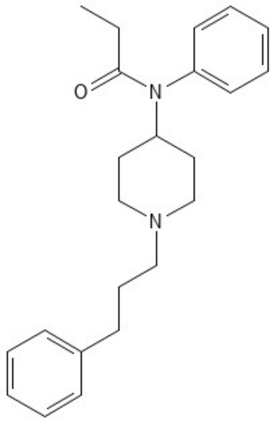	$C_{23}H_{30}N_2O$	**N**	**N**	**N**	**N**	**Y**
**218**	P-Iodofentanyl	p-Iodofentanyl; 4′-Iodofentanyl	N-(4-Iodophenyl)-N-[1-(2-phenylethyl)piperidin-4-yl]propanamide	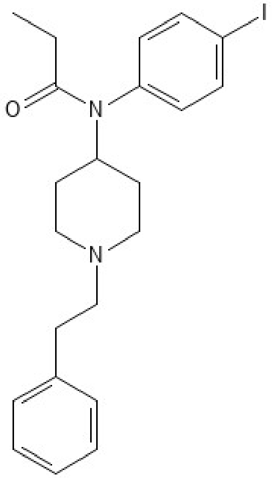	$C_{22}H_{27}IN_2O$	**N**	**N**	**N**	**N**	**Y**

**219**	Pivaloylfentanyl		2,2-Dimethyl-N-phenyl-N-[1-(2-phenylethyl)piperidin-4-yl]propanamide	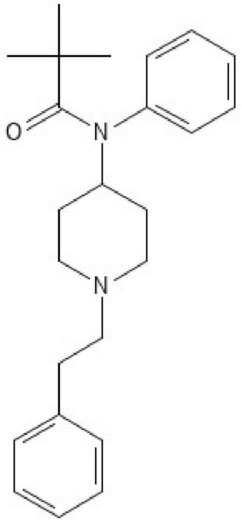	$C_{24}H_{32}N_2O$	**N**	**N**	**N**	**N**	**Y**
**220**	P-Methoxy-Acetylfentanyl	p-Methoxy-acetylfentanyl; 4′-Methoxy-acetylfentanyl; 4′-MeO-AF	N-(4-Methoxyphenyl)-N-[1-(2-phenylethyl)piperidin-4-yl]acetamide	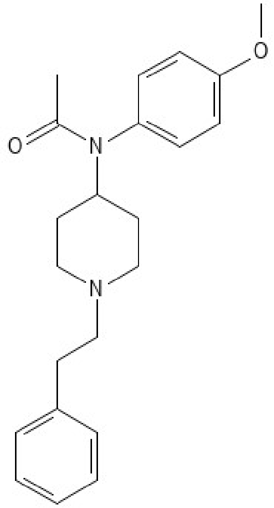	$C_{22}H_{28}N_2O_2$	**N**	**N**	**N**	**N**	**Y**

**221**	P-Methoxy-Butyrylfentanyl	p-Methoxy-butyrylfentanyl; 4′-Methoxy-butyrylfentanyl; p-MeO-BF	N-(4-Methoxyphenyl)-N-[1-(2-phenylethyl)piperidin-4-yl]butanamide	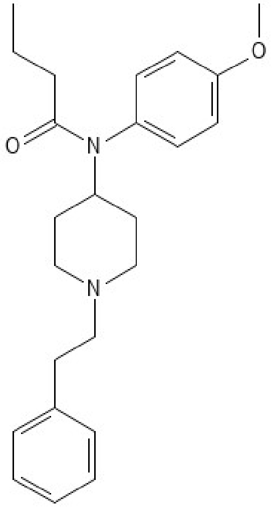	$C_{24}H_{32}N_2O_2$	**Y**	**N**	**N**	**N**	**N**
**222**	P-Methoxy-Methoxyacetylfentanyl	p-Methoxy-methoxyacetylfentanyl; 4′-Methoxy-methoxyacetylfentanyl	2-Methoxy-N-(4-methoxyphenyl)-N-[1-(2-phenylethyl)piperidin-4-yl]acetamide	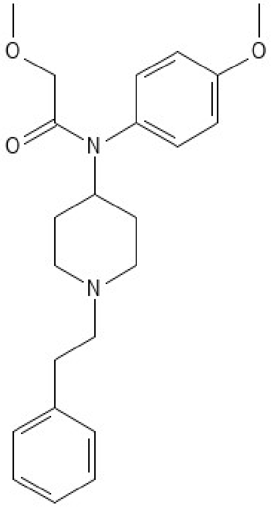	$C_{23}H_{30}N_2O_3$	**N**	**N**	**N**	**N**	**Y**

**223**	P-Methoxy-Tetrahydrofuranylfentanyl	p-Methoxy-tetrahydrofuranylfentanyl; 4′-Methoxy-tetrahydrofuranylfentanyl	N-(4-Methoxyphenyl)-N-[1-(2-phenylethyl)piperidin-4-yl]oxolane-2-carboxamide	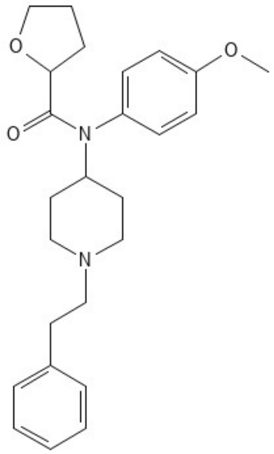	$C_{25}H_{32}N_2O_3$	**N**	**N**	**N**	**N**	**Y**
**224**	P-Methoxy-Valerylfentanyl	4′-Methoxy-tetrahydrofuranylfentanyl	*N*-(4-methoxyphenyl)-*N*-[1-(2-phenylethyl)piperidin-4-yl]pentanamide	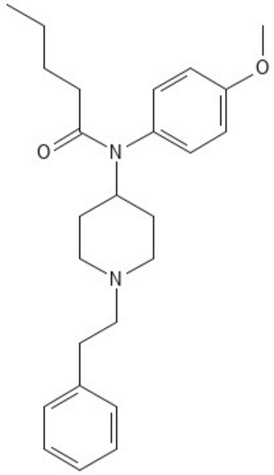	$C_{25}H_{34}N_2O_2$	**N**	**N**	**N**	**N**	**Y**

**225**	P-Methyl-Acetylfentanyl	4′-Methyl-acetylfentanyl	N-(4-Methylphenyl)-N-[1-(2-phenylethyl)piperidin-4-yl]acetamide	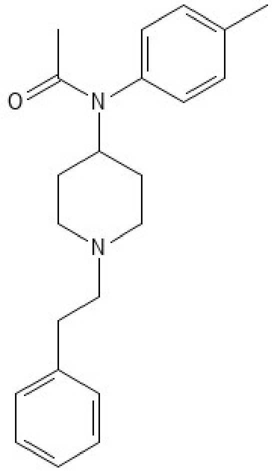	$C_{22}H_{28}N_2O$	**Y**	**N**	**N**	**N**	**N**
**226**	P-Methyl-Cyclopropylfentanyl	4′-Methyl-cyclopropylfentanyl	N-(4-Methylphenyl)-N-[1-(2-phenylethyl)piperidin-4-yl]cyclopropanecarboxamide	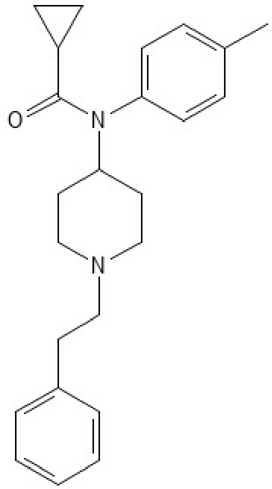	$C_{24}H_{30}N_2O$	**Y**	**Y**	**N**	**N**	**N**

**227**	Propylnorfentanyl		N-Phenyl-N-(1-propylpiperidin-4-yl)propanamide	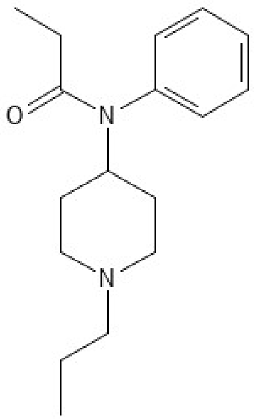	$C_{17}H_{26}N_2O$	**N**	**N**	**N**	**N**	**Y**
**228**	Psicofentanil	EAZ-91-05	1-azabicyclo[2.2.2]octan-3-yl 1-[2-(1*H*-indol-3-yl)ethyl]-4-(*N*-propanoylanilino)piperidine-4-carboxylate	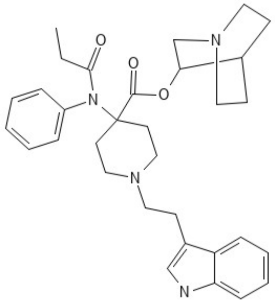	$C_{32}H_{40}N_4O_3$	**N**	**N**	**N**	**N**	**Y**

**229**	P-TFM-Fentanyl	4′-TFM-fentanyl	N-[1-(2-Phenylethyl)piperidin-4-yl]-N-[4-(trifluoromethyl)phenyl]propanamide	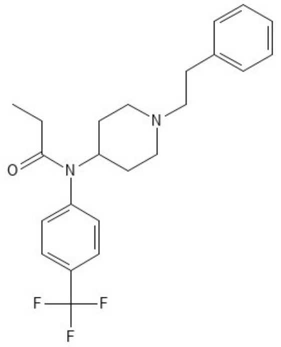	$C_{23}H_{27}F_3N_2O$	**N**	**N**	**N**	**N**	**Y**
**230**	Remifentanil	Remifentanyl; Ultiva	methyl 1-(3-methoxy-3-oxopropyl)-4-(N-propanoylanilino)piperidine-4-carboxylate	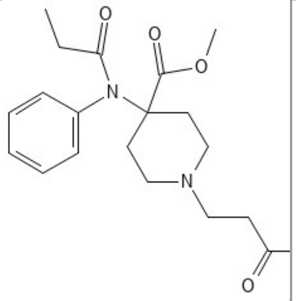	$C_{20}H_{28}N_2O_5$	**N**	**N**	**Y**	**N**	**N**
**231**	Remifentanil Bis Ethyl Ester		Ethyl 1-(3-ethoxy-3-oxopropyl)-4-[phenyl(propanoyl)amino]piperidine-4-carboxylate	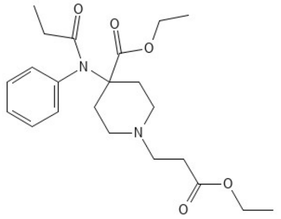	$C_{22}H_{32}N_2O_5$	**N**	**N**	**N**	**N**	**Y**

**232**	Sufentanil	Dsuvia; Sufenta	N-[4-(methoxymethyl)-1-(2-thiophen-2-ylethyl)piperidin-4-yl]-N-phenylpropanamide	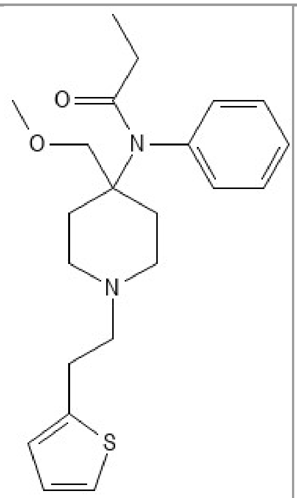	$C_{22}H_{30}N_2O_2S$	**N**	**N**	**Y**	**N**	**N**
**233**	Tetrahydrofuran-Fentanyl	tetrahydrofuranfentanyl; THF-F (hydrochloride)	*N*-phenyl-*N*-[1-(2-phenylethyl)piperidin-4-yl]oxolane-2-carboxamide	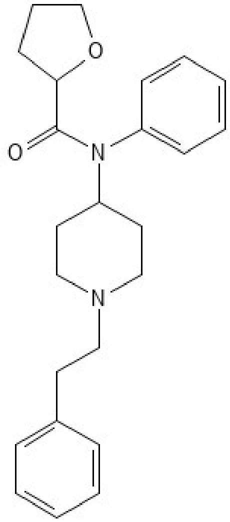	$C_{24}H_{30}N_2O_2$	**N**	**Y**	**Y**	**N**	**N**

**234**	Thiafentanil	A-3080	methyl 4-(*N*-(2-methoxyacetyl)anilino)-1-(2-thiophen-2-ylethyl)piperidine-4-carboxylate	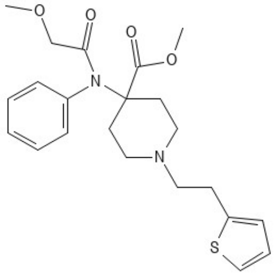	$C_{22}H_{28}N_2O_4S$	**N**	**N**	**N**	**N**	**Y**
**235**	Thiophenoylfentanyl		N-Phenyl-N-[1-(2-phenylethyl)piperidin-4-yl]thiophene-2-carboxamide	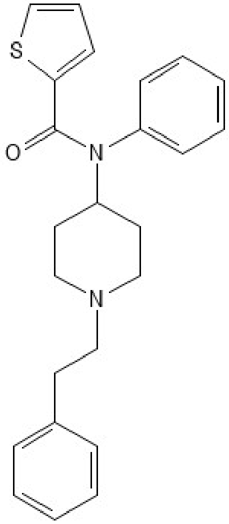	$C_{24}H_{26}N_2OS$	**N**	**N**	**N**	**N**	**Y**

**236**	W-18*		4-chloro-*N*-[1-[2-(4-nitrophenyl)ethyl]piperidin-2-ylidene]benzene sulfonamide	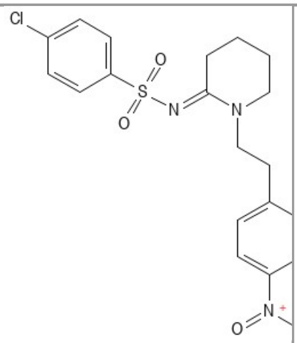	$C_{19}H_{20}ClN_3O_4S$	**Y**	**N**	**N**	**N**	**N**
**237**	A-Methyl-Isobutyrylfentanyl	α-Methyl-isobutyrylfentanyl	2-Methyl-N-phenyl-N-[1-(1-phenylpropan-2-yl)piperidin-4-yl]propanamide	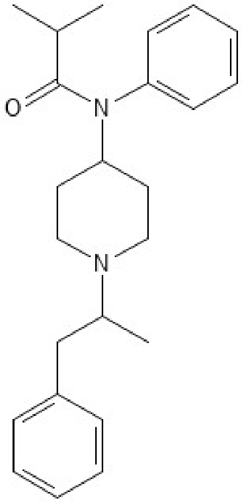	$C_{24}H_{32}N_2O$	**N**	**N**	**N**	**N**	**Y**

**Potentially a fentanyl analogue with minimal or no opioid activity (Huang et al., 2017).*

Table S1 – NPSfinder® fentanyl analogues; IUPAC names and comparisons between the different databases. NPSfinder® crawling in 2018 Table 1 (Arillotta et al., 2020, Table 1)

N	Molecule denomination in NPS*finder*®	Other Names	Chemical Name (IUPAC)	Chemical Structure	Molecular Formula	UNODC EWA on NPS (May 2025)	CFSRE (NPS Discovery) (December 2024)	INCB Yellow list (July 2024)	INCB Green list (January 2025)	Unique to NPS*finder*® database
**1**	Acetyldihydrocodeine	Dihydrocodeine 6-acetate; Dihydrothebacone	[(4R,4aR,7S,7aR,12bS)-9-methoxy-3-methyl-2,4,4a,5,6,7,7a,13-octahydro-1H-4,12-methanobenzofuro[3,2-e]isoquinolin-7-yl] acetate	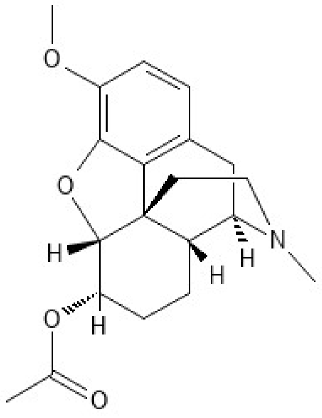	$C_{20}H_{25}NO_4$	**N**	**N**	**Y**	**N**	**N**

**2**	Anileridine	Leritine	ethyl 1-[2-(4-aminophenyl)ethyl]-4-phenylpiperidine-4-carboxylate	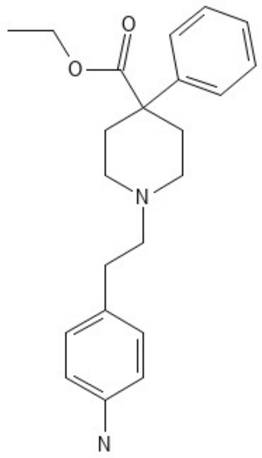	$C_{22}H_{28}N_2O_2$	**N**	**N**	**Y**	**N**	**N**
**3**	Bezitramide	Benzitramide; Burgodin	4-[4-(2-oxo-3-propanoylbenzimidazol-1-yl)piperidin-1-yl]-2,2-diphenylbutanenitrile	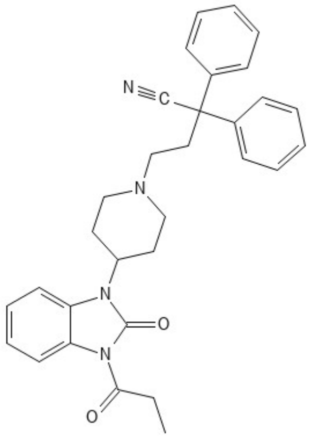	$C_{31}H_{32}N_4O_2$	**N**	**N**	**Y**	**N**	**N**

**4**	Buprenorphine	Subutex	(1S,2S,6R,14R,15R,16R)-5-(cyclopropylmethyl)-16-[(2S)-2-hydroxy-3,3-dimethylbutan-2-yl]-15-methoxy-13-oxa-5-azahexacyclo[13.2.2.12,8.01,6.02,14.012,20]icosa-8(20),9,11-trien-11-ol	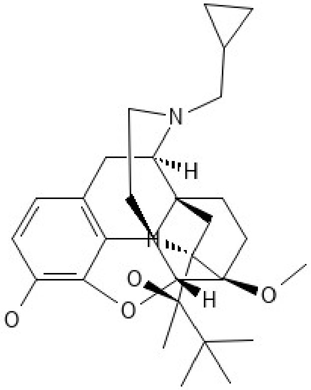	$C_{29}H_{41}NO_4$	**N**	**N**	**Y**	**N**	**N**
**5**	Butorphanol	Butorfanol; Stadol	(1S,9R,10S)-17-(cyclobutylmethyl)-17-azatetracyclo[7.5.3.01,10.02,7]heptadeca-2(7),3,5-triene-4,10-diol	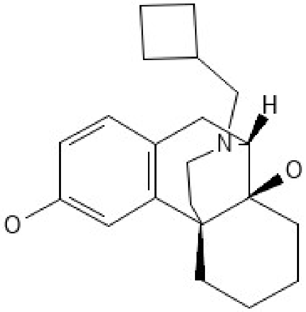	$C_{21}H_{29}NO_2$	**N**	**N**	**N**	**N**	**Y**

**6**	Codeine	3-Methoxy morphine	(4R,4aR,7S,7aR,12bS)-9-methoxy-3-methyl-2,4,4a,7,7a,13-hexahydro-1H-4,12-methanobenzofuro[3,2-e]isoquinolin-7-ol	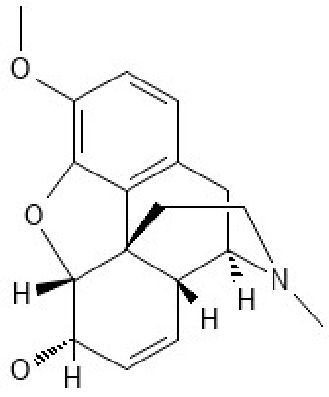	$C_{18}H_{21}NO_3$	**N**	**N**	**Y**	**N**	**N**
**7**	Co-Proxamol	Distalgesic; Coproxamol	[(2*S*,3*R*)-4-(dimethylamino)-3-methyl-1,2-diphenylbutan-2-yl] propanoate;*N*-(4-hydroxyphenyl)acetamide	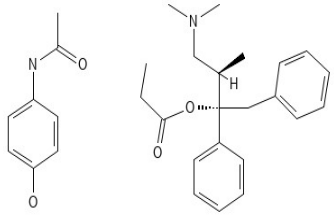	$C_{30}H_{38}N_2O_4$	**N**	**N**	**N**	**N**	**Y**
**8**	Dextromethorphan	d-Methorphan; DXM; DM	(1S,9S,10S)-4-methoxy-17-methyl-17-azatetracyclo[7.5.3.01,10.02,7]heptadeca-2(7),3,5-triene	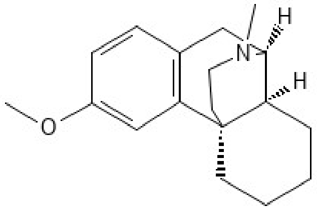	$C_{18}H_{25}NO$	**N**	**N**	**N**	**N**	**Y**

**9**	Dextromoramide	Palfium; Palphium; Jetrium; Dimorlin	(3S)-3-methyl-4-morpholin-4-yl-2,2-diphenyl-1-pyrrolidin-1-ylbutan-1-one	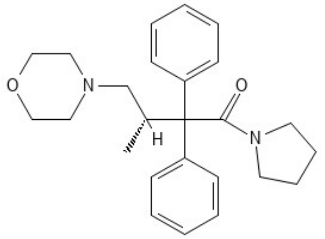	$C_{25}H_{32}N_2O_2$	**N**	**N**	**Y**	**N**	**N**
**10**	Dextropropoxyphene	propoxyphene; Darvon	[(2S,3R)-4-(dimethylamino)-3-methyl-1,2-diphenylbutan-2-yl] propanoate	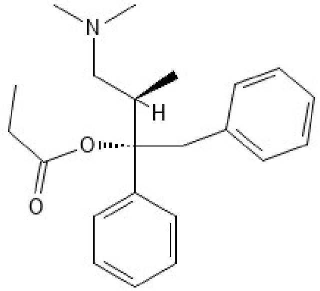	$C_{22}H_{29}NO_2$	**N**	**N**	**Y**	**N**	**N**
**11**	Diamorphine	Diamorphine hydrochloride (approved); diamorphine; diacetylmorphine; morphine diacetate; dope; H; smack;	[(4R,4aR,7S,7aR,12bS)-9-acetyloxy-3-methyl-2,4,4a,7,7a,13-hexahydro-1H-4,12-methanobenzofuro[3,2-e]isoquinolin-7-yl] acetate	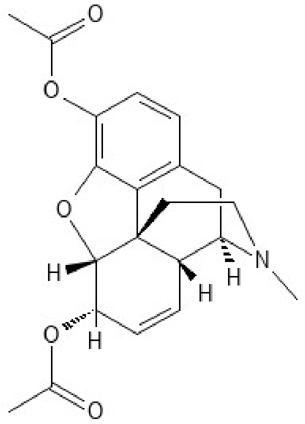	$C_{21}H_{23}NO_5$	**N**	**N**	**N**	**N**	**Y**

		junk; horse; brown								
**12**	Difenoxin	Motofen; R-15403	1-(3-cyano-3,3-diphenylpropyl)-4-phenylpiperidine-4-carboxylic acid	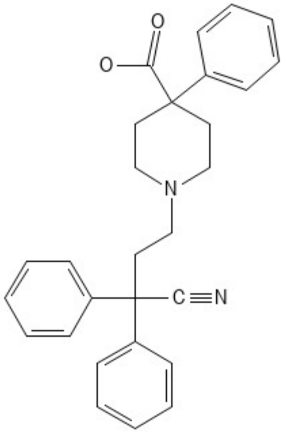	$C_{28}H_{28}N_2O_2$	**N**	**N**	**Y**	**N**	**N**
**13**	Dihydrocodeine	Hydrocodeine; 6-alpha-Hydrocodol	(4R,4aR,7S,7aR,12bS)-9-methoxy-3-methyl-2,4,4a,5,6,7,7a,13-octahydro-1H-4,12-methanobenzofuro[3,2-e]isoquinolin-7-ol	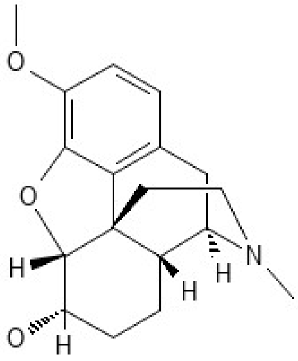	$C_{18}H_{23}NO_3$	**N**	**N**	**Y**	**N**	**N**

**14**	Diphenoxylate	R-1132	ethyl 1-(3-cyano-3,3-diphenylpropyl)-4-phenylpiperidine-4-carboxylate	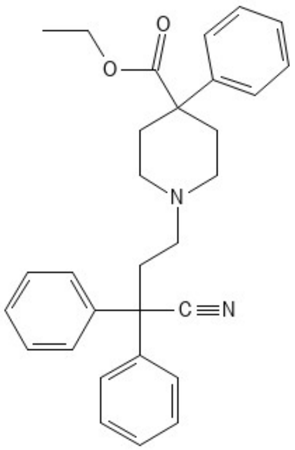	$C_{30}H_{32}N_2O_2$	**N**	**N**	**Y**	**N**	**N**
**15**	Eluxadoline	Viberzi; Truberzi	5-[[[(2S)-2-amino-3-(4-carbamoyl-2,6-dimethylphenyl) propanoyl]-[(1S)-1-(5-phenyl-1H-imidazol-2-yl)ethyl]amino] methyl]-2-methoxybenzoic acid	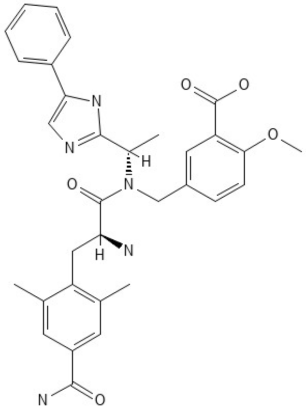	$C_{32}H_{35}N_5O_5$	**N**	**N**	**N**	**N**	**Y**

**16**	Ethylmorphine	codethyline; dionine; ethyl morphine	(4R,4aR,7S,7aR,12bS)-9-ethoxy-3-methyl-2,4,4a,7,7a,13-hexahydro-1H-4,12-methanobenzofuro[3,2-e]isoquinolin-7-ol	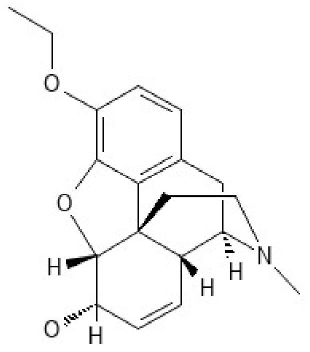	$C_{19}H_{23}NO_3$	**N**	**N**	**Y**	**N**	**N**
**17**	Hydrocodone	Dihydrocodeinone; Vicodin; Norco	(4R,4aR,7aR,12bS)-9-methoxy-3-methyl-1,2,4,4a,5,6,7a,13-octahydro-4,12-methanobenzofuro[3,2-e]isoquinolin-7-one	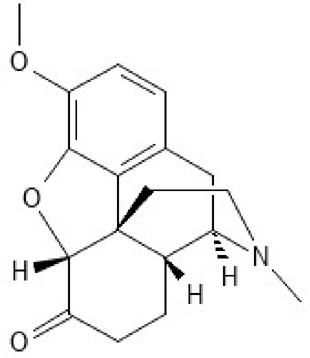	$C_{18}H_{21}NO_3$	**N**	**N**	**Y**	**N**	**N**

**18**	Hydromorphone	dihydromorphinone; Dilaudid	(4R,4aR,7aR,12bS)-9-hydroxy-3-methyl-1,2,4,4a,5,6,7a,13-octahydro-4,12-methanobenzofuro[3,2-e]isoquinolin-7-one	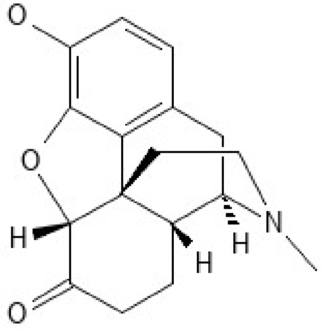	$C_{17}H_{19}NO_3$	**N**	**N**	**Y**	**N**	**N**
**19**	Ketobemidone	Cliradon; Cymidon; Ketogan; Ketorax	1-[4-(3-hydroxyphenyl)-1-methylpiperidin-4-yl]propan-1-one	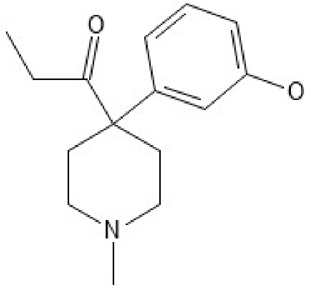	$C_{15}H_{21}NO_2$	**N**	**N**	**Y**	**N**	**N**
**20**	Levacetylmethadol	levomethadyl acetate; levo-α-acetylmethadol; LAAM; OrLAAM	[(3S,6S)-6-(dimethylamino)-4,4-diphenylheptan-3-yl] acetate	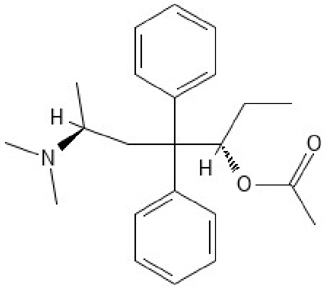	$C_{23}H_{31}NO_2$	**N**	**N**	**N**	**N**	**Y**

**21**	Loperamide	Imodium	4-[4-(4-chlorophenyl)-4-hydroxypiperidin-1-yl]-N,N-dimethyl-2,2-diphenylbutanamide	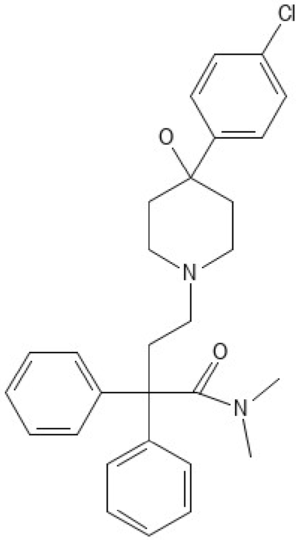	$C_{29}H_{33}ClN_2O_2$	**N**	**N**	**N**	**N**	**Y**
**22**	Methadone	Dolophine; Amidone; Biodone; Physeptone; Adanon; Diaminon; Ketalgin	6-(dimethylamino)-4,4-diphenylheptan-3-one	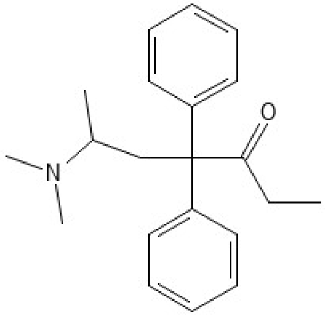	$C_{21}H_{27}NO$	**N**	**N**	**Y**	**N**	**N**

**23**	Morphine	Statex; MSContin; Oramorph; Sevredol	(4R,4aR,7S,7aR,12bS)-3-methyl-2,4,4a,7,7a,13-hexahydro-1H-4,12-methanobenzofuro[3,2-e]isoquinoline-7,9-diol	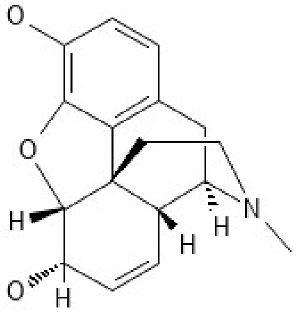	$C_{17}H_{19}NO_3$	**N**	**N**	**Y**	**N**	**N**
**24**	Nalbuphine	Nubain	(4R,4aS,7S,7aR,12bS)-3-(cyclobutylmethyl)-1,2,4,5,6,7,7a,13-octahydro-4,12-methanobenzofuro[3,2-e]isoquinoline-4a,7,9-triol	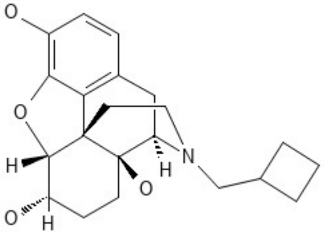	$C_{21}H_{27}NO_4$	**N**	**N**	**N**	**N**	**Y**
**25**	Nalmefene	Nalmetrene; Selincro	(4R,4aS,7aS,12bS)-3-(cyclopropylmethyl)-7-methylidene-2,4,5,6,7a,13-hexahydro-1H-4,12-methanobenzofuro[3,2-	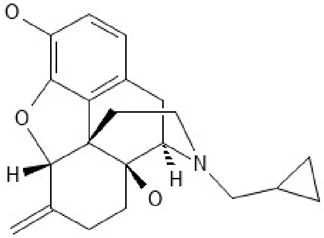	$C_{21}H_{25}NO_3$	**N**	**N**	**N**	**N**	**Y**

			e]isoquinoline-4a,9-diol							
**26**	Nalorphine	Allorphine; N-allylnormorphine; Lethidrone; Nalline	(4R,4aR,7S,7aR,12bS)-3-prop-2-enyl-2,4,4a,7,7a,13-hexahydro-1H-4,12-methanobenzofuro[3,2-e]isoquinoline-7,9-diol	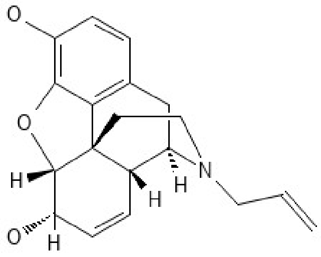	$C_{19}H_{21}NO_3$	**N**	**N**	**N**	**N**	**Y**
**27**	Naloxegol	PEGylated naloxol; Movantik; Moventig; NKTR-118	(4R,4aS,7S,7aR,12bS)-7-[2-[2-[2-[2-[2-[2-(2-methoxyethoxy)ethoxy]ethoxy]ethoxy]ethoxy]ethoxy]ethoxy]-3-prop-2-enyl-1,2,4,5,6,7,7a,13-octahydro-4,12-methanobenzofuro[3,2-e]isoquinoline-4a,9-diol	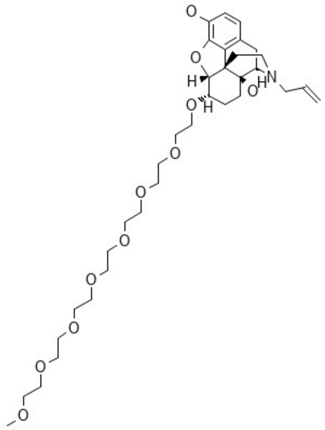	$C_{34}H_{53}NO_{11}$	**N**	**N**	**N**	**N**	**Y**

**28**	Naloxone	Narcan; Evzio	(4R,4aS,7aR,12bS)-4a,9-dihydroxy-3-prop-2-enyl-2,4,5,6,7a,13-hexahydro-1H-4,12-methanobenzofuro[3,2-e]isoquinolin-7-one	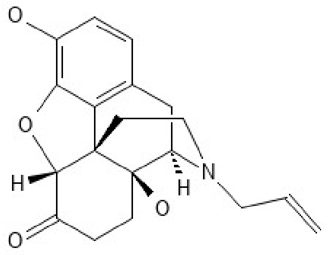	$C_{19}H_{21}NO_4$	**N**	**N**	**N**	**N**	**Y**
**29**	Naltrexone	ReVia; Vivitrol	(4R,4aS,7aR,12bS)-3-(cyclopropylmethyl)-4a,9-dihydroxy-2,4,5,6,7a,13-hexahydro-1H-4,12-methanobenzofuro[3,2-e]isoquinolin-7-one	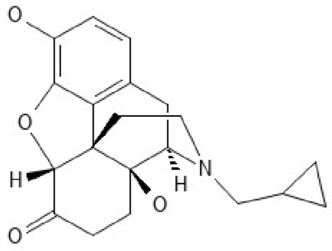	$C_{20}H_{23}NO_4$	**N**	**N**	**N**	**N**	**Y**

**30**	Nicomorphine	Vilan; Subellan; Gevilan; MorZet	[(4R,4aR,7S,7aR,12bS)-3-methyl-9-(pyridine-3-carbonyloxy)-2,4,4a,7,7a,13-hexahydro-1H-4,12-methanobenzofuro[3,2-e]isoquinolin-7-yl] pyridine-3-carboxylate	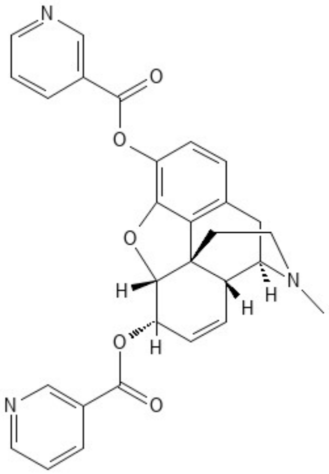	$C_{29}H_{25}N_3O_5$	**N**	**N**	**Y**	**N**	**N**
**31**	Normethadone	Phenyldimazone; desmethylmethadone; Cophylac; Dacartil; Eucopon; Mepidon; Noramidone; Normedon	6-(dimethylamino)-4,4-diphenylhexan-3-one	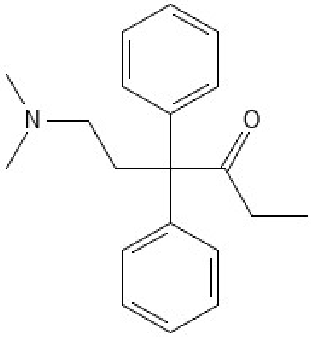	$C_{20}H_{25}NO$	**N**	**N**	**Y**	**N**	**N**

**32**	Noscapine	Narcotine; Nectodon; Nospen; Anarcotine; Opiane	(3S)-6,7-dimethoxy-3-[(5R)-4-methoxy-6-methyl-7,8-dihydro-5H-[1,3]dioxolo[4,5-g]isoquinolin-5-yl]-3H-2-benzofuran-1-one	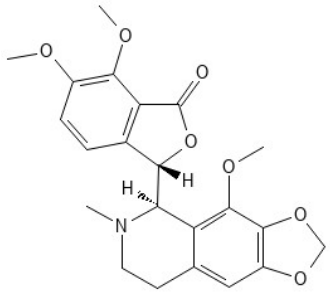	$C_{22}H_{23}NO_7$	**N**	**N**	**N**	**N**	**Y**
**33**	Opium	Lachryma papaveris; poppy tears	N.A	**N.A**	N.A	**N**	**N**	**Y**	**N**	**N**
**34**	Oxycodone	dihydrohydroxycodeinone; OxyContin; Eukodal; eucodal	(4R,4aS,7aR,12bS)-4a-hydroxy-9-methoxy-3-methyl-2,4,5,6,7a,13-hexahydro-1H-4,12-methanobenzofuro[3,2-e]isoquinolin-7-one	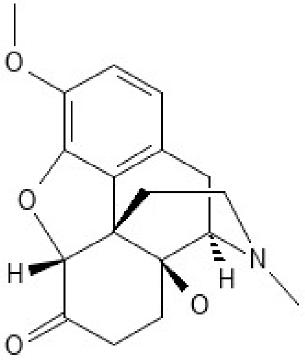	$C_{18}H_{21}NO_4$	**N**	**N**	**Y**	**N**	**N**

**35**	Pentazocine	Talwin	(1R,9R,13R)-1,13-dimethyl-10-(3-methylbut-2-enyl)-10-azatricyclo[7.3.1.02,7]trideca-2(7),3,5-trien-4-ol	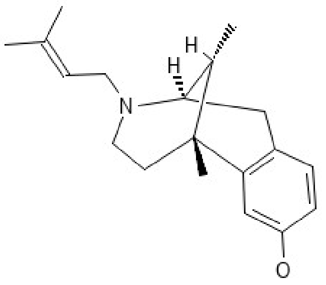	$C_{19}H_{27}NO$	**N**	**N**	**N**	**Y**	**N**
**36**	Pethidine	meperidine; Demerol	ethyl 1-methyl-4-phenylpiperidine-4-carboxylate	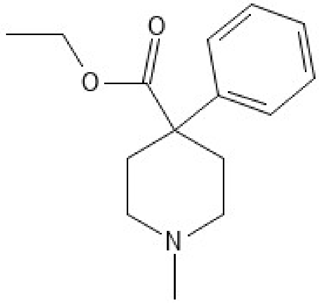	$C_{15}H_{21}NO_2$	**N**	**N**	**Y**	**N**	**N**
**37**	Phenazocine	Prinadol; Narphen	1,13-dimethyl-10-(2-phenylethyl)-10-azatricyclo[7.3.1.02,7]trideca-2(7),3,5-trien-4-ol	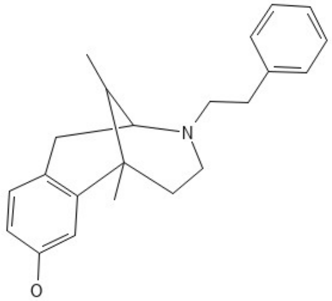	$C_{22}H_{27}NO$	**N**	**N**	**Y**	**N**	**N**

**38**	Phenoperidine	Operidine ; Lealgin	ethyl 1-(3-hydroxy-3-phenylpropyl)-4-phenylpiperidine-4-carboxylate	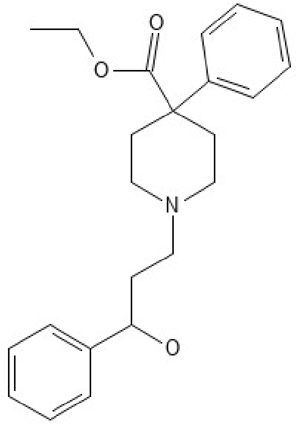	$C_{23}H_{29}NO_3$	**N**	**N**	**Y**	**N**	**N**
**39**	Pholcodine	Logicin	(4R,4aR,7S,7aR,12bS)-3-methyl-9-(2-morpholin-4-ylethoxy)-2,4,4a,7,7a,13-hexahydro-1H-4,12-methanobenzofuro[3,2-e]isoquinolin-7-ol	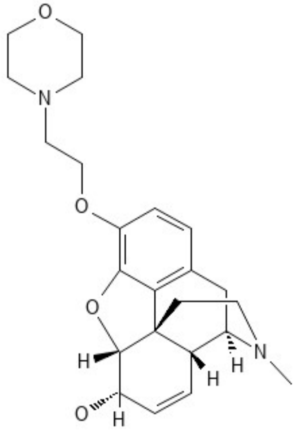	$C_{23}H_{30}N_2O_4$	**N**	**N**	**Y**	**N**	**N**

**40**	Piritramide	R-3365; Dipidolor ; Piridolan; Pirium	1-(3-cyano-3,3-diphenylpropyl)-4-piperidin-1-ylpiperidine-4-carboxamide	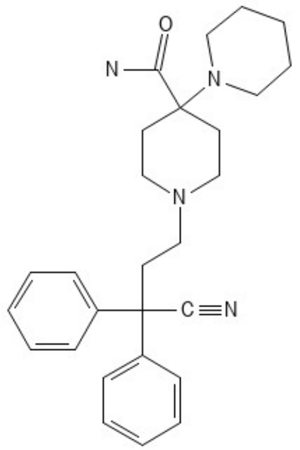	$C_{27}H_{34}N_4O$	**N**	**N**	**Y**	**N**	**N**
**41**	Tapentadol	Nucynta; Palexia; Yantil; Tapenta; Tapal	3-[(2R,3R)-1-(dimethylamino)-2-methylpentan-3-yl]phenol	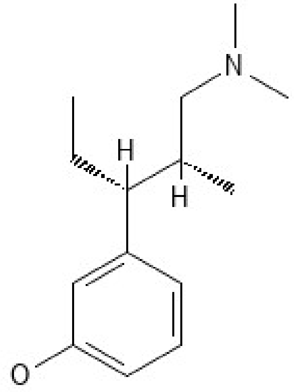	$C_{14}H_{23}NO$	**N**	**N**	**N**	**N**	**Y**

**42**	Thebacon	tebacon; Acedicon; Diacodin dihydrocodeinone enol acetate	[(4*R*,4*aR*,7*aR*,12*bS*)-9-methoxy-3-methyl-2,4,4*a*,5,7*a*,13-hexahydro-1*H*-4,12-methanobenzofuro[3,2-e]isoquinolin-7-yl] acetate	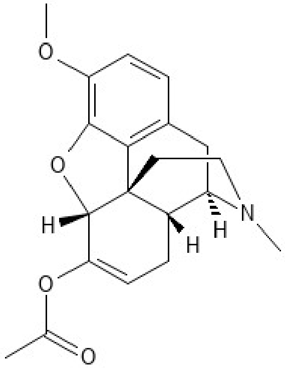	$C_{20}H_{23}NO_4$	**N**	**N**	**Y**	**N**	**N**
**43**	Tilidine	tilidate; Tilidin; Valoron; Valtran; dextilidine	ethyl (1S,2R)-2-(dimethylamino)-1-phenylcyclohex-3-ene-1-carboxylate	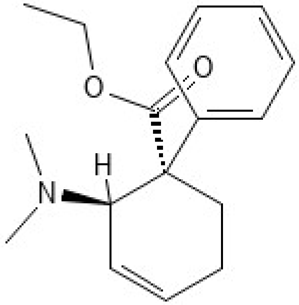	$C_{17}H_{23}NO_2$	**N**	**N**	**Y**	**N**	**N**
**44**	Tramadol	Ultram; Zytram	(1R,2R)-2-[(dimethylamino)methyl]-1-(3-methoxyphenyl)cyclohexan-1-ol	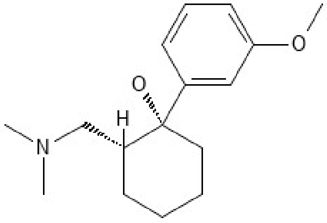	$C_{16}H_{25}NO_2$	**N**	**N**	**N**	**N**	**Y**

Table S2A – NPSfinder® ATC/DDD non fentanyl analogues (ATC/DDD opioids) and comparison between the different databases. NPSfinder® crawling in 2018 (Arillotta et al., 2020, Table 2A).

N	Molecule denomination in NPS*finder*®	Other Names	Chemical Name (IUPAC)	Chemical Structure	Molecular Formula	UNODC EWA on NPS (May 2025)	CFSRE (NPS Discovery) (December 2024)	INCB Yellow list (July 2024)	INCB Green list (January 2025)	Unique to NPS*finder*® database
**1**	Concentrate Of Poppy Straw	Opium straw; mowed opium straw; crushed poppy capsule; poppy chaff; poppy husk	N.A	N.A	N.A	**N**	**N**	**Y**	**N**	**N**
**2**	Granulate opium		N.A	N.A	N.A	**N**	**N**	**N**	**N**	**Y**
**3**	Kratom	Mitragyna speciosa; Ketum; Kakuam; Ithang; Thom;	N.A	N.A	N.A	**Y**	**N**	**N**	**N**	**N**

		krath`m (Thai); ketum; kratum								
**4**	Medicinal opium		N.A	N.A	N.A	**N**	**N**	**N**	**N**	**Y**
**5**	Papaver somniferum	Opium; Plant of Joy; Mawseed ; Joy Plant; Pen Yan; Paregoric	N.A	N.A	N.A	**N**	**N**	**N**	**N**	**Y**
**6**	PST	Poppy-seed-tea; poppy-tea	N.A	N.A	N.A	**N**	**N**	**N**	**N**	**Y**
**7**	Salvia Divinorum	Ska Pastora; Shepherdess's Herb; ska Maria Pastora;	N.A	N.A	N.A	**Y**	**N**	**N**	**N**	**N**

		yerba de Maria; Sally-D; Ska María Pastora; Seer's Sage; diviners mint; diviners sage								
**8**	Tincture of opium	Laudanum; Paregoric	N.A	N.A	N.A	**N**	**N**	**N**	**N**	**Y**

Table S2B – NPSfinder® plants and derivatives and comparison between the different databases. NPSfinder® crawling in 2018. (Arillotta et al., 2020 , Table 2B)

N	Molecule denomination in NPS*finder*®	Other Names	Chemical Name (IUPAC)	Chemical Structure	Molecular Formula	UNODC EWA on NPS (May 2025)	CFSRE (NPS Discovery) (December 2024)	INCB Yellow list (July 2024)	INCB Green list (January 2025)	Unique to NPS*finder*® database
**1**	03-monoacetylmorphine oxalate		3-Monoacetylmorphine: [(4*R*,4*aR*,7*S*,7*aR*,12*bS*)-7-hydroxy-3-methyl-2,4,4*a*,7,7*a*,13-hexahydro-1*H*-4,12-methanobenzofuro[3,2-e]isoquinolin-9-yl] acetate	3-Monoacetylmorphine: 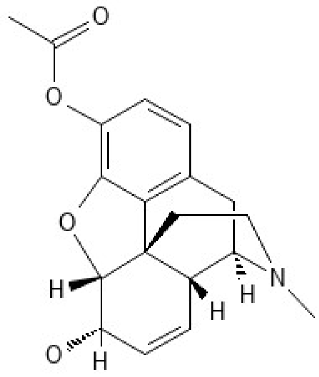	3-Monoacetylmorphine: $C_{19}H_{21}NO_4$	**N**	**N**	**N**	**N**	**Y**

**2**	14-hydroxymorphine	RAM-371	(4R,4aS,7S,7aR,12bS)-3-methyl-1,2,4,7,7a,13-hexahydro-4,12-methanobenzofuro[3,2-e]isoquinoline-4a,7,9-triol	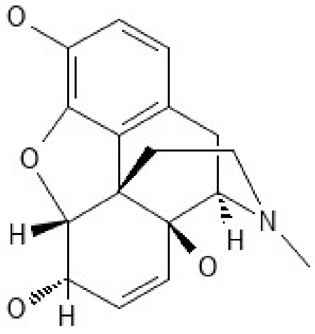	C17H19NO4	**N**	**N**	**N**	**N**	**Y**
**3**	2-ethyl-AP-237	2-methyl-bucinnazine	1-[2-methyl-4-(3-phenylprop-2-en-1-yl)piperazin-1-yl]butan-1-one	N.A	N.A	**N**	**N**	**N**	**N**	**Y**

**4**	3-(O-carboxymethyl)morphine		2-[[(4R,4aR,7S,7aR,12bS)-7-hydroxy-3-methyl-2,4,4a,7,7a,13-hexahydro-1H-4,12-methanobenzofuro[3,2-e]isoquinolin-9-yl]oxy]acetic acid	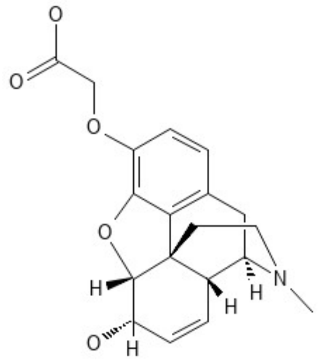	C19H21NO5	**N**	**N**	**N**	**N**	**Y**
**5**	3-benzylmorphine		(4R,4aR,7S,7aR,12bS)-9-benzyl-3-methyl-1,2,4,4a,7,7a,10,13-octahydro-4,12-methanobenzofuro[3,2-e]isoquinoline-7,9-diol	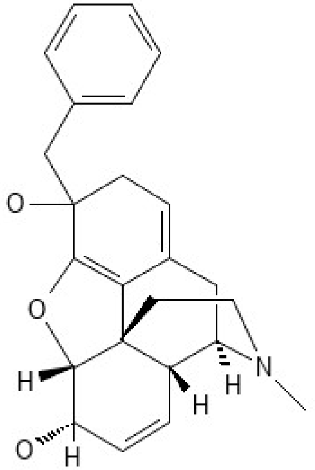	$C_{24}H_{27}NO_3$	**N**	**N**	**Y**	**N**	**N**

**6**	3-hydroxy-N-methylmorphinan	Dromoran; (+-)-3-Hydroxy-N-methylmorphinan = Racemorphan; (-)-3-Hydroxy-N-methylmorphinan = Levorphanol, levo-Dromoran (see prescription drugs)	(1*R*,9*R*,10*R*)-17-methyl-17-azatetracyclo[7.5.3.0^{1,10}.0^{2,7}]heptadeca-2(7),3,5-trien-4-ol	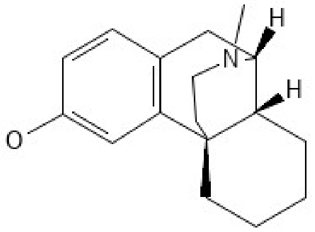	$C_{17}H_{23}NO$	**N**	**N**	**Y**	**N**	**N**

**7**	3-monoacetylmorphine	3-MAM; 3-acetylmorphine	[(4*R*,4*aR*,7*S*,7*aR*,12*bS*)-7-hydroxy-3-methyl-2,4,4*a*,7,7*a*,13-hexahydro-1*H*-4,12-methanobenzofuro[3,2-e]isoquinolin-9-yl] acetate	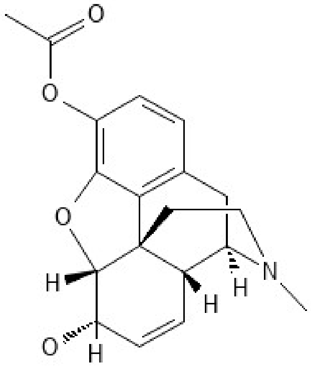	$C_{19}H_{21}NO_4$	**N**	**N**	**N**	**N**	**Y**
**8**	6-acetylmorphine hydrochloride		1-[(4R,4aR,7S,7aR,12bS)-7,9-dihydroxy-3-methyl-1,2,4,4a,7a,13-hexahydro-4,12-methanobenzofuro[3,2-e]isoquinolin-7-yl]ethanone; hydrochloride	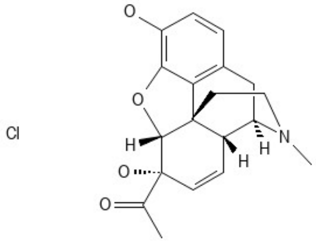	C19H22ClNO4	**N**	**N**	**N**	**N**	**Y**

**9**	6-methylenedihydrodesoxymorphine	6-MDDM; 6-Methylenedihydroepoxymorphine	(4R,4aR,7aS,12bS)-3-methyl-7-methylidene-1,2,4,4a,5,6,7a,13-octahydro-4,12-methanobenzofuro[3,2-e]isoquinolin-9-ol	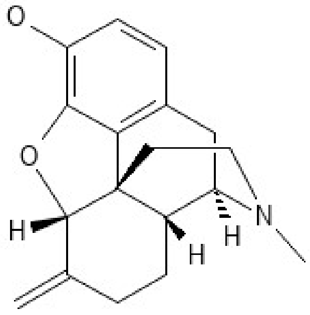	C18H21NO2	**N**	**N**	**N**	**N**	**Y**
**10**	6-monoacetylmorphine	6-MAM; 6-acetylmorphine; 6-AM	[(4R,4aR,7S,7aR,12bS)-9-hydroxy-3-methyl-2,4,4a,7,7a,13-hexahydro-1H-4,12-methanobenzofuro[3,2-e]isoquinolin-7-yl] acetate	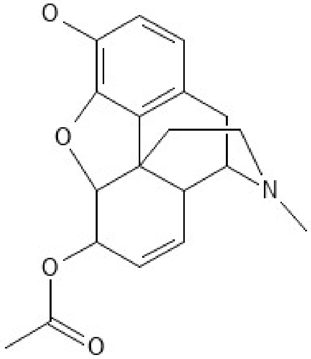	C19H21NO4	**N**	**N**	**N**	**N**	**Y**

**11**	6-nicotinoyldihydrocodeine	Nicodicodine; 6-Nicotinoyl dihydrocodeine	[(4R,4aR,7S,7aR,12bS)-9-methoxy-3-methyl-2,4,4a,5,6,7,7a,13-octahydro-1H-4,12-methanobenzofuro[3,2-e]isoquinolin-7-yl] pyridine-3-carboxylate	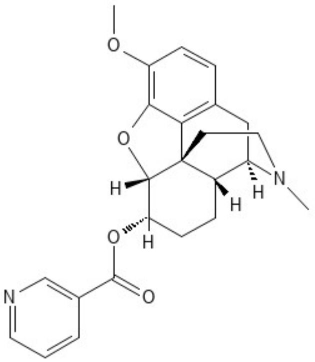	C24H26N2O4	**N**	**N**	**Y**	**N**	**N**
**12**	Acetorphine		[(1R,2S,6R,14R,15R,19R)-19-[(2R)-2-hydroxypentan-2-yl]-15-methoxy-5-methyl-13-oxa-5-azahexacyclo[13.2.2.12,8.01,6.02,14.012,20]icosa-8(20),9,11,16-tetraen-11-yl] acetate	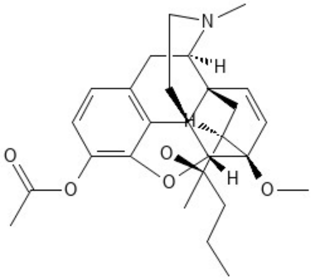	C27H35NO5	**N**	**N**	**Y**	**N**	**N**

**13**	Acetoxyketobemidone	O-Acetylketobemidone	3-(1-Methyl-4-propionyl-4-piperidinyl)phenyl acetate	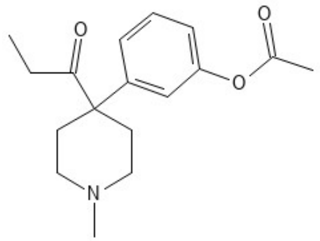	C17H23NO3	**N**	**N**	**N**	**N**	**Y**
**14**	Acetylmethadol	methadyl acetate	[6-(dimethylamino)-4,4-diphenylheptan-3-yl] acetate	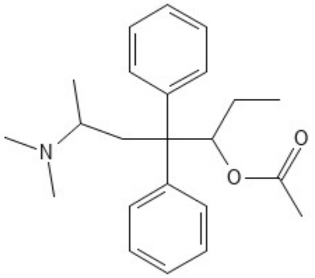	C23H31NO2	**N**	**N**	**Y**	**N**	**N**
**15**	AH-7921	doxylam	3,4-dichloro-N-[[1-(dimethylamino)cyclohexyl]methyl]benzamide	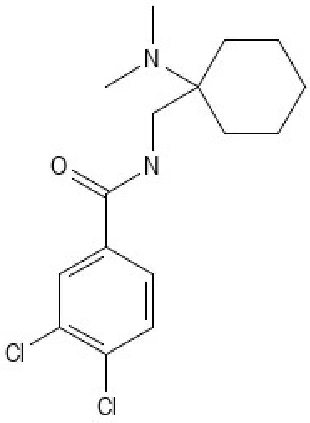	C16H22Cl2N2O	**Y**	**Y**	**Y**	**Y**	**N**

**16**	Alfameprodine	Alphameprodine; Alfameprodina	[(3S,4R)-3-ethyl-1-methyl-4-phenylpiperidin-4-yl] propanoate	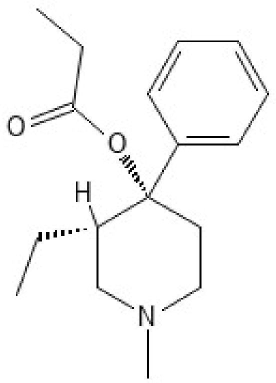	C17H25NO2	**N**	**N**	**Y**	**N**	**N**
**17**	Allylprodine	Alperidine	(1-methyl-4-phenyl-3-prop-2-enylpiperidin-4-yl) propanoate	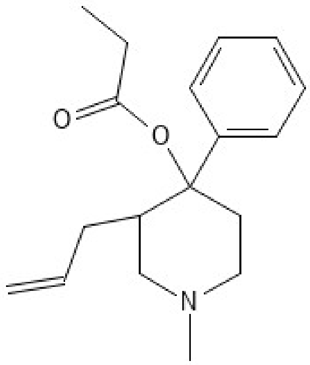	C18H25NO2	**N**	**N**	**Y**	**N**	**N**
**18**	Alpha-3-acetoxy-6-methylanino-4,4-diphenylheptane		N.A	N.A	N.A	**N**	**N**	**N**	**N**	**Y**

**19**	Alphacetylmethadol	α-acetylmethadol; AAM; Alphacemethadone	[(3R,6R)-6-(dimethylamino)-4,4-diphenylheptan-3-yl] acetate	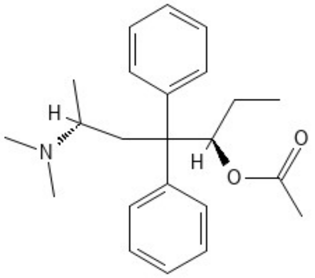	C23H31NO2	**N**	**N**	**Y**	**N**	**N**
**20**	Alphamethadol		(3R,6R)-6-(dimethylamino)-4,4-diphenylheptan-3-ol	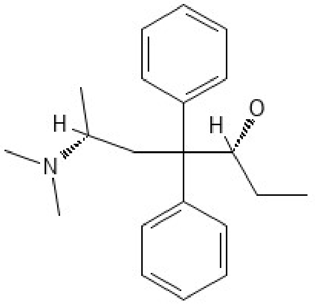	C21H29NO	**N**	**N**	**Y**	**N**	**N**

**21**	Alphaprodine		[(3S,4R)-1,3-dimethyl-4-phenylpiperidin-4-yl] propanoate	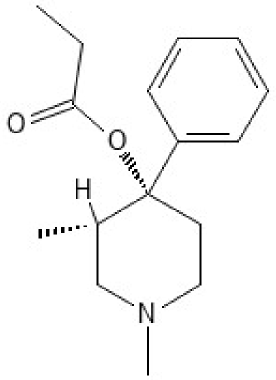	C16H23NO2	**N**	**N**	**Y**	**N**	**N**
**22**	BDPC	bromadol	4-(4-bromophenyl)-4-(dimethylamino)-1-(2-phenylethyl)cyclohexan-1-ol	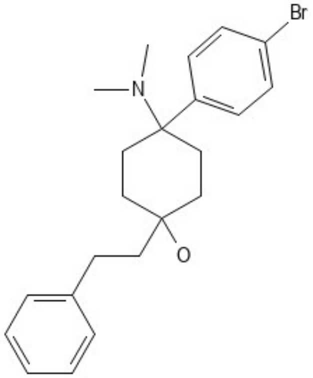	C22H28BrNO	**N**	**N**	**N**	**N**	**Y**

**23**	Benzethidine		ethyl 4-phenyl-1-(2-phenylmethoxyethyl)piperidine-4-carboxylate	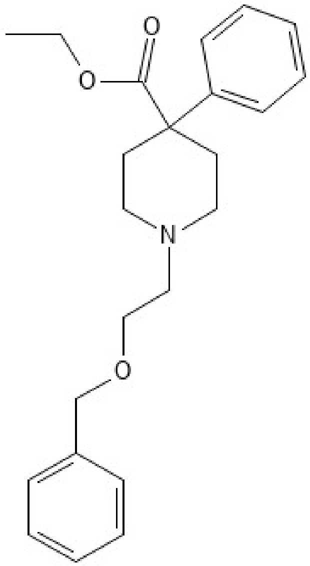	C23H29NO3	**N**	**N**	**Y**	**N**	**N**

**24**	Benzylmorphine myristate	Myrophine; Myristylbenzylmorphine;	[(4R,4aR,7S,7aR,12bS)-3-methyl-9-phenylmethoxy-2,4,4a,7,7a,13-hexahydro-1H-4,12-methanobenzofuro[3,2-e]isoquinolin-7-yl] tetradecanoate	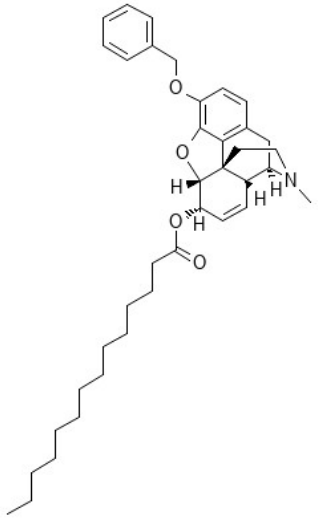	C38H51NO4	**N**	**N**	**N**	**N**	**Y**
**25**	Betacetylmethadol		[(3S,6R)-6-(dimethylamino)-4,4-diphenylheptan-3-yl] acetate	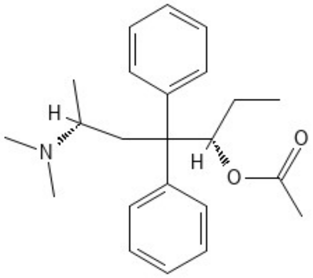	C23H31NO2	**N**	**N**	**Y**	**N**	**N**

**26**	Betameprodin e		[(3R,4R)-3-ethyl-1-methyl-4-phenylpiperidin-4-yl] propanoate	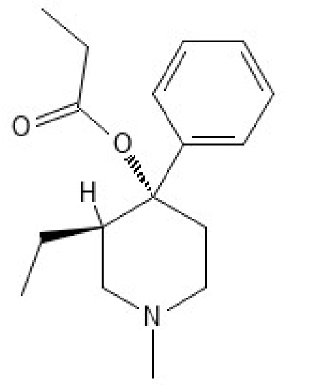	C17H25NO2	**N**	**N**	**Y**	**N**	**N**
**27**	Betamethadol	β-methadol; betametadol	(3S,6R)-6-(dimethylamino)-4,4-diphenylheptan-3-ol	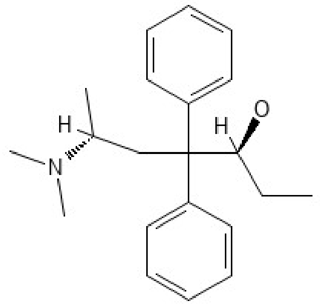	C21H29NO	**N**	**N**	**Y**	**N**	**N**

**28**	Betaprodine		[(3R,4R)-1,3-dimethyl-4-phenylpiperidin-4-yl] propanoate	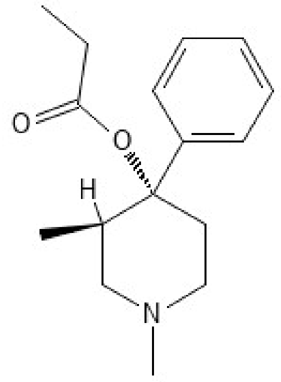	C16H23NO2	**N**	**N**	**Y**	**N**	**N**
**29**	Bromadoline	U-47931E	4-bromo-N-[(1S,2S)-2-(dimethylamino)cyclohexyl]benzamide	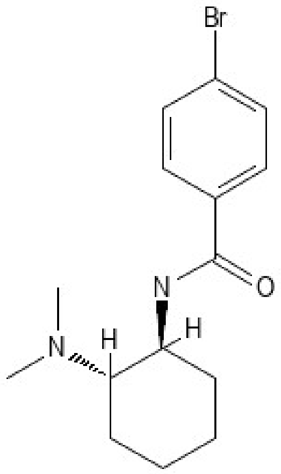	C15H21BrN2O	**Y**	**Y**	**N**	**N**	**N**

**30**	Carperidine		ethyl 1-(3-amino-3-oxopropyl)-4-phenylpiperidine-4-carboxylate	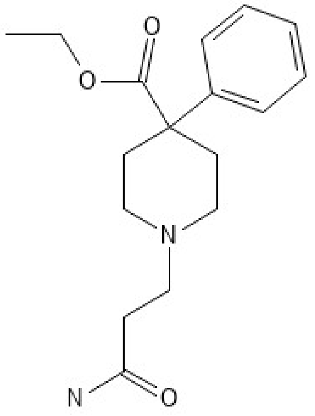	$C_{17}H_{24}N_2O_3$	**N**	**N**	**N**	**N**	**Y**
**31**	Clonitazene		2-[2-[(4-chlorophenyl)methyl]-5-nitrobenzimidazol-1-yl]-N,N-diethylethanamine	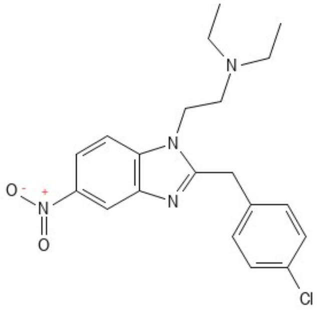	C20H23ClN4O2	**N**	**N**	**Y**	**N**	**N**

**32**	Codeine methylbromide	eucodeine; Eucodin; methobromide (salt)	(4R,4aR,7S,7aR,12bS)-9-methoxy-3,3-dimethyl-2,4,4a,7,7a,13-hexahydro-1H-4,12-methanobenzofuro[3,2-e]isoquinolin-3-ium-7-ol;bromide	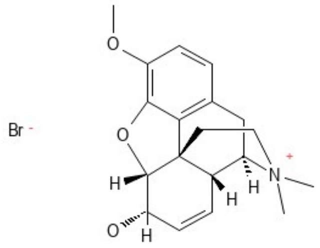	C19H24BrNO3	**N**	**N**	**N**	**N**	**Y**
**33**	Codeine-N-oxide	genocodeine	(4R,4aR,7S,7aR,12bS)-9-methoxy-3-methyl-3-oxido-2,4,4a,7,7a,13-hexahydro-1H-4,12-methanobenzofuro[3,2-e]isoquinolin-3-ium-7-ol	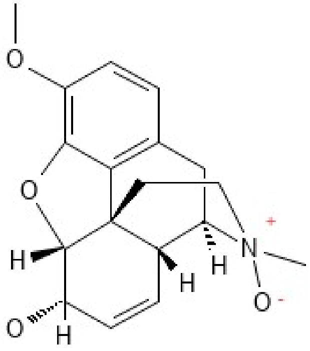	C18H21NO4	**N**	**N**	**N**	**N**	**Y**

**34**	Codoxime	Codossima; Dihydrocodeinone-6-carboxymethyloxime	2-[[(4R,4aR,7aR,12bS)-9-methoxy-3-methyl-1,2,4,4a,5,6,7a,13-octahydro-4,12-methanobenzofuro[3,2-e]isoquinolin-7-ylidene]amino]oxyacetic acid	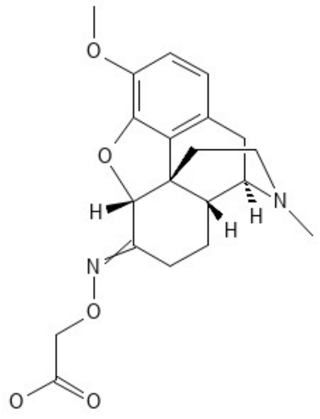	C20H24N2O5	**N**	**N**	**Y**	**N**	**N**

**35**	Cyprenorphine	M-285	(1R,2S,6R,14R,15R,19R)-5-(cyclopropylmethyl)-19-(2-hydroxypropan-2-yl)-15-methoxy-13-oxa-5-azahexacyclo[13.2.2.12,8.01,6.02,14.012,20]icosa-8(20),9,11,16-tetraen-11-ol	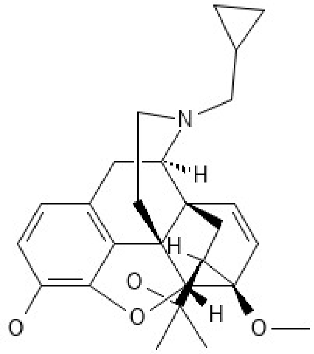	C26H33NO4	**N**	**N**	**N**	**N**	**Y**

**36**	D3-Codeine	Codeine-d3 solution	(4R,4aR,7S, 7aR,12bS)-9-methoxy-3-(trideuteriomethyl)-2,4,4a,7,7a,13-hexahydro-1H-4,12-methanobenzofuro[3,2-e]isoquinolin-7-ol	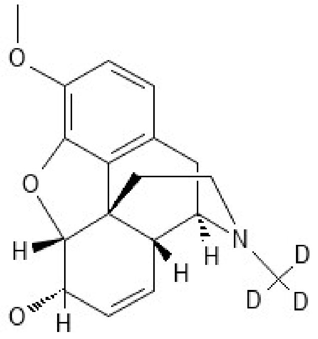	C18H21NO3	**N**	**N**	**N**	**N**	**Y**
**37**	D3-Morphine	Deuterio morphine; morphine-d3	(4R,4aR,7S, 7aR,12bS)-3-(trideuteriomethyl)-2,4,4a,7,7a,13-hexahydro-1H-4,12-methanobenzofuro[3,2-e]isoquinoline-7,9-diol	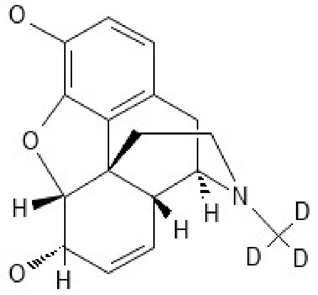	C17H19NO3	**N**	**N**	**N**	**N**	**Y**

**38**	Demethylmorphine	Desmethylmorphine; Normorphine	(4R,4aR,7S,7aR,12bS)-1,2,3,4,4a,7,7a,13-octahydro-4,12-methanobenzofuro[3,2-e]isoquinoline-7,9-diol	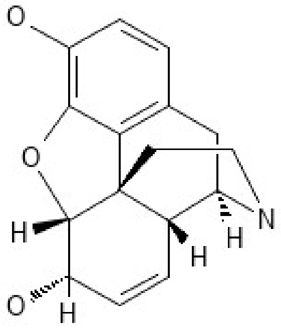	C16H17NO3	**N**	**N**	**Y**	**N**	**N**
**39**	Desmetramadol	O-desmethyltramadol; ODT; O-DSMT; Omnitram; Krypton (powdered kratom leaf laced with desmetramadol)	3-[(1R,2R)-2-[(dimethylamino)methyl]-1-hydroxycyclohexyl]phenol	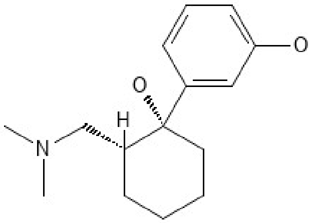	C15H23NO2	**Y**	**N**	**N**	**N**	**N**

**40**	Desomorphine	krokodil; dihydrodesoxymorphine; Permonid	(4R,4aR,7aS,12bS)-3-methyl-2,4,4a,5,6,7,7a,13-octahydro-1H-4,12-methanobenzofuro[3,2-e]isoquinolin-9-ol	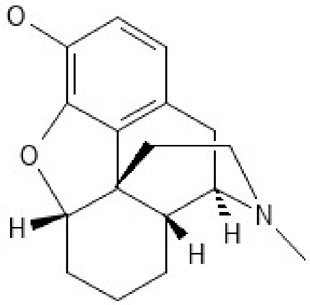	C17H21NO2	**N**	**N**	**Y**	**N**	**N**
**41**	Diampromide		N-[2-[methyl(2-phenylethyl)amino]propyl]-N-phenylpropanamide	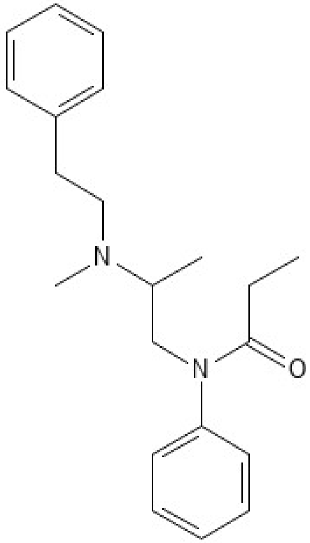	C21H28N2O	**N**	**N**	**Y**	**N**	**N**

**42**	Diethylthiambutene	Thiambutene; Themalon; Diethibutin	N,N-diethyl-4,4-dithiophen-2-ylbut-3-en-2-amine	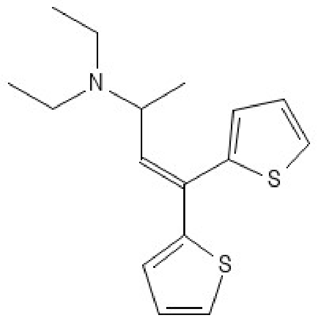	C16H21NS2	**N**	**N**	**Y**	**N**	**N**
**43**	Dihydroetorphine	DHE	(1S,2S,6R,14R,15R,16R)-16-[(2R)-2-hydroxypentan-2-yl]-15-methoxy-5-methyl-13-oxa-5-azahexacyclo[13.2.2.12,8.01,6.02,14.012,20]icosa-8(20),9,11-trien-11-ol	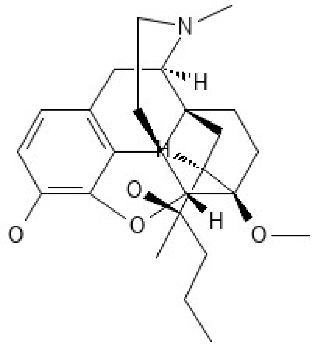	C25H35NO4	**N**	**N**	**Y**	**N**	**N**

**44**	Dihydromorphine	Paramorfan; Paramorphan	(4R,4aR,7S,7aR,12bS)-3-methyl-2,4,4a,5,6,7,7a,13-octahydro-1H-4,12-methanobenzofuro[3,2-e]isoquinoline-7,9-diol	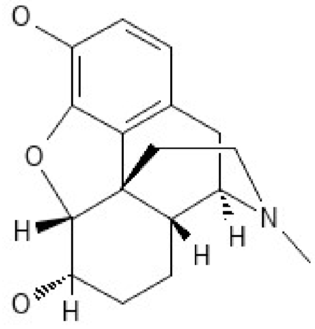	C17H21NO3	**N**	**N**	**Y**	**N**	**N**
**45**	Dihydromorphine 3,6-diglucuronide		N.A	N.A	N.A	**N**	**N**	**N**	**N**	**Y**
**46**	Dimenoxadol		2-(dimethylamino)ethyl 2-ethoxy-2,2-diphenylacetate	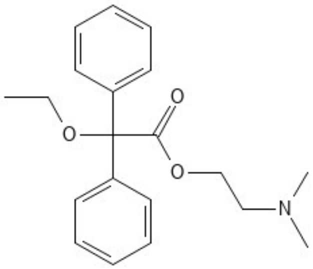	C20H25NO3	**N**	**N**	**Y**	**N**	**N**

**47**	Dimepheptanol	Amidol; Pangerin; methadol; racemethadol; imethadol; betamethadol	6-(dimethylamino)-4,4-diphenylheptan-3-ol	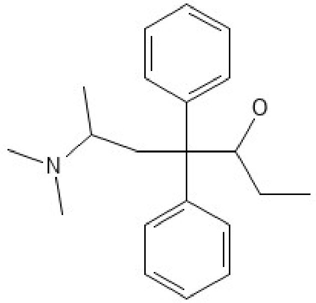	C21H29NO	**N**	**N**	**Y**	**N**	**N**
**48**	Dimethylthiambutene	DMTB; Ohton; Aminobutene; Dimethibutin; Kobaton; Takaton; Dimethibutin	N,N-dimethyl-4,4-dithiophen-2-ylbut-3-en-2-amine	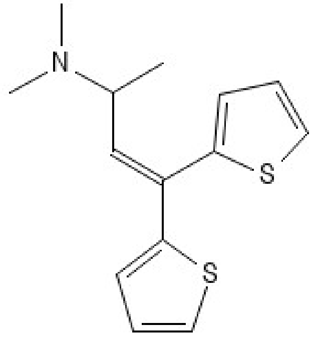	C14H17NS2	**N**	**N**	**Y**	**N**	**N**

**49**	Dioxaphetyl butyrate	Amidalgon; Spasmoxal	ethyl 4-morpholin-4-yl-2,2-diphenylbutanoate	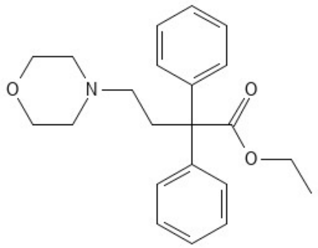	C22H27NO3	**N**	**N**	**Y**	**N**	**N**
**50**	Dipipanone	Pipadone; Diconal (commercially available, dipipanone hydrochloride mixed with cyclizine hydrochloride)	4,4-diphenyl-6-piperidin-1-ylheptan-3-one	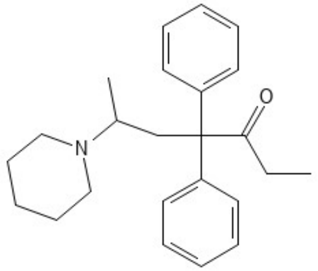	C24H31NO	**N**	**N**	**Y**	**N**	**N**

**51**	Drotebanol	Oxymethebanol; Metebanyl	(1R,9R,10S,13R)-3,4-dimethoxy-17-methyl-17-azatetracyclo[7.5.3.01,10.02,7]heptadeca-2(7),3,5-triene-10,13-diol l	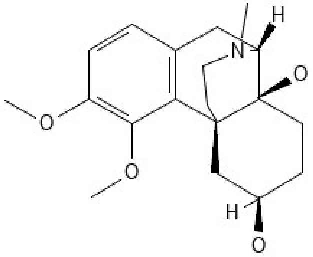	C19H27NO4	**N**	**N**	**Y**	**N**	**N**
**52**	Ethoheptazine	Zactane; Equagesic (commercially available, ethoheptazine citrate mixed with acetylsalicylic acid and meprobamate)	ethyl 1-methyl-4-phenylazepane-4-carboxylate	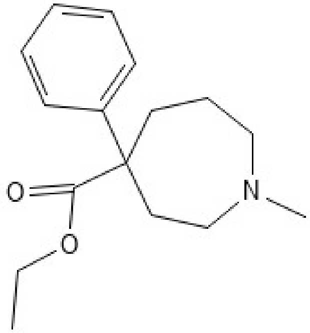	C16H23NO2	**N**	**N**	**N**	**N**	**Y**

**53**	Ethylmethylthiambutene	Emethibutin	N-ethyl-N-methyl-4,4-dithiophen-2-ylbut-3-en-2-amine	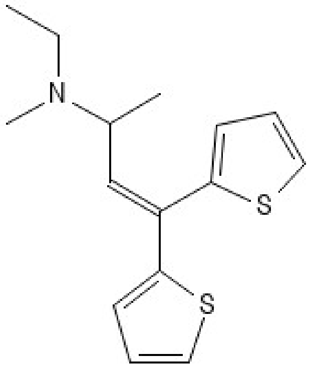	C15H19NS2	**N**	**N**	**Y**	**N**	**N**
**54**	Etonitazene		2-[2-[(4-ethoxyphenyl)methyl]-5-nitrobenzimidazol-1-yl]-N,N-diethylethanamine	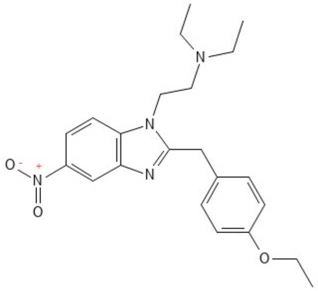	C22H28N4O3	**N**	**N**	**Y**	**N**	**N**

**55**	Etorphine	M99; Immobilon	(1R,2S,6R,14R,15R,19R)-19-[(2R)-2-hydroxypentan-2-yl]-15-methoxy-5-methyl-13-oxa-5-azahexacyclo[13.2.2.12,8.01,6.02,14.012,20]icosa-8(20),9,11,16-tetraen-11-ol	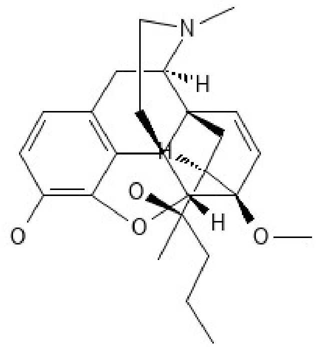	C25H33NO4	**N**	**N**	**Y**	**N**	**N**
**56**	Etorphine hydrochloride		(1R,2S,6R,14R,15R,19R)-19-[(2R)-2-hydroxypentan-2-yl]-15-methoxy-5-methyl-13-oxa-5-azahexacyclo[13.2.2.12,8.01,6.02,14.012,20]icosa-8(20),9,11,16-tetraen-11-	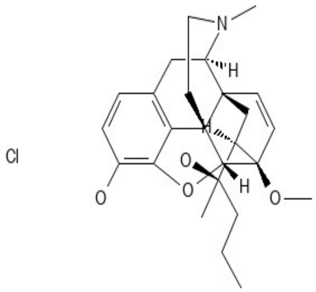	C25H34ClNO4	**N**	**N**	**N**	**N**	**Y**

			ol;hydrochloride							
**57**	Etoxeridine	Carbetidine; Atenos	ethyl 1-[2-(2-hydroxyethoxy)ethyl]-4-phenylpiperidine-4-carboxylate	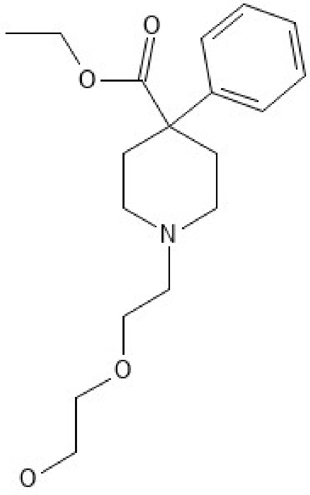	C18H27NO4	**N**	**N**	**Y**	**N**	**N**

**58**	Furethidine		ethyl 1-[2-(oxolan-2-ylmethoxy)ethyl]-4-phenylpiperidine-4-carboxylate	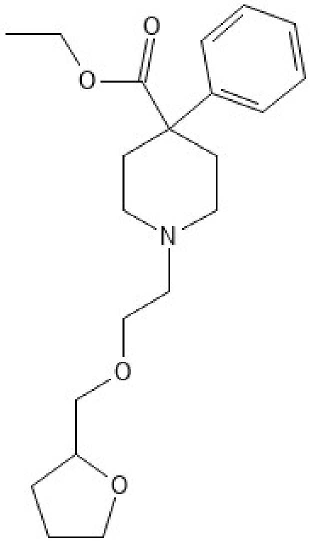	C21H31NO4	**N**	**N**	**Y**	**N**	**N**
**59**	Hexadeuterated diamorphine		N.A	N.A	N.A	**N**	**N**	**N**	**N**	**Y**

**60**	Hydromorphinol	RAM-320; 14-hydroxydihydromorphine; α-oxymorphol	(4R,4aS,7S,7aR,12bS)-3-methyl-1,2,4,5,6,7,7a,13-octahydro-4,12-methanobenzofuro[3,2-e]isoquinoline-4a,7,9-triol	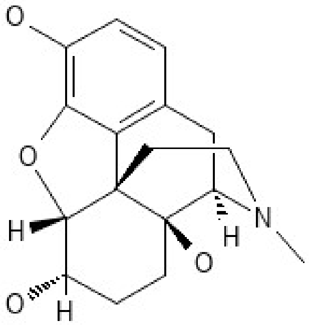	C17H21NO4	**N**	**N**	**Y**	**N**	**N**
**61**	Hydroxypethidine	Bemidone	ethyl 4-(3-hydroxyphenyl)-1-methylpiperidine-4-carboxylate	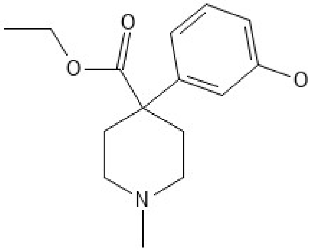	C15H21NO3	**N**	**N**	**Y**	**N**	**N**

**62**	Isocodeine	6-Isocodeine; α-Isocodeine	(4R,4aR,7R,7aR,12bS)-9-methoxy-3-methyl-2,4,4a,7,7a,13-hexahydro-1H-4,12-methanobenzofuro[3,2-e]isoquinolin-7-ol	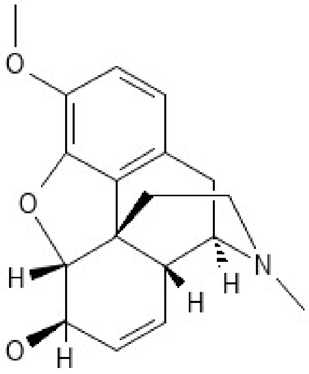	C18H21NO3	**N**	**N**	**N**	**N**	**Y**
**63**	Isomethadone	Liden; isoamidone	6-(dimethylamino)-5-methyl-4,4-diphenylhexan-3-one	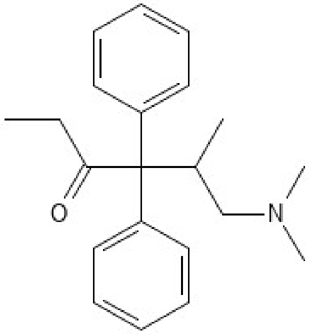	C21H27NO	**N**	**N**	**Y**	**N**	**N**

**64**	Isopropyl-U-47700	Isopropyl-U47; IP-U47	3,4-dichloro-N-[(1R,2R)-2-(dimethylamino)cyclohexyl]-N-propan-2-ylbenzamide	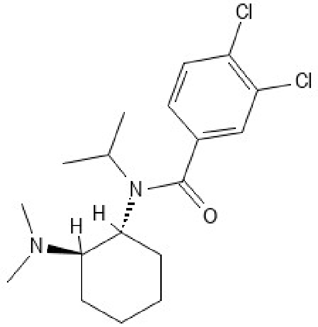	C18H26Cl2N2O	**Y**	**Y**	**N**	**N**	**N**
**65**	Lefetamine	SPA; Santenol	(1R)-N,N-dimethyl-1,2-diphenylethanamine	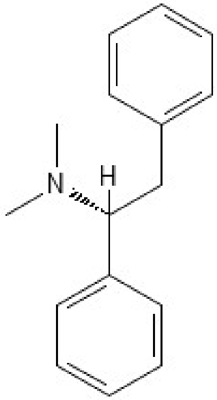	C16H19N	**N**	**N**	**N**	**Y**	**N**

**66**	Levomethorphan		(1R,9R,10R)-4-methoxy-17-methyl-17-azatetracyclo[7.5.3.01,10.02,7]heptadeca-2(7),3,5-triene	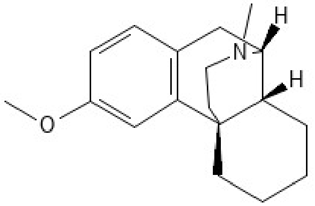	C18H25NO	**N**	**N**	**Y**	**N**	**N**
**67**	Levomoramide		(3R)-3-methyl-4-morpholin-4-yl-2,2-diphenyl-1-pyrrolidin-1-ylbutan-1-one	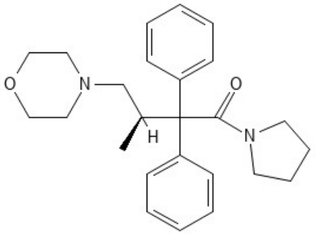	C25H32N2O2	**N**	**N**	**Y**	**N**	**N**

**68**	Levophenacylmorphan	Benzorphanol	2-[(1R,9R,10R)-4-hydroxy-17-azatetracyclo[7.5.3.01,10.02,7]heptadeca-2(7),3,5-trien-17-yl]-1-phenylethanone	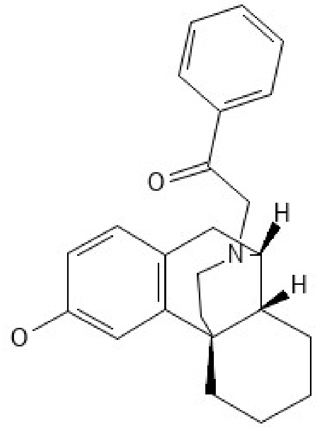	C24H27NO2	**N**	**N**	**Y**	**N**	**N**
**69**	Levorphanol	L-Dromoran; Levo-Dromoran; Ro 1-5431	(1R,9R,10R)-17-methyl-17-azatetracyclo[7.5.3.01,10.02,7]heptadeca-2(7),3,5-trien-4-ol	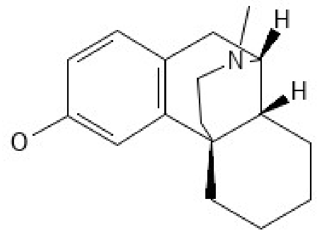	C17H23NO	**N**	**N**	**Y**	**N**	**N**

**70**	Meprodine	(α/β)-Meprodine	[(3S,4R)-3-ethyl-1-methyl-4-phenylpiperidin-4-yl] propanoate	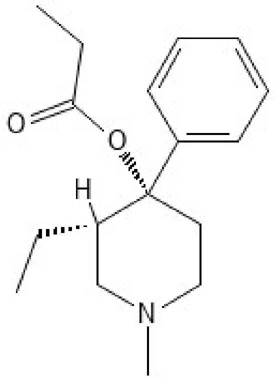	C17H25NO2	**N**	**N**	**N**	**N**	**Y**
**71**	Metazocine		(1R,9R)-1,10,13-trimethyl-10-azatricyclo[7.3.1.02,7]trideca-2(7),3,5-trien-4-ol	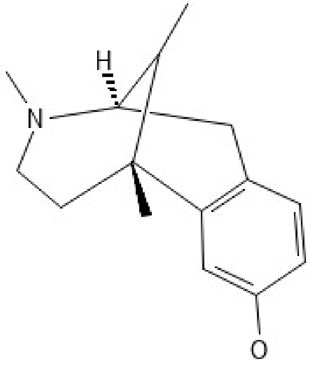	C15H21NO	**N**	**N**	**Y**	**N**	**N**

**72**	Metethoheptazine	WY-535	ethyl 1,3-dimethyl-4-phenylazepane-4-carboxylate	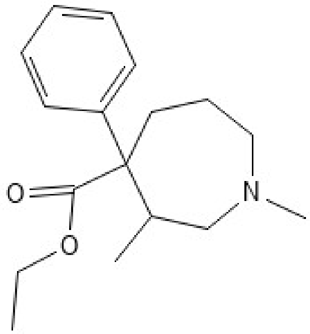	C17H25NO2	**N**	**N**	**N**	**N**	**Y**
**73**	Metheptazine		methyl 1,2-dimethyl-4-phenylazepane-4-carboxylate	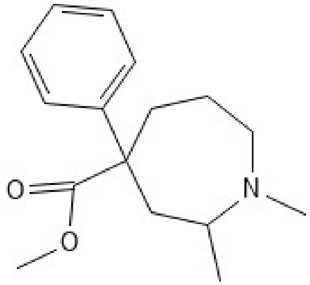	C16H23NO2	**N**	**N**	**N**	**N**	**Y**
**74**	Methorphan	dextromethorphan; levomethorphan; racemethorphan	(1R,9R,10R)-4-methoxy-17-methyl-17-azatetracyclo[7.5.3.01,10.02,7]heptadeca-2(7),3,5-triene	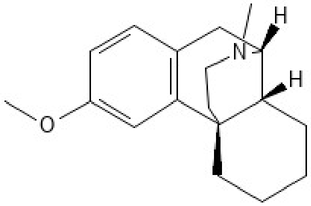	C18H25NO	**N**	**N**	**N**	**N**	**Y**

**75**	Methyldihydromorphine	dihydroheterocodeine	(4R,4aR,7S,7aR,12bS)-3,7-dimethyl-1,2,4,4a,5,6,7a,13-octahydro-4,12-methanobenzofuro[3,2-e]isoquinoline-7,9-diol	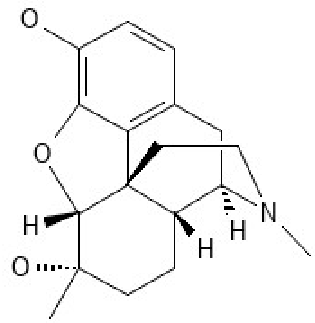	C18H23NO3	**N**	**N**	**Y**	**N**	**N**
**76**	Metopon	Methyldihydromorphinone	(4R,4aR,7aR,12bS)-9-hydroxy-3,7a-dimethyl-2,4,4a,5,6,13-hexahydro-1H-4,12-methanobenzofuro[3,2-e]isoquinolin-7-one	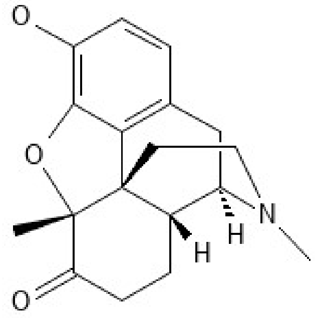	C18H21NO3	**N**	**N**	**Y**	**N**	**N**

**77**	Moramide intermediate	2-Methyl-3-morpholino-1,1-diphenylpropane-carboxylic acid	3-methyl-4-morpholin-4-yl-2,2-diphenylbutanoic acid	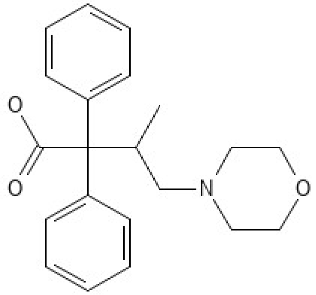	C21H25NO3	**N**	**N**	**Y**	**N**	**N**
**78**	Morphanol	Racemorphan (Dextrorphan and Levorphan); Morphinan-3-ol, 17-methyl-, (+/-)-	17-methyl-17-azatetracyclo[7.5.3.01,10.02,7]heptadeca-2(7),3,5-trien-4-ol	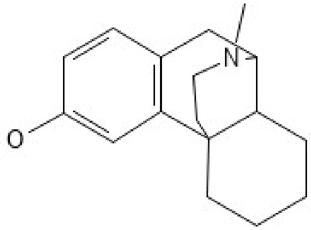	C17H23NO	**N**	**N**	**N**	**N**	**Y**

**79**	Morpheridine	Morpholinoethylnorpethidine	ethyl 1-(2-morpholin-4-ylethyl)-4-phenylpiperidine-4-carboxylate	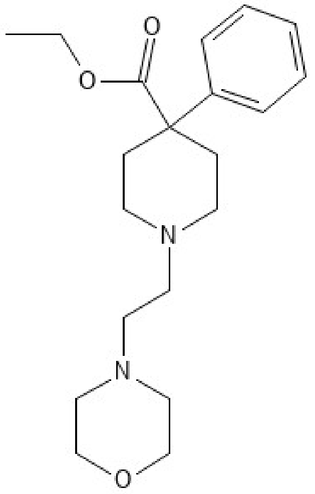	C20H30N2O3	**N**	**N**	**Y**	**N**	**N**
**80**	Morphine Methobromide	Morphine methylbromide; Morphine bromomethylate; Morphosan	(4R,4aR,7S,7aR,12bS)-3,3-dimethyl-2,4,4a,7,7a,13-hexahydro-1H-4,12-methanobenzofuro[3,2-e]isoquinolin-3-ium-7,9-diol;bromide	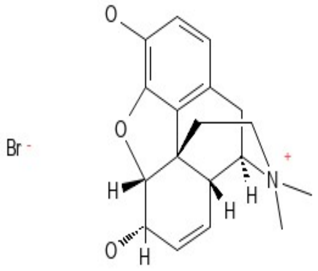	C18H22BrNO3	**N**	**N**	**Y**	**N**	**N**

**81**	Morphine methylsulfonate		[(4R,4aR,7S,7aR,12bS)-7-hydroxy-3-methyl-2,4,4a,7,7a,13-hexahydro-1H-4,12-methanobenzofuro[3,2-e]isoquinolin-9-yl] methanesulfonate	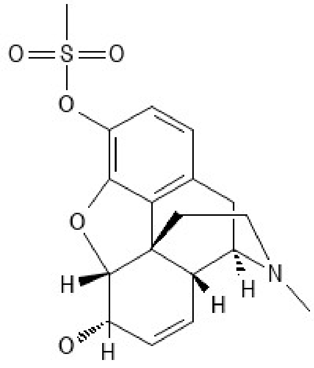	C18H21NO5S	**N**	**N**	**N**	**N**	**Y**
**82**	Morphine N-oxide	Morphine-N-Oxide; genomorphine	(4R,4aR,7S,7aR,12bS)-3-methyl-3-oxido-2,4,4a,7,7a,13-hexahydro-1H-4,12-methanobenzofuro[3,2-e]isoquinolin-3-ium-7,9-diol	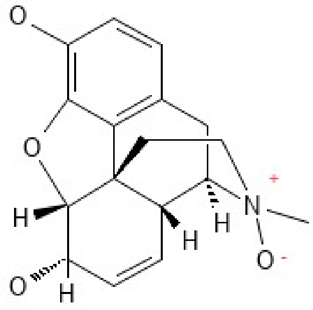	C17H19NO4	**N**	**N**	**Y**	**N**	**N**

**83**	Morphine3, 6-diacalte		N.A	N.A	N.A	**N**	**N**	**N**	**N**	**Y**
**84**	Morphine-6-sulphate	M6S; Morphine 6-sulfate	[(4R,4aR,7S,7aR,12bS)-9-hydroxy-3-methyl-2,4,4a,7,7a,13-hexahydro-1H-4,12-methanobenzofuro[3,2-e]isoquinolin-7-yl] hydrogen sulfate	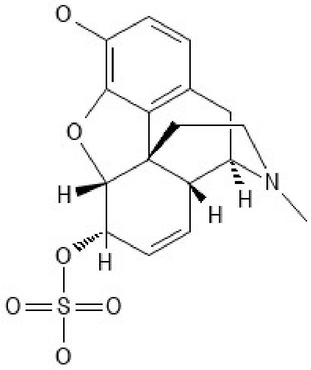	C17H19NO6S	**N**	**N**	**N**	**N**	**Y**

**85**	MT-45	IC-6	1-Cyclohexyl-4-(1,2-diphenylethyl)piperazine	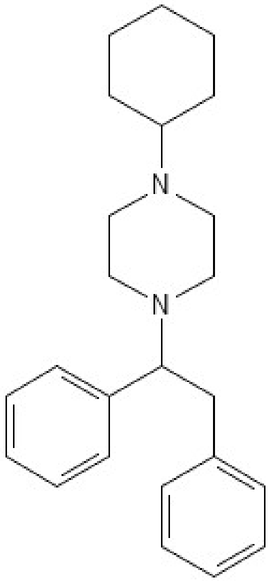	C24H32N2	**Y**	**N**	**Y**	**N**	**N**
**86**	N-benzylpethidine		N.A	N.A	N.A	**N**	**N**	**N**	**N**	**Y**

**87**	N-Demethylcodeine	Norcodeine	(4R,4aR,7S,7aR,12bS)-9-methoxy-1,2,3,4,4a,7,7a,13-octahydro-4,12-methanobenzofuro[3,2-e]isoquinolin-7-ol	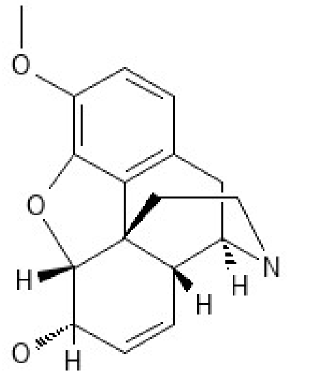	C17H19NO3	**N**	**N**	**Y**	**N**	**N**
**88**	Nicocodeine	Nicocodine; Lyopect; 6-Nicotinoylcodeine	[(4R,4aR,7S,7aR,12bS)-9-methoxy-3-methyl-2,4,4a,7,7a,13-hexahydro-1H-4,12-methanobenzofuro[3,2-e]isoquinolin-7-yl] pyridine-3-carboxylate	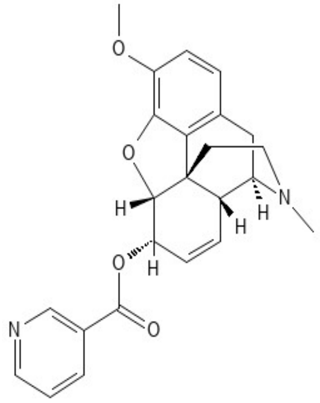	C24H24N2O4	**N**	**N**	**Y**	**N**	**N**

**89**	Nicodicodeine	6-Nicotinoyl dihydrocodeine	[(4R,4aR,7S,7aR,12bS)-9-methoxy-3-methyl-2,4,4a,5,6,7,7a,13-octahydro-1H-4,12-methanobenzofuro[3,2-e]isoquinolin-7-yl] pyridine-3-carboxylate	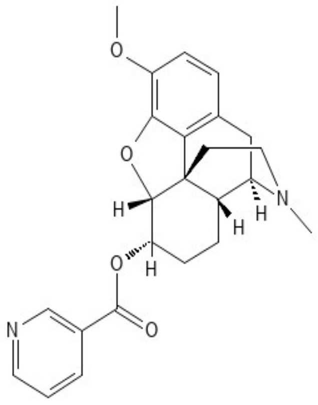	C24H26N2O4	**N**	**N**	**Y**	**N**	**N**
**90**	Noracymethadol	Paracymethadol	[6-(methylamino)-4,4-diphenylheptan-3-yl] acetate	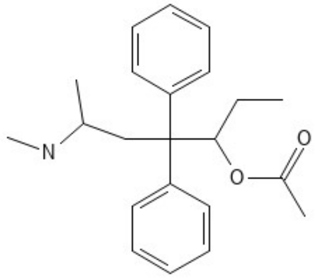	C22H29NO2	**N**	**N**	**Y**	**N**	**N**

**91**	Norlevorphanol	3-Hydroxymorphinan	(1R,9R,10R)-17-azatetracyclo[7.5.3.01,10.02,7]heptadeca-2(7),3,5-trien-4-ol	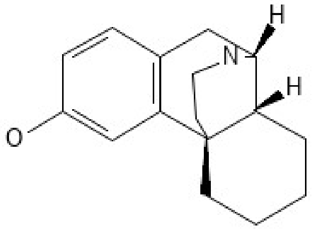	C16H21NO	**N**	**N**	**Y**	**N**	**N**
**92**	Norpethidine	normeperidine; pethidine intermediate B	ethyl 4-phenylpiperidine-4-carboxylate	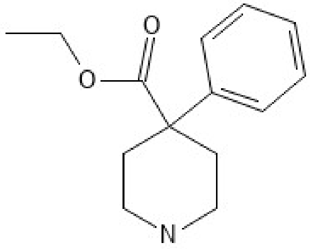	C14H19NO2	**N**	**N**	**Y**	**N**	**N**
**93**	Norpipanone	Hexalgon	4,4-diphenyl-6-piperidin-1-ylhexan-3-one	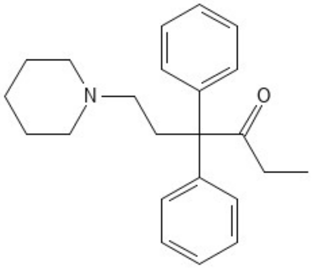	C23H29NO	**N**	**N**	**Y**	**N**	**N**

**94**	Oripavine		(4R,7aR,12bS)-7-methoxy-3-methyl-2,4,7a,13-tetrahydro-1H-4,12-methanobenzofuro[3,2-e]isoquinolin-9-ol	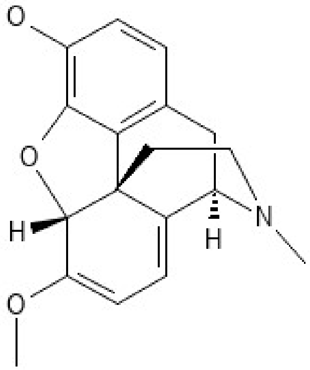	C18H19NO3	**N**	**N**	**Y**	**N**	**N**
**95**	Oxpheneridine	Carbamethidine	ethyl 1-(2-hydroxy-2-phenylethyl)-4-phenylpiperidine-4-carboxylate	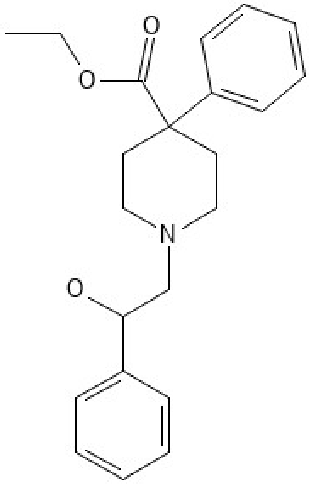	C22H27NO3	**N**	**N**	**N**	**N**	**Y**

**96**	Oxymorphone	Numorphan (oxymorphone hydrochloride); 14-Hydroxydihydromorphinone	(4R,4aS,7aR,12bS)-4a,9-dihydroxy-3-methyl-2,4,5,6,7a,13-hexahydro-1H-4,12-methanobenzofuro[3,2-e]isoquinolin-7-one	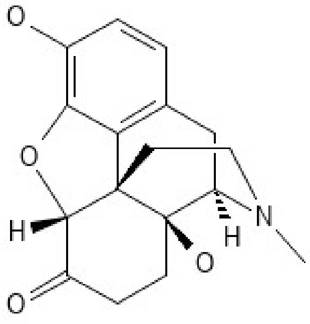	C17H19NO4	**N**	**N**	**Y**	**N**	**N**
**97**	PEPAP	synthetic heroin; 1-(2-Phenylethyl)-4-Phenyl-4-Acetoxypiperidine	4-phenyl-1-(2-phenylethyl) piperidin-4-yl acetate	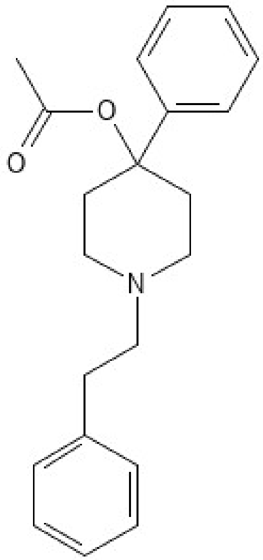	C21H25NO2	**N**	**N**	**Y**	**N**	**N**

**98**	Phenadoxone	Heptalgin; Morphidone; Heptazone	6-morpholin-4-yl-4,4-diphenylheptan-3-one	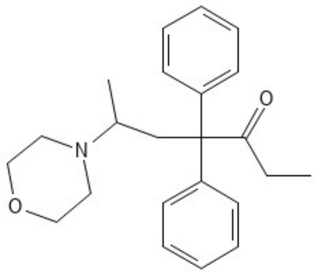	C23H29NO2	**N**	**N**	**Y**	**N**	**N**
**99**	Phenampromide		N-phenyl-N-(1-piperidin-1-ylpropan-2-yl)propanamide	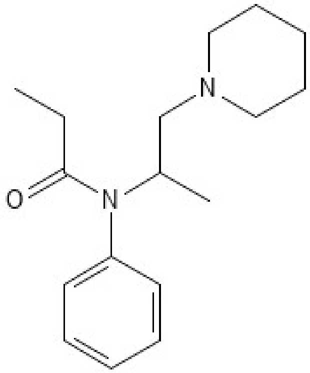	C17H26N2O	**N**	**N**	**Y**	**N**	**N**

**100**	Phenomorphan		(1R,9R,10R)-17-(2-phenylethyl)-17-azatetracyclo[7.5.3.01,10.02,7]heptadeca-2(7),3,5-trien-4-ol	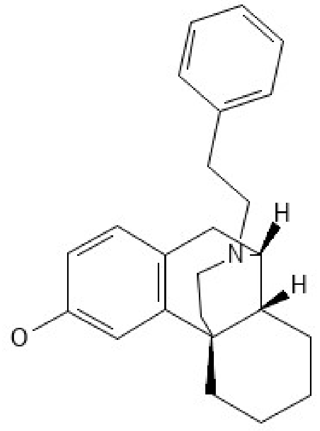	C24H29NO	**N**	**N**	**Y**	**N**	**N**
**101**	Piminodine	Alvodine	ethyl 1-(3-anilinopropyl)-4-phenylpiperidine-4-carboxylate	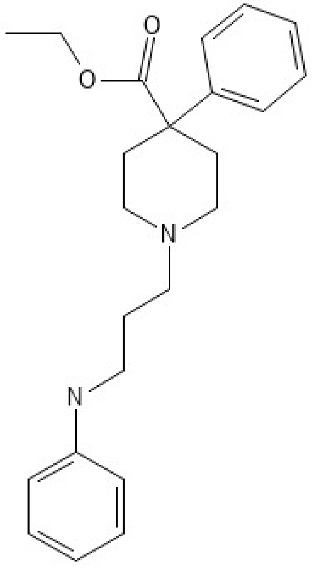	C23H30N2O2	**N**	**N**	**Y**	**N**	**N**

**102**	Piperidylthiambutene	Piperidino-ohton; Piperidinohton; 3-Piperidino-1,1-di(2-thienyl)but-1-ene	1-(4,4-dithiophen-2-ylbut-3-en-2-yl)piperidine	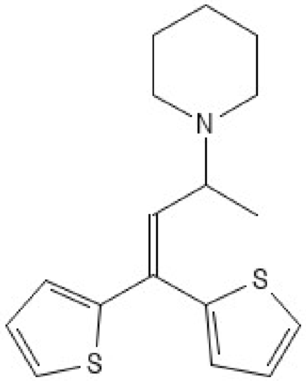	C17H21NS2	**Y**	**Y**	**N**	**N**	**N**
**103**	Proheptazine		(1,3-dimethyl-4-phenylazepan-4-yl) propanoate	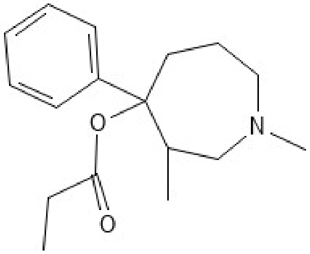	C17H25NO2	**N**	**N**	**Y**	**N**	**N**
**104**	Properidine	Ipropethidine	propan-2-yl 1-methyl-4-phenylpiperidine-4-carboxylate	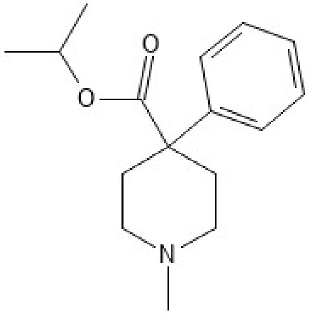	C16H23NO2	**N**	**N**	**Y**	**N**	**N**

**105**	Propiram	Algeril; Dirame; Bay 4503	N-(1-piperidin-1-ylpropan-2-yl)-N-pyridin-2-ylpropanamide	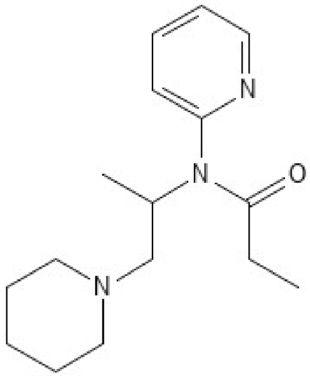	C16H25N3O	**N**	**N**	**Y**	**N**	**N**
**106**	Racemethorphan	see methorphan	4-methoxy-17-methyl-17-azatetracyclo[7.5.3.01,10.02,7]heptadeca-2(7),3,5-triene	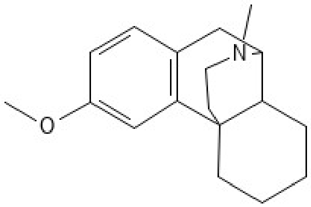	C18H25NO	**N**	**N**	**Y**	**N**	**N**

**107**	Racemoramide	Moramide	3-methyl-4-morpholin-4-yl-2,2-diphenyl-1-pyrrolidin-1-ylbutan-1-one	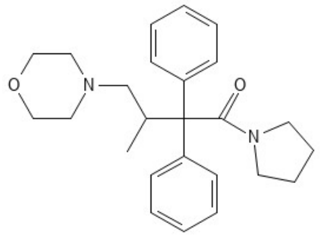	C25H32N2O2	**N**	**N**	**Y**	**N**	**N**
**108**	Trimeperidine	Isopromedol	[(2S,4S,5R)-1,2,5-trimethyl-4-phenylpiperidin-4-yl] propanoate	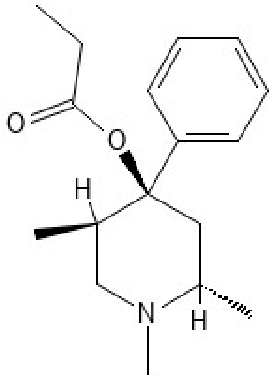	C17H25NO2	**N**	**N**	**Y**	**N**	**N**

**109**	U-47700	Pinky; Fake morphine; U4	3,4-dichloro-N-[(1R,2R)-2-(dimethylamino)cyclohexyl]-N-methylbenzamide	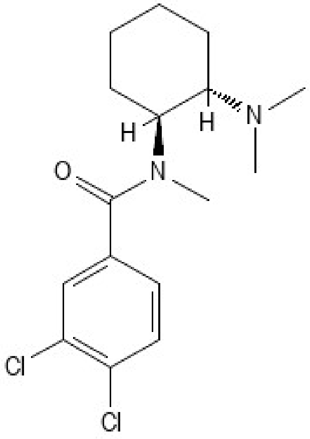	C16H22Cl2N2O	**Y**	**Y**	**Y**	**N**	**N**
**110**	U-48800		2-(2,4-dichlorophenyl)-N-[(1S,2S)-2-(dimethylamino)cyclohexyl]-N-methylacetamide	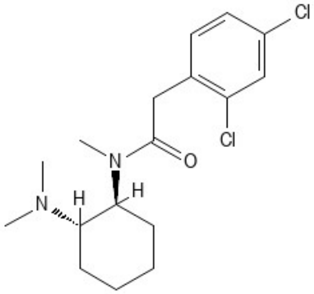	C17H24Cl2N2O	**Y**	**Y**	**N**	**N**	**N**

**111**	U-49900		3,4-dichloro-N-(2-(diethylamino)cyclohexyl)-N-methylbenzamide	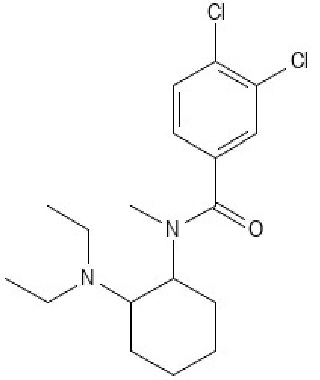	C18H26Cl2N2O	**Y**	**N**	**N**	**N**	**N**
**112**	U-51754	methene-U-47700	2-(3,4-dichlorophenyl)-N-[2-(dimethylamino)cyclohexyl]-N-methylacetamide	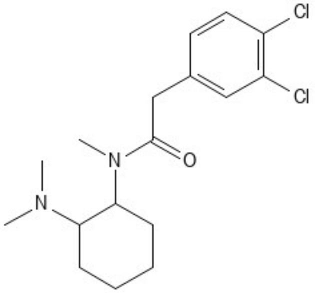	C17H24Cl2N2O	**Y**	**Y**	**N**	**N**	**N**

**113**	W-15	1-Phenylethylpiperidylidene-2-(4-chlorophenyl)sulfonamide	4-chloro-N-[1-(2-phenylethyl)piperidin-2-ylidene]benzenesulfonamide	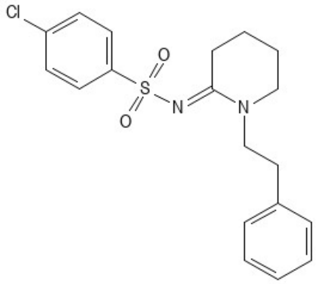	C19H21ClN2O2S	**Y**	**N**	**N**	**N**	**N**

Table S2Ca – NPSfinder® non fentanyl µ(mu)-opioid receptor agonists including those with partial/weak and potent/very potent action (miscellaneous opioids); comparison between the different databases. NPSfinder® crawling in 2018. (updated table from Arillotta et al., 2020, Table 2C)

N	Molecule denomination in NPS*finder*®	Other Names	Chemical Name (IUPAC)	Chemical Structure	Molecular Formula	UNODC EWA on NPS (May 2025)	CFSRE (NPS Discovery) (December 2024)	INCB Yellow list (July 2024)	INCB Green list (January 2025)	Unique to NPS*finder*® database
**1**	Cyclazocine		10-(cyclopropylmethyl)-1,13-dimethyl-10-azatricyclo[7.3.1.02,7]trideca-2(7),3,5-trien-4-ol	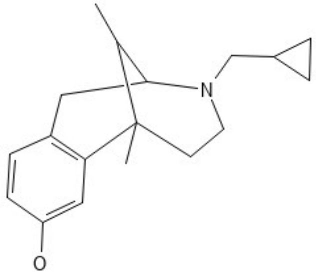	C18H25NO	**N**	**N**	**N**	**N**	**Y**
**2**	Levallorphan*	levallorphan tartrate; Lorfan; Naloxifan; Naloxiphan; Pethilorfan (combination of levallorph	(1R,9R,10R)-17-prop-2-enyl-17-azatetracyclo[7.5.3.01,10.02,7]heptadeca-2(7),3,5-trien-4-ol	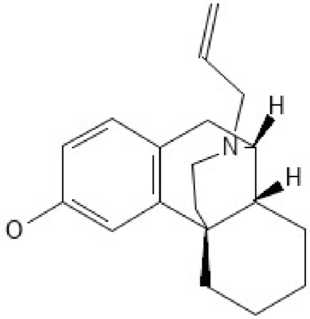	C19H25NO	**N**	**N**	**N**	**N**	**Y**

		an with pethidine (meperidine))								
**3**	Levargorphan *		l-11-propargyl-1,2,3,9,10,10a-hexahydro-4H-10,4a-iminoethanophenanthren-6-ol	N.A	N.A	**N**	**N**	**N**	**N**	**Y**
**4**	Salvinorin A		methyl (2S,4aR,6aR,7R,9S,10aS,10bR)-9-acetyloxy-2-(furan-3-yl)-6a,10b-dimethyl-4,10-dioxo-2,4a,5,6,7,8,9,10a-octahydro-1H-benzo[f]isoc	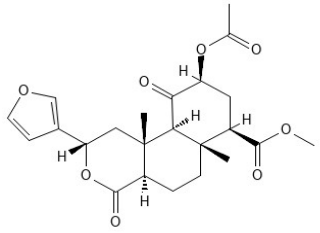	$C_{23}H_{28}O_8$	**N**	**N**	**N**	**N**	**Y**

			hromene-7-carboxylate							
**5**	Salvinorin B ethoxymethyl ether	Symmetry	methyl (2S,4aR,6aR,7R,9S,10aS,10bR)-9-(ethoxymethoxy)-2-(furan-3-yl)-6a,10b-dimethyl-4,10-dioxo-2,4a,5,6,7,8,9,10a-octahydro-1H-benzo[f]isochromene-7-carboxylate	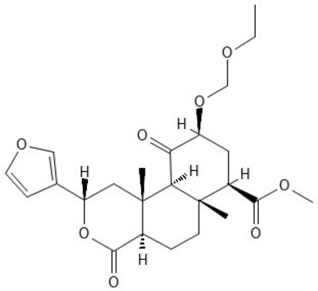	$C_{24}H_{32}O_8$	**N**	**N**	**N**	**N**	**Y**
**6**	Salvinorin B methoxymethyl ether		methyl (2S,4aR,6aR,7R,9S,10aS,10bR)-2-(furan-3-yl)-9-(methoxymethoxy)-6a,10b-	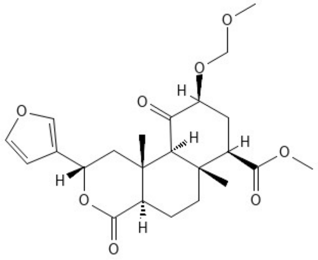	$C_{23}H_{30}O_8$	**N**	**N**	**N**	**N**	**Y**

			dimethyl-4,10-dioxo-2,4a,5,6,7,8,9,10a-octahydro-1H-benzo[f]isochromene-7-carboxylate							
**7**	Thebaine		(4R,7aR,12bS)-7,9-dimethoxy-3-methyl-2,4,7a,13-tetrahydro-1H-4,12-methanobenzofuro[3,2-e]isoquinoline	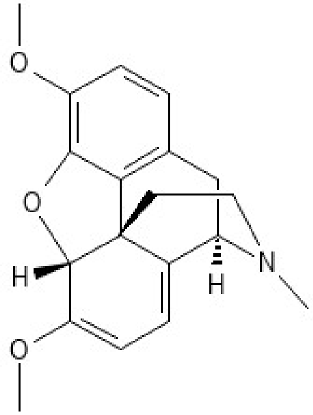	$C_{19}H_{21}NO_3$	**N**	**N**	**Y**	**N**	**N**

**8**	Tifluadom		N-[[5-(2-fluorophenyl)-1-methyl-2,3-dihydro-1,4-benzodiazepin-2-yl]methyl]thiophene-3-carboxamide	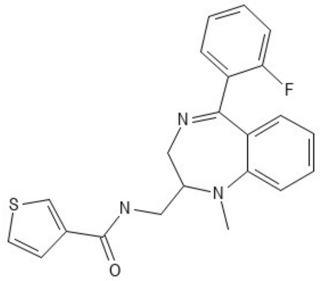	$C_{22}H_{20}FN_3OS$	**N**	**N**	**N**	**N**	**Y**
**9**	U-50488	U-50488H; trans-3,4-Dichloro-N-methyl-N-[2-(1-pyrrolidinyl)cyclohexyl]-benzeneacetamide	2-(3,4-dichlorophenyl)-N-methyl-N-[(1R,2R)-2-pyrrolidin-1-ylcyclohexyl]acetamide	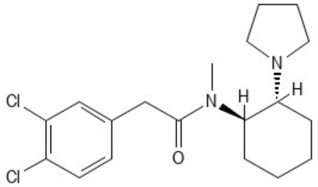	$C_{19}H_{26}Cl_2N_2O$	**Y**	**N**	**N**	**N**	**N**

*Levallorphan and Levargorphan also acting as antagonists in (mu) µ-receptors.

Table S2Cb – NPS.Finder® non fentanyl κ(kappa)-opioid receptor agonists including those with partial/weak and potent action (miscellaneous opioids); comparison between the different databases. NPS.Finder® crawling in 2018. (updated table from Arillotta et al., 2020, Table 2C)

N	Molecule denomination in NPSfinder®	Other Names	Chemical Name (IUPAC)	Chemical Structure	Molecular Formula	UNODC EWA on NPS (May 2025)	CFSRE (NPS Discovery) (December 2024)	INCB Yellow list (July 2024)	INCB Green list (January 2025)	Unique to NPSfinder® database
**1**	1-phenethyl-4-hydroxypiperidine		1-(2-phenylethyl) piperidin-4-ol	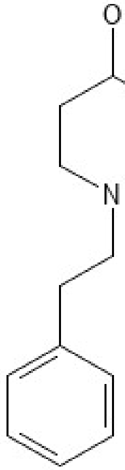	C13H19NO	**N**	**N**	**N**	**N**	**Y**
**2**	4-cyano-2-dimethylamino-4,4-diphenylbutane	methadone intermediate; premethadone	4-(dimethylamino)-2,2-diphenylpentanenitrile	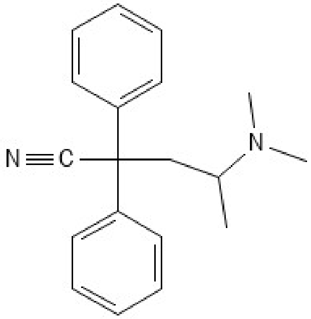	C19H22N2	**N**	**N**	**Y**	**N**	**N**

**3**	4-phenylpiperidine-4-carboxylic acid	Norpethidinic acid; Normeperidinic acid	4-phenylpiperidine-4-carboxylic acid	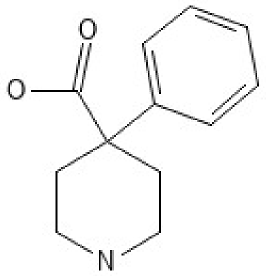	C12H15NO2	**N**	**N**	**N**	**N**	**Y**
**4**	Dextrorphan	DXO; Levorphanol d-form	(1*S*,9*S*,10*S*)-17-methyl-17-azatetracyclo[7.5.3.0[1,10].0[2,7]]heptadeca-2(7),3,5-trien-4-ol	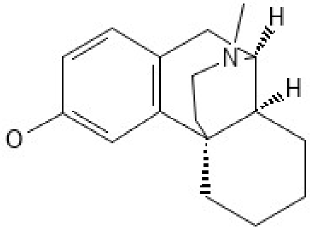	$C_{17}H_{23}NO$	**N**	**N**	**N**	**N**	**Y**
**5**	Morphine-3-B-D-Glucoride-N-oxide	Morphine 3-β-D-glucuronide-N-Oxide	N.A	N.A	N.A	**N**	**N**	**N**	**N**	**Y**

**6**	Morphine-3-B-D-Glucuronide	Morphine-3-glucuronide; Morphine-3-beta-D-glucuronide	(2S,3S,4S,5R,6S)-6-[[(4R,4aR,7S,7aR,12bS)-7-hydroxy-3-methyl-2,4,4a,7,7a,13-hexahydro-1H-4,12-methanobenzofuro[3,2-e]isoquinolin-9-yl]oxy]-3,4,5-trihydroxyoxane-2-carboxylic acid	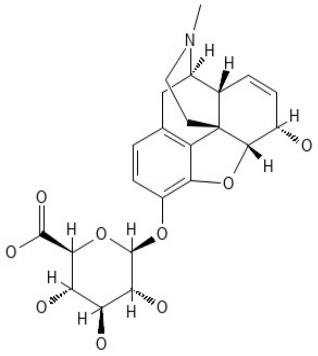	$C_{23}H_{27}NO_9$	**N**	**N**	**N**	**N**	**Y**
**7**	Morphine-6-B-D-glucuronide-N-oxide		N.A	N.A	N.A	**N**	**N**	**N**	**N**	**Y**
**8**	Normorphine hemisuccinide		N.A	N.A	N.A	**N**	**N**	**N**	**N**	**Y**

**9**	NPP	1-Phenethyl-4-piperidone; N-Phenethyl-4-piperidinone	1-(2-phenylethyl)piperidin-4-one	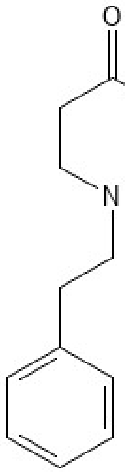	$C_{13}H_{17}NO$	**N**	**N**	**N**	**N**	**Y**
**10**	oxymorphone-3-glucuronide		(2S,3S,4S,5R,6S)-6-[[(4R,4aS,7aR,12bS)-4a-hydroxy-3-methyl-7-oxo-2,4,5,6,7a,13-hexahydro-1H-4,12-methanobenzofuro[3,2-e]isoquinolin-9-yl]oxy]-3,4,5-trihydroxyox	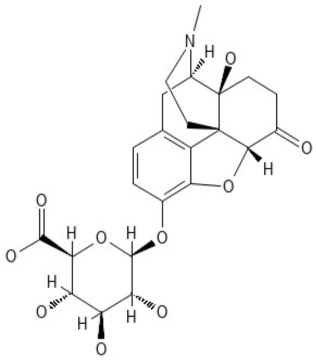	$C_{23}H_{27}NO_{10}$	**N**	**N**	**N**	**N**	**Y**

			ane-2-carboxylic acid							
**11**	Pethidine intermediate A		1-Methyl-4-phenylpiperidine-4-carbonitrile	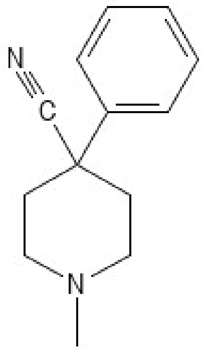	$C_{13}H_{16}N_2$	**N**	**N**	**Y**	**N**	**N**
**12**	Pethidinic Acid	meperidinic acid; pethidine intermediate C	1-methyl-4-phenylpiperidine-4-carboxylic acid	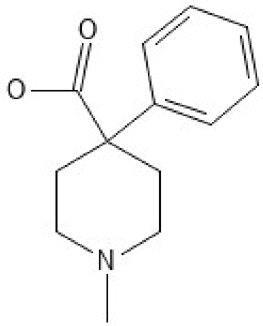	$C_{13}H_{17}NO_2$	**N**	**N**	**N**	**N**	**Y**

Table S2Cc – NPSfinder®: miscellaneous opioids; e.g., non fentanyl precursors; intermediates; inactive metabolites; comparison between the different databases. NPSfinder® crawling in 2018. (updated table from Arillotta et al., 2020, Table 2C)

N	Molecule denomination in NPS*finder*®	Other Names	Chemical Name (IUPAC)	Chemical Structure	Molecular Formula	UNODC EWA on NPS (May 2025)	CFSRE (NPS Discovery) (December 2024)	INCB Yellow list (July 2024)	INCB Green list (January 2025)	Unique to NPS*finder*® database
**1**	1-Phenethyl-4-propionyloxypiperidine		1-(2-Phenylethyl)-4-(propionyloxy)piperidine	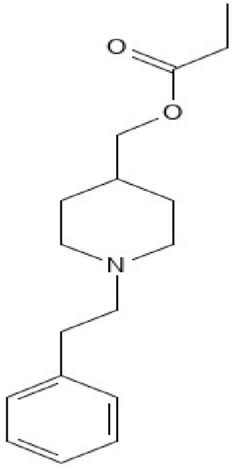	C16H23NO2	**N**	**N**	**N**	**N**	**Y**

**2**	1-Propionyl-4-anilinopiperidine		1-(4-anilinopiperidin-1-yl)propan-1-one	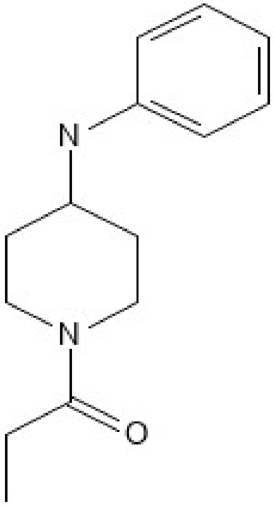	$C_{14}H_{20}N_2O$	**N**	**N**	**N**	**N**	**Y**
**3**	4-ANBocP		*tert*-butyl 4-anilinopiperidine-1-carboxylate	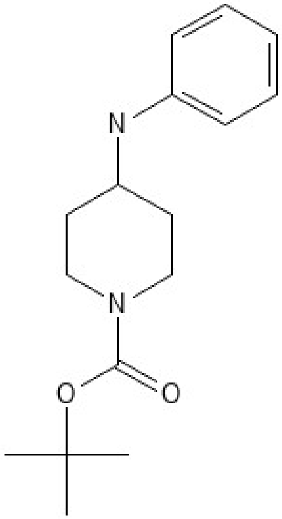	$C_{16}H_{24}N_2O_2$	**N**	**N**	**N**	**N**	**Y**

**4**	4-chloro-4-ANPP	Despropionyl para-Chlorofentanyl	*N*-(4-chlorophenyl)-1-(2-phenylethyl)piperidin-4-amine	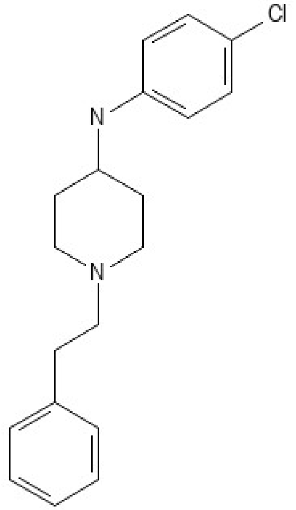	$C_{19}H_{23}ClN_2$	**N**	**N**	**N**	**N**	**Y**
**5**	4F-Phenethyl-4-ANPP		N.A	N.A	N.A	**N**	**N**	**N**	**N**	**Y**

**6**	Fluoro-norfentanyl	para-fluoro Norfentanyl	N-(4-fluorophenyl)-N-piperidin-4-ylpropanamide	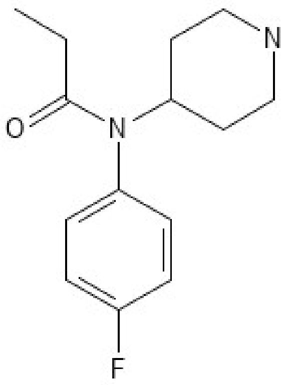	$C_{14}H_{19}FN_2O$	**N**	**N**	**N**	**N**	**Y**
**7**	iso-Butanoyl-4-fluorofentanyl		*N*-(1-benzylpiperidin-4-yl)-*N*-(4-fluorophenyl)-2-methylpropanamide	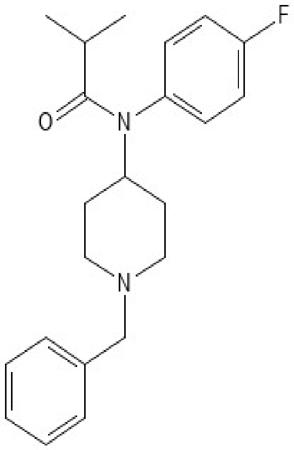	$C_{22}H_{27}FN_2O$	**N**	**N**	**N**	**N**	**Y**

**8**	N-Boc Norfentanyl		*tert*-butyl 4-(*N*-propanoylanilino)piperidine-1-carboxylate	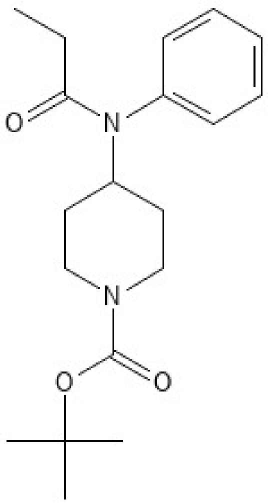	$C_{19}H_{28}N_2O_3$	**Y**	**N**	**N**	**N**	**N**
**9**	N-Propionylnorfentanyl		N.A	N.A	N.A	**N**	**N**	**N**	**N**	**Y**
**10**	Norfentanyl		*N*-phenyl-*N*-piperidin-4-ylpropanamide	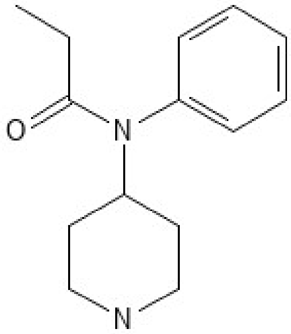	$C_{14}H_{20}N_2O$	**N**	**N**	**N**	**N**	**Y**

**11**	Phenethyl 4-ANPP		*N*-phenyl-*N*,1-bis(2-phenylethyl)piperidin-4-amine	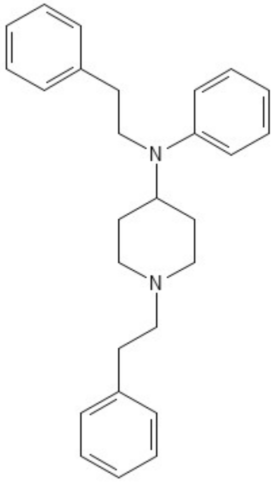	$C_{27}H_{32}N_2$	**N**	**N**	**N**	**N**	**Y**
**12**	Propionyl-fluoronorfentanyl		*N*-(4-fluorophenyl)-*N*-(1-propanoylpiperidin-4-yl)propanamide	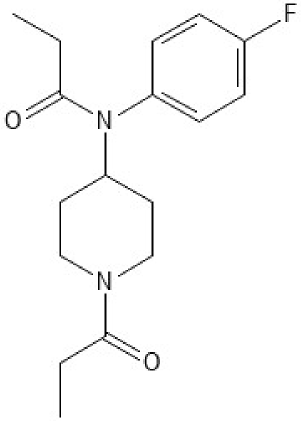	$C_{17}H_{23}FN_2O_2$	**N**	**N**	**N**	**N**	**Y**

Table S3A NPSfinder®: fentanyl analogues and precursor-like compounds; comparison between the different databases. NPSfinder® crawling in 2023.

N	Molecule denomination in NPS*finder*®	Other Names	Chemical Name (IUPAC)	Chemical Structure	Molecular Formula	UNODC EWA on NPS (May 2025)	CFSRE (NPS Discovery) (December 2024)	INCB Yellow list (July 2024)	INCB Green list (January 2025)	Unique to NPS*finder*® database
**1**	Acetylcodeine	6-Acetylcodeine	[(4*R*,4*aR*,7*S*,7*aR*,12*bS*)-9-methoxy-3-methyl-2,4,4*a*,7,7*a*,13-hexahydro-1*H*-4,12-methanobenzofuro[3,2-e]isoquinolin-7-yl] acetate	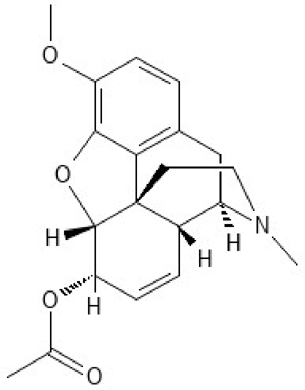	$C_{20}H_{23}NO_4$	**N**	**N**	**N**	**N**	**Y**
**2**	Mitragynine		Methyl (*E*)-2-[(2*S*,3*S*,12*bS*)-3-ethyl-8-methoxy-1,2,3,4,6,7,12,12*b*-octahydroindolo[2,3-a]quinolizin-2-yl]-3-methoxyprop-2-enoate	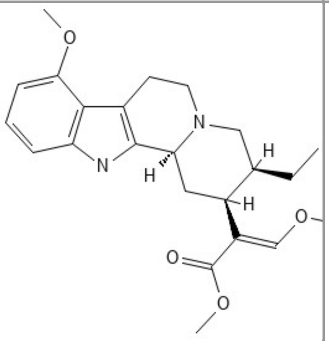	$C_{23}H_{30}N_2O_4$	**N**	**N**	**N**	**N**	**Y**

**3**	Dipyanone		4,4-diphenyl-6-pyrrolidin-1-ylheptan-3-one	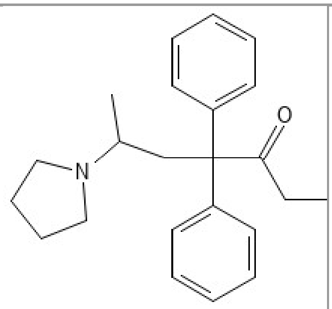	$C_{23}H_{29}NO$	**Y**	**Y**	**N**	**N**	**N**
**4**	Etonitazepyne	n-pyrrolidino etonitazene	2-[(4-ethoxyphenyl)methyl]-5-nitro-1-(2-pyrrolidin-1-ylethyl)benzimidazole	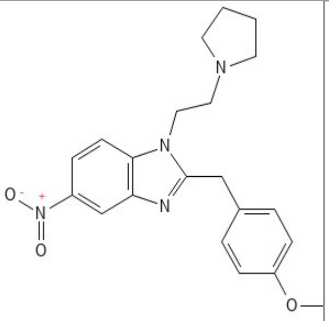	$C_{22}H_{26}N_4O_3$	**Y**	**N**	**Y**	**N**	**N**
**5**	Hydrocotarnine	4-methoxy-6-methyl-2H,5H,6H,7H,8H-[1,3]dioxolo[4,5-g]isoquinoline	4-methoxy-6-methyl-7,8-dihydro-5*H*-[1,3]dioxolo[4,5-g]isoquinoline	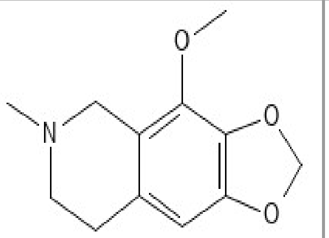	$C_{12}H_{15}NO_3$	**N**	**N**	**N**	**N**	**Y**

**6**	Metonitazene		*N*,*N*-diethyl-2-[2-[(4-methoxyphenyl)methyl]-5-nitrobenzimidazol-1-yl]ethanamine	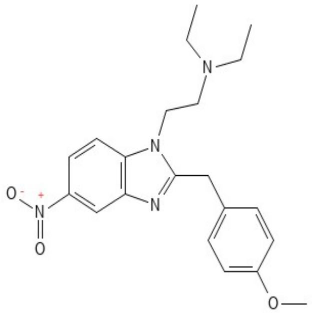	$C_{21}H_{26}N_4O_3$	**Y**	**Y**	**Y**	**N**	**N**
**7**	N-Cyclohexylbenzamide	Hexamethylenebenzamide	*N*-cyclohexylbenzamide	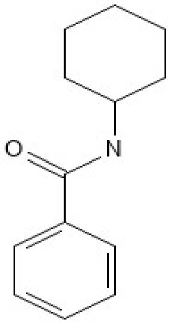	$C_{13}H_{17}NO$	**N**	**N**	**N**	**N**	**Y**
**8**	N-Desethylisotonitazene		*N*-ethyl-2-[5-nitro-2-[(4-propan-2-yloxyphenyl)methyl]benzimidazol-1-yl]ethanamine	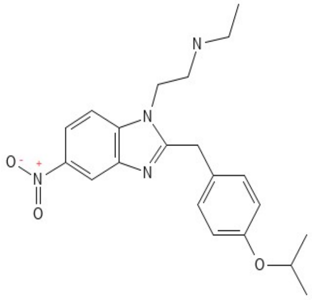	$C_{21}H_{26}N_4O_3$	**Y**	**Y**	**Y**	**N**	**N**

**9**	N-Phenylpropanamide		*N*-phenylpropanamide	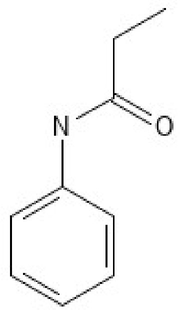	$C_9H_{11}NO$	**N**	**N**	**N**	**N**	**Y**
**10**	N-Piperidinyl Etonitazene	Etonitazepipne	2-[(4-ethoxyphenyl)methyl]-5-nitro-1-(2-piperidin-1-ylethyl)benzimidazole	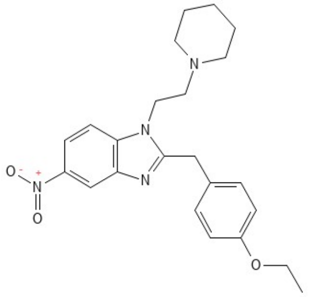	$C_{23}H_{28}N_4O_3$	**Y**	**Y**	**Y**	**N**	**N**
**11**	Protonitazene		*N*,*N*-diethyl-2-[5-nitro-2-[(4-propoxyphenyl)methyl]benzimidazol-1-yl]ethanamine	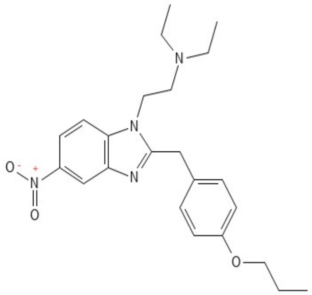	$C_{23}H_{30}N_4O_3$	**Y**	**Y**	**Y**	**N**	**N**

Table S3B – NPSfinder®: non fentanyl analogues (e.g., nitazene-like molecules, kratom-related compounds and other miscellaneous opioids); comparison between the different databases. NPSfinder® crawling in 2023.
